# Differential Apicobasal VEGF Signaling at Vascular Blood-Neural Barriers

**DOI:** 10.1016/j.devcel.2014.06.027

**Published:** 2014-09-08

**Authors:** Natalie Hudson, Michael B. Powner, Mosharraf H. Sarker, Thomas Burgoyne, Matthew Campbell, Zoe K. Ockrim, Roberta Martinelli, Clare E. Futter, Maria B. Grant, Paul A. Fraser, David T. Shima, John Greenwood, Patric Turowski

**Affiliations:** 1 Department of Cell Biology, UCL Institute of Ophthalmology, University College London, 11-43 Bath Street, London EC1V 9EL, UK; 2 Department of Ocular Biology and Therapeutics, UCL Institute of Ophthalmology, University College London, 11-43 Bath Street, London EC1V 9EL, UK; 3 Cardiovascular Division, King’s College London, 150 Stamford Street, London SE1 9NH, UK; 4 Neurovascular Genetics Laboratory, Smurfit Institute of Genetics, Lincoln Place Gate, Trinity College, Dublin 2, Ireland; 5 Eugene and Marilyn Glick Eye Institute, Department of Ophthalmology, Indiana University School of Medicine, 1160 West Michigan Street, Indianapolis, IN 46202, USA

## Abstract

The vascular endothelium operates in a highly polarized environment, but to date there has been little exploration of apicobasal polarization of its signaling. We show that VEGF-A, histamine, IGFBP3, and LPA trigger unequal endothelial responses when acting from the circulation or the parenchymal side at blood-neural barriers. For VEGF-A, highly polarized receptor distribution contributed to distinct signaling patterns: VEGFR2, which was found to be predominantly abluminal, mediated increased permeability via p38; in contrast, luminal VEGFR1 led to Akt activation and facilitated cytoprotection. Importantly, such differential apicobasal signaling and VEGFR distribution were found in the microvasculature of brain and retina but not lung, indicating that endothelial cells at blood-neural barriers possess specialized signaling compartments that assign different functions depending on whether an agonist is tissue or blood borne.

## Highlights

• At blood-neural barriers, only abluminal (tissue-side) VEGF-A induces permeability
• Most VEGFR1 is localized on the luminal face of neural microvascular endothelium
• Most VEGFR2 is localized on the abluminal face of neural microvascular endothelium
• Luminal VEGFR1 stimulates Akt; abluminal VEGFR2 induces permeability via p38

## Graphical Abstract

**Figure:**
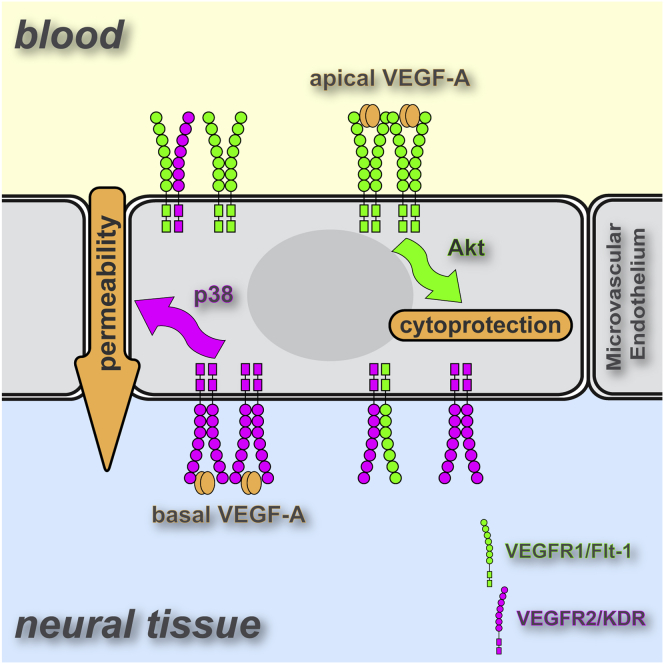

## Teaser

Hudson et al. show that the microvascular endothelium of blood-neural barriers uses functional polarity of signaling to compartmentalize responses to vasoactive stimuli. VEGFR2, predominantly expressed on the abluminal face of the endothelium, mediates p38 and vascular permeability. Luminally polarized VEGFR1 activates Akt but does not contribute to permeability.

## Introduction

Endothelial polarity is assumed to be mechanistically similar to that of epithelial cells, where it is well studied. Undoubtedly, the morphological and molecular organization of the vascular endothelial cells (ECs) at any of the diverse blood-tissue interphases must reflect the highly polarized environment in which they operate. For instance, vascular lumen formation is entirely dependent on segregation of apical and basal membrane compartments and subsequent EC polarization (Lizama and Zovein, 2013). A huge body of work has also demonstrated that at vascular blood-brain or blood-retinal barriers, neuronal homeostasis is maintained by highly polarized localization of transporters and channels, which regulate the directional movement of ions, drugs, metabolites, and toxins (Abbott et al., 2010). Thus, it is assumed that, in their naturally highly polarized environment, ECs have adopted differential apicobasal signaling. However, experimental proof of such polarized signaling processes is still missing.

Vascular endothelial growth factor (VEGF) family members, and in particular its most studied representative VEGF-A, are central to the creation of new blood vessels during normal development and growth but also in pathological situations such as tumorigenesis and ocular neovascular disease (Koch et al., 2011, Takahashi and Shibuya, 2005). Besides its angiogenic role, VEGF-A has additional effects on the vascular endothelium. It was originally discovered for its ability to trigger vascular permeability, and this permeability-enhancing property of VEGF-A is linked to interstitial fluid accumulation in tumors and psoriatic lesions, as well as tissue edema and concomitant vision loss in neovascular eye disease (Ferrara et al., 2007). VEGF-A also has beneficial roles in the systemic vasculature, regulating normal vascular tone and acting as a trophic factor for the vascular endothelium (Maharaj and D’Amore, 2007).

VEGF-A mediates its diverse functions through the use of multiple receptors, mainly the receptor tyrosine kinases VEGFR1 (Flt-1) and VEGFR2 (Flk-1/KDR) (Koch et al., 2011). The most common splice isoform VEGF-A(165), subject of the present study, additionally interacts with neuropilin 1 and various cell-surface heparan sulfate proteoglycans to modulate the intracellular response. VEGF-A belongs to a family of growth factors with five genes encoding VEGF-A, -B, -C, and -D and placental growth factors (PlGFs), each binding VEGFRs with different affinities. For instance, PlGF-1 and the viral gene product VEGF-E specifically bind and activate VEGFR1 and VEGFR2, respectively (Takahashi and Shibuya, 2005).

VEGF-A triggers a plethora of intracellular signaling steps in ECs (Koch et al., 2011). Standout mediators associated with the VEGF-A-induced endothelial permeability response are the mitogen-activated protein kinases Erk and p38, phospholipases, protein kinase C, phosphatidylinositol 3-kinase (PI3K)-activated Akt, and endothelial nitric oxide synthase (eNOS) (Weis and Cheresh, 2005). However, given the complexity of VEGF-A signaling and the permeability response, as well as the morphological and cytological differences of various endothelia, it is not surprising that these signaling components remain under intense scrutiny, in particular with respect to their specific role in particular experimental models and their interaction with each other in controlling various effector mechanisms.

Because of VEGF-A’s central role in the regulation of endothelial functions in health and disease, anti-VEGFs have become irreplaceable tools in treating pathological angiogenesis and permeability (Ferrara et al., 2007). However, systemic anti-VEGF-A therapies are associated with endothelial dysfunction leading to bleeding, inflammation, hypertension, proteinuria, and even lethality (Chen and Cleck, 2009). Consistent with these observations in patients, animal models demonstrate that VEGF-A is constitutively required to maintain vascular tone and vascular EC survival and that reductions in plasma VEGF-A levels cause vascular attrition and functional abnormalities (Sugimoto et al., 2003).

We therefore hypothesized that circulating and tissue-produced VEGF-A induce distinct responses in ECs. In light of the central role of VEGF-A as a permeability-inducing factor in brain (Merrill and Oldfield, 2005, Argaw et al., 2012) and eye pathologies (Miller et al., 2013), our study focused on vascular ECs at blood-neural barriers and on acute endothelial permeability (as opposed to chronic interstitial fluid accumulation during inflammation, cancer, and wound healing, which may involve cells other than ECs) (Nagy et al., 2008). Our study is also restricted to the in vitro and in vivo analysis of the microvasculature as opposed to the macrovasculature, because this is where physiological and pathological vascular permeability occurs. Here we report that VEGF-A induces unequal responses depending on whether it acts on the luminal or abluminal side of neural microvascular ECs (MVECs). Mechanistically, this differential EC response is predicated on polarized VEGFR expression and distinct downstream signaling.

## Results

**Figure 1:**
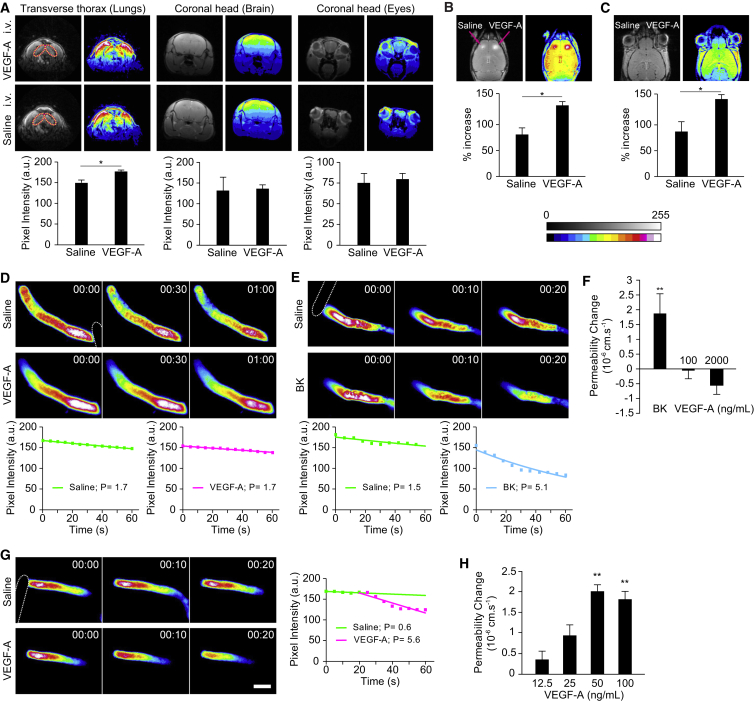
Abluminal but Not Luminal VEGF-A Induces Permeability In Vivo (A–C) T-1-weighted MRI in mice showed increased extravasation of Gd-DTPA (742 Da) in the lungs (circled) but not the brain or eyes in response to i.v. VEGF-A (3 μg/animal) (A). In contrast, direct injection of VEGF-A (ca. 8 ng) into the brain cortex (B; arrows indicate injection sites) or the vitreous of the eye (C) led to increased extravasation. Shown are representative contrast-enhanced and 16-color pseudocolored images taken 8 min after VEGF-A injection, with densitometric quantification of each experimental group shown beneath. a.u., arbitrary units. (D and E) Time-dependent recording of sulforhodamine B (580 Da) loss from single occluded rat pial microvessels in vivo showed no change of permeability in response to an intracarotid (i.e., luminal) bolus injection of VEGF-A (100 ng/ml) (D). In contrast, luminal bradykinin (BK) at 10 μM induced a rapid loss of dye (E). Shown are micrographs of pseudocolored microvessels at indicated times (min:s) after either saline or VEGF-A or bradykinin injection. The dotted outline in the first image in each series indicates the position of the occluding probe. Densitometric fluorescence intensities were plotted against time, and permeability values P (10^−6^ cm/s) were calculated by fitting data to the equation C_t_ = C_0_ e^−kt^, where k = 4P/d and d is the diameter of the vessel. (F) Mean permeability changes determined as described in (D) and (E) in response to either 10 μM bradykinin or 100 or 2,000 ng/ml VEGF-A. (G) Application of VEGF-A (100 ng/ml) to the abluminal, extravascular space of single pial microvessels in vivo produced a strong increase of permeability to sulforhodamine B (580 Da). The scale bar represents 50 μm. (H) Mean permeability changes in response to increasing concentration of VEGF-A. Data are means ± SEM of at least three independent experiments. ^∗∗^p < 0.01, ^∗∗∗^p < 0.001 [Student’s t test (A–C) and ANOVA and Dunnett’s post hoc test (F and H)].

Using contrast-enhanced magnetic resonance imaging (MRI) in the mouse, we determined tissue extravasation of intravenous (i.v.) injected gadolinium (gadolinium diethylenetriamine pentaacetic acid [Gd-DTPA], 742 Da). Basal leakage of Gd-DTPA was significantly higher in peripheral tissues such as the lung than the brain or the eye, reflecting the significant difference in baseline permeability between neural and nonneural vascular beds. Intravenous injection of VEGF-A (3 μg/mouse) significantly enhanced leakage of Gd-DTPA into lungs within 10 min (Figure 1A), in agreement with previous reports of VEGF-A-induced permeability in nonneural vasculature, such as that of the trachea or the mesentery (Sun et al., 2012, Bates and Curry, 1996). Significantly, no leakage was observed in the brain or the eye over a 30 min observation period. However, when VEGF-A was directly injected into the brain cortex or the vitreous of the eye, rapid and significant accumulation of Gd-DTPA in the vicinity of the injection site was observed (Figures 1B and 1C), indicating that only tissue-borne but not circulating VEGF-A induced vascular hyperpermeability in the neural tissues.

This was further studied in single pial microvessels, which exhibit barrier properties closely resembling those found at the intact blood-brain barrier (BBB) and allow permeability measurements that are tightly controlled with respect to kinetics and dose (Butt et al., 1990, Easton et al., 1997). Increasing VEGF-A levels up to 2 μg/ml within the lumen of tight pial microvessels did not affect baseline permeability (Figures 1D and 1F), even when bradykinin triggered strong vascular permeability (Figures 1E and 1F). In contrast, exposure to VEGF-A from the tissue side led to a rapid increase of permeability (Figures 1G and 1H), which was maximal at a concentration of ca. 50 ng/ml. Taken together, these data demonstrated that cerebral microvascular permeability is strongly induced by VEGF-A, albeit only when presented to the abluminal face of microvessels.

**Figure 2:**
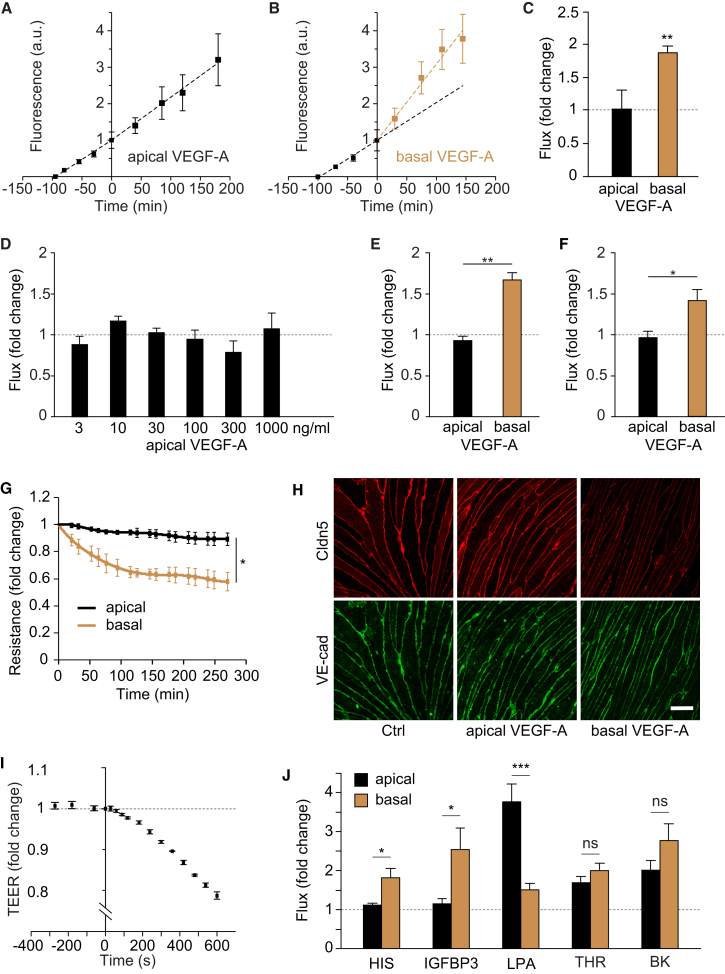
Basal, but Not Apical, VEGF-A Induces Permeability in Cultured Cerebral or Retinal MVECs (A–C) Rat cerebral MVECs were grown on permeable Transwell inserts until they reached a TEER of greater than 200 Ω/cm^2^. FITC dextran (4 kDa) was added to the apical chamber and flux was measured as the time-dependent accumulation of fluorescence in the basal chamber. VEGF-A (50 ng/ml) addition to the apical side of the cells (at time 0) did not change flux rates (A). In contrast, a rapid change in flux was observed when it was added to the basal side of the cells (B). Dotted lines are linear regressions of data points before and after the addition of VEGF-A. Mean fold changes of flux following apical or basal application are shown in (C). (D) Flux was measured as in (A)–(C) in the presence of apical VEGF-A at the indicated concentrations, none of which led to significant flux changes. (E and F) Basal but not apical VEGF-A (50 ng/ml) also induced significant 4 kDa FITC dextran flux changes in postconfluent rat (E) or porcine (F) retinal MVECs. (G) Postconfluent rat cerebral MVECs grown on permeable Transwell inserts were subjected either apically or basally to 50 ng/ml VEGF-A, and time-dependent changes in electrical resistance were monitored by impedance spectroscopy. (H) Changes in the distribution of Cldn5 and VE-cadherin in response to either apical or basal VEGF-A (50 ng/ml, 1 hr) were analyzed by confocal microscopy in postconfluent rat cerebral MVECs. Shown are representative projections spanning the entire thickness of the monolayers. The scale bar represents 10 μm. (I) As in (G), except that direct TEER changes were measured every ca. 30 s using chopstick electrodes. VEGF-A (50 ng/ml) was added to the basal side at time 0. (J) Transendothelial flux was measured in postconfluent primary rat brain MVECs in response to apical or basal histamine (100 μM; HIS), IGFBP3 (50 ng/ml), LPA (10 μM), thrombin (1 U/ml; THR), or bradykinin (10 μM; BK). Data are means ± SEM of at least three independent experiments. ^∗^p < 0.05, ^∗∗^p < 0.01, ^∗∗∗^p < 0.001; ns, not significant [Student’s t test (C, E, F, and J), ANOVA (D), or two-factor analysis of variance (location of VEGF application by time) with one repeated measure (time) (G)]. Note that in (J), only p values of apical versus basal treatments are shown. See also Figure S1.

The microvasculature in the brain and retina constitutes a network of ECs tightly interacting with mural cells such as pericytes and glial cells including astrocytes and Müller cells (Abbott et al., 2010). Because microvascular permeability operates primarily on the level of endothelium, we tested whether the ECs themselves responded to VEGF in a polarized fashion. For this, we used highly purified nonpassaged, primary MVECs isolated from brain or retina, which were characterized by preservation of interendothelial junctions, apicobasal polarity, and very good in vitro barrier properties (Figure S1 available online). Macromolecular apical-to-basal flux was enhanced nearly 2-fold when 50 ng/ml VEGF-A was added to the basal side of rat brain MVEC monolayers, whereas doses of up to 1 μg/ml applied to the apical side did not change flux (Figures 2A–2D). A similar, polarized response to VEGF-A was also observed in cultures of murine brain (data not shown) and rat or porcine retinal MVECs (Figures 2E and 2F), suggesting that functional polarity operates at all vascular blood-brain and -retinal barriers and across species. Importantly, both barrier properties and the sided response to VEGF-A were lost following repeated subculturing of MVECs (Figures S1D–S1F), offering a plausible explanation as to why this phenomenon has not yet been universally observed. VEGF-A also induced changes in transendothelial electrical resistance (TEER) and significant reduction in junctional claudin 5 (Cldn5) (Figures 2G and 2H), a reliable molecular indicator of MVEC barrier and BBB integrity (Nitta et al., 2003), indicating that paracellular rather than transcellular permeability was operational (Steed et al., 2010). VEGF-A provoked flux and TEER changes within minutes (Figures 2B and 2I), thus resembling the acute response observed in vivo. A sided permeability response similar to that with VEGF-A was also observed with histamine and insulin-like growth factor-binding protein 3 (IGFBP3) (Figure 2J), whereas opposite responsiveness was observed with lysophosphatidic acid (LPA). Thrombin and bradykinin induced significant flux in brain MVECs but without predilection for either endothelial face. Significantly, the sided response to histamine and LPA was consistent with previous results from pial microvessels in vivo (Sarker et al., 1998, Sarker et al., 2010).

**Figure 3:**
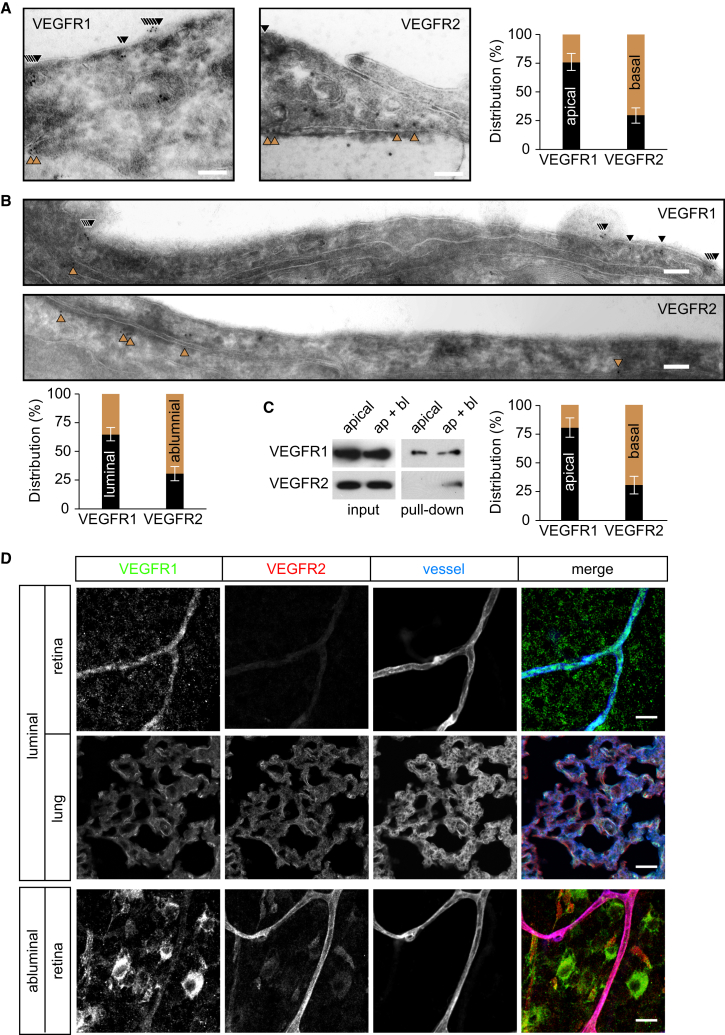
Differential VEGF Receptor Localization in Microvascular ECs in Brain and Retina (A and B) Cryoimmunogold EM analysis of VEGFR1 and -R2 in primary rat brain MVECs (A) or mouse hippocampal microvessels (B) showed predominant apical/luminal (black) and basal/abluminal (brown) localization, respectively. Mean distribution of each VEGF receptor (±SEM) was determined by quantifying gold particles located within ca. 20 nm of the plasma membrane (as indicated by arrowheads) in five independent sections, each comprising at least 10 μm of continuous plasma membrane. The scale bars represent 100 nm. (C) VEGF receptor distribution was also analyzed in postconfluent rat cerebral MVECs by biotinylation of either the apical or, in the presence of EDTA, the apical and basal membranes (ap+bl). Biotinylated proteins were isolated and analyzed by western blot. Shown are representative immunoblots of VEGFR1 and VEGFR2 and quantitative distribution analysis from three experiments (means ± SEM). (D) VEGFR1 but not -R2 antibodies bound to the retinal vasculature of mice within 5 min of luminal delivery through cardiac injection. However, in the same animals, both VEGFR1 and -R2 antibodies were found bound to alveolar microvessels in the lung. Inversely, when unfixed, nonpermeabilized retinae were incubated with VEGFR antibodies (abluminal), only VEGFR2 was found to stain microvessels significantly. All whole mounts and sections were counterstained with the vessel marker isolectin B4 and analyzed by confocal microcopy. Shown are optical sections of ca. 8 μm thickness of the retinal ganglion cell layer or the center of the lung. The scale bars represent 20 μm. See also Figure S2 and Table S1.

One explanation for the polarized responses to VEGF-A is that distinct signaling pathways, and in particular different VEGF-A receptors, operate on the apical and basal sides of brain or retinal MVECs. To test this hypothesis, we studied the distribution of VEGFR1 (Flt-1) and VEGFR2 (KDR/Flk-1) (Koch et al., 2011). Cryoimmunogold electron microscopy (EM) showed that ca. 75% of VEGFR1 was found apically in rat brain MVECs, whereas ca. 70% of VEGFR2 was found at basal membranes (Figure 3A; Table S1). Analysis by domain-specific biotinylation (Figure 3C) or analysis by confocal microscopy (Figures S2A and S2B) confirmed this highly polarized distribution pattern. Importantly, distribution of VEGFR1 and VEGFR2 was also predominantly luminal and abluminal, respectively, in the microvascular endothelium of the mouse hippocampus (Figure 3B; Table S1). VEGF receptor distribution was also interrogated in vivo: the luminal side of the mouse vasculature was exposed for 5 min to anti-VEGFR1 or -R2 antibodies by cardiac injection. Following perfusion, VEGFR antibody binding was studied by fluorescent secondary antibody labeling of retinae and lungs (Figure 3D; Figure S2C). VEGFR1 antibodies bound to retinal and lung microvessels, whereas VEGFR2 antibodies strongly labeled lung but not retinal microvessels. In contrast, when the same antibodies were applied abluminally for 5 min to unfixed and nonpermeabilized retinae, VEGFR2 but not -R1 was detectable in retinal vessels. In addition, both antibodies strongly reacted with other cells in the retinal ganglion cell layer, consistent with the reported expression of VEGF receptors in ganglion and Müller cells (Saint-Geniez et al., 2008, Foxton et al., 2013). Taken together, the majority of VEGFR1 and -R2 was found to be inversely located to the luminal and abluminal sides, respectively, of MVECs in the brain and the retina but not the lung.

**Figure 4:**
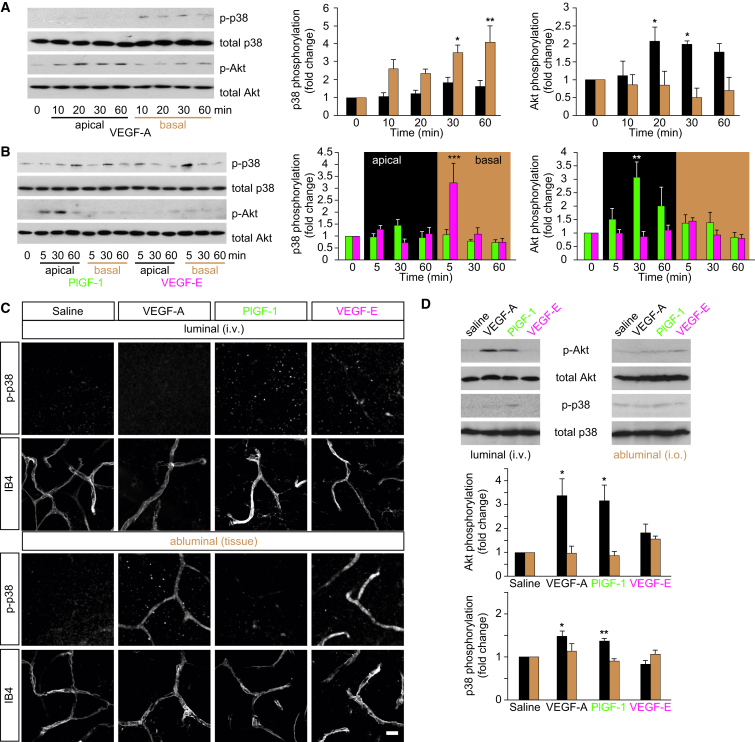
Differential VEGF Receptor Signaling in Brain MVECs and Neural Microvessels (A) Basal but not apical VEGF-A (50 ng/ml) induced significant activation of p38 but not Akt in postconfluent primary rat brain MVECs. In contrast, Akt but not p38 was activated in response to apical VEGF-A. At the indicated times of VEGF-A stimulation, whole-cell lysates were prepared and levels of phosphorylated p38 (pT180/Y182) and Akt (pS473) were determined by western blotting. Shown are representative blots and normalized densitometric quantifications. (B) Kinase response to PlGF-1 or VEGF-E analyzed as in (A). p38 was activated within 5 min of basal but not apical VEGF-E (50 ng/ml) treatment, whereas slower Akt activation was seen in response to apical but not basal PlGF-1 (50 ng/ml). (C) Intravenously injected VEGF-A, PlGF-1, or VEGF-E (at 120 μg/kg) (luminal) did not induce activation of p38 in the pial vasculature of P23 rats. When applied abluminally to the pial microvasculature (at 100 ng/ml) (abluminal), VEGF-A and VEGF-E but not PlGF-1 led to rapid activation of p38. Pial tissues were fixed within 5 min of treatment and then stained for phosphorylated p38 (pT180/Y182). All sections were counterstained with the vessel marker isolectin B4 (IB4) and analyzed by confocal microscopy. Shown are projections spanning a thickness of ca. 11 μm. The scale bar represents 20 μm. (D) VEGF-A, PlGF-1, or VEGF-E was either injected into the tail vein of P23 rats (at 120 μg/kg) or into the vitreous (i.o.; 100 ng/eye). After ca. 20 min, retinae were isolated and subjected to quantitative immunoblot analysis as described for (A) and (B). Akt was robustly activated in response to i.v. but not i.o. injected VEGF-A and PlGF-1. p38 was weakly activated as well. VEGF-E did not induce any significant effects. Shown are means ± SEM from at least three independent experiments. ^∗^p < 0.05, ^∗∗^p < 0.01, ^∗∗∗^p < 0.001 [ANOVA and Dunnett’s post hoc test (A, B, and D)]. See also Figure S3.

These data strongly suggested that different downstream signals operate in response to apical or basal VEGF. Indeed, we found that the time-dependent activation of p38 and Akt was significantly different following apical or basal exposure of brain MVECs to VEGF-A. Phosphorylation of p38, increasing over time, was observed in response to basal but not apical VEGF-A (Figure 4A). In contrast, Akt activation was only seen following apical exposure to VEGF-A. Apical but not basal exposure to PlGF-1, which selectively binds to VEGFR1 (Takahashi and Shibuya, 2005), also induced Akt phosphorylation, with a peak at ca. 30 min (Figure 4B). In contrast, stimulation with the VEGFR2-selective ligand VEGF-E (Takahashi and Shibuya, 2005) did not lead to Akt activation from any side of the ECs. Instead, strong phosphorylation of p38 was observed within 5 min of basal but not apical exposure.

The hallmarks of this differential p38 and Akt activation pattern could be recapitulated by VEGF treatment in vivo. In rats, intravenous injection of neither VEGF-A, PlGF-1, nor VEGF-E (120 μg/kg) led to any activation of p38 in pial microvessels (Figure 4C). Nevertheless, in alveolar lung vessels of the same animals, p38 was strongly and rapidly activated in response to i.v. VEGF-A or VEGF-E but not PlGF-1 (Figure S3). In pial microvessels, VEGF-A and VEGF-E led to rapid p38 activation, but only when growth factors were administered from the tissue side through a cranial window (Figure 4C). We also attempted to study Akt activation in a similar manner. However, none of six different commercial anti-phospho-Akt antibodies produced significantly altered staining patterns in brain, lung, or retinal tissues of rats treated with time courses of luminal or abluminal VEGFs or indeed insulin, suggesting that reliable phospho-Akt detection was not possible by immunohistochemistry (data not shown). We therefore opted to study Akt activation in whole-cell lysates of retinal tissue. Intravenous injection of VEGF-A or PlGF-1 but not VEGF-E led to strong Akt activation after 25 min (Figure 4D). Some weak activation of p38 was also observed under these conditions. Direct injection of VEGFs into the retina (i.e., abluminal application) did not lead to any significant Akt phosphorylation. Taken together, these data demonstrated that in cerebral or retinal MVECs, rapid p38 activation occurred downstream of abluminal VEGFR2. In contrast, Akt activation occurred more slowly and downstream of luminal VEGFR1.

**Figure 5:**
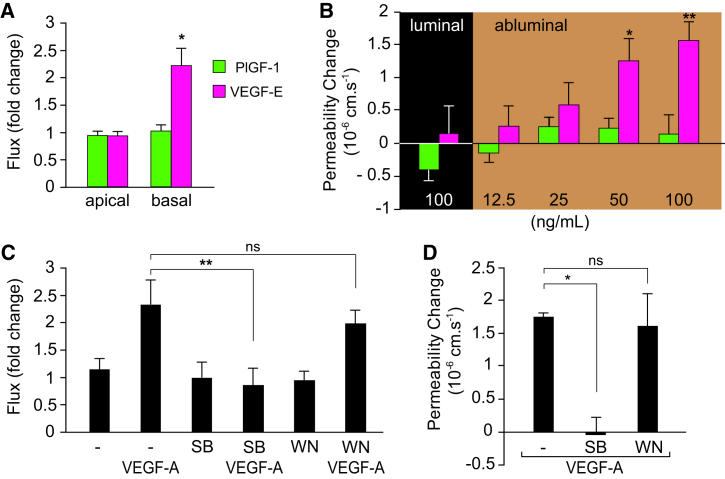
VEGFR2 and p38 Mediate the Brain Microvascular Permeability Response (A) Transendothelial flux of 4 kDa dextran across confluent primary rat brain MVECs was increased in response to basal but not apical VEGF-E (50 ng/ml). PlGF-1 (50 ng/ml) did not affect flux. (B) Application of VEGF-E but not PlGF-1 to the abluminal, extravascular space of single pial microvessels in vivo produced a dose-dependent increase of permeability to sulforhodamine B (580 Da). Intracarotid (i.e., luminal) bolus injection of PlGF-1 or VEGF-E did not affect permeability. (C) Flux measurements in primary brain MVECs showed that pretreatment with 10 μM p38 inhibitor SB202190 (SB) but not the PI3K inhibitor wortmannin (WN) inhibited the permeability response to 50 ng/ml VEGF-A. (D) VEGF-A-induced permeability changes in pial microvessels were abolished by pretreatment with 10 μM SB202190 but not wortmannin. Shown are means ± SEM from at least three independent experiments. ^∗^p < 0.05, ^∗∗^p < 0.01; ns, not significant [Student’s t test (A), ANOVA and Dunnett’s post hoc test (B and D), and two-way ANOVA and Bonferroni’s post hoc test (C)]. See also Figure S4.

This polarized signaling pattern was not only in full agreement with the VEGFR1 and -R2 localization described in Figure 3 but also with the endothelial permeability response. Enhanced macromolecular flux across brain MVECs was seen following basal (but not apical) VEGF-E stimulation, whereas PlGF-1 had no effect on barrier function, neither in vitro nor in vivo (Figures 5A and 5B). Furthermore, we found that SB202190, but not wortmannin or LY294002, which inhibited VEGF-induced p38 or Akt activation, respectively (Figures S4A and S4B), abrogated VEGF-induced hyperpermeability in vitro and in vivo (Figures 5C and 5D; Figure S4C). Taken together, this clearly indicated that the permeability response to VEGF at blood-neural barriers is mediated by abluminal VEGFR2. In agreement, the VEGFR2 inhibitor SU1498 completely abolished VEGF-A-induced flux in brain MVECs (Figure S4C).

**Figure 6:**
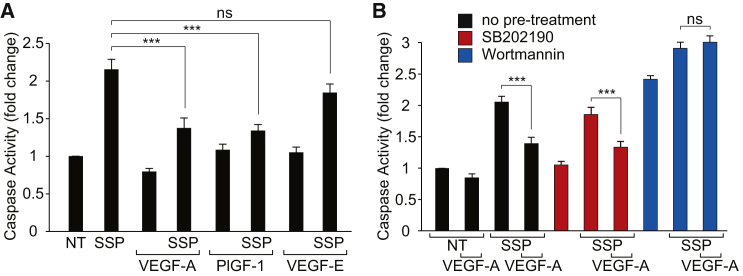
VEGFR1 and Akt-Mediated Cytoprotection in Brain Microvascular ECs (A) Staurosporine (SSP; 1 μM, 60 min) more than doubled caspase 3/7 activity in primary rat brain MVECs when compared to untreated control cells (NT). The staurosporine-induced caspase 3/7 activity was significantly reduced by pretreatment with VEGF-A and PlGF-1 but not VEGF-E (all at 50 ng/ml for 30 min). (B) The VEGF-A-mediated reduction of staurosporine-induced caspase 3/7 activity was sensitive to 10 μM PI3K inhibitor wortmannin but not the p38 inhibitor SB202190. Shown are means ± SEM (n ≥ 15). ^∗∗∗^p < 0.001; ns, not significant (Student’s t test).

Abundant data suggest that systemic (hence, luminal) VEGF-A is a trophic factor for the vasculature (Maharaj and D’Amore, 2007, Gerber et al., 1998). In light of the specific activation of Akt following luminal VEGFR1 stimulation, we investigated a potential role in endothelial cytoprotection. Rat brain MVECs were stimulated with the microbial alkaloid staurosporine, widely used to induce apoptosis in most cell types, including ECs (Kabir et al., 2002). Apoptosis, measured as an increase in caspase 3/7 activity, was significantly reduced by apical VEGF-A or PlGF-1 but not VEGF-E (Figure 6A). This VEGF-mediated cytoprotection was sensitive to wortmannin but not to SB202190 (Figure 6B). To this end, a potential role of VEGF in cytoprotection of retinal ECs could not be interrogated in vivo, because staurosporine was ineffective in inducing EC death. Staurosporine was applied to mouse or rat retinae both intravitreally and through the carotid artery at up to 40 μM for up to 12 hr. This treatment induced severe retinal ganglion cell death but did not affect ECs at all (data not shown). Even when additionally circulating VEGF-A was reduced by coadministration of anti-VEGF antibodies, we could not measure any annexin V binding or activated caspase 3 staining in retinal ECs (data not shown). Thus, VEGF mediates a cytoprotective response in rat brain MVECs in culture, with signaling via VEGFR1 and Akt but not VEGFR2 and p38. In the neuronal vasculature in vivo, VEGF-A is likely to be only part of a complex network of environmental cytoprotective factors (see below).

## Discussion

**Figure 7:**
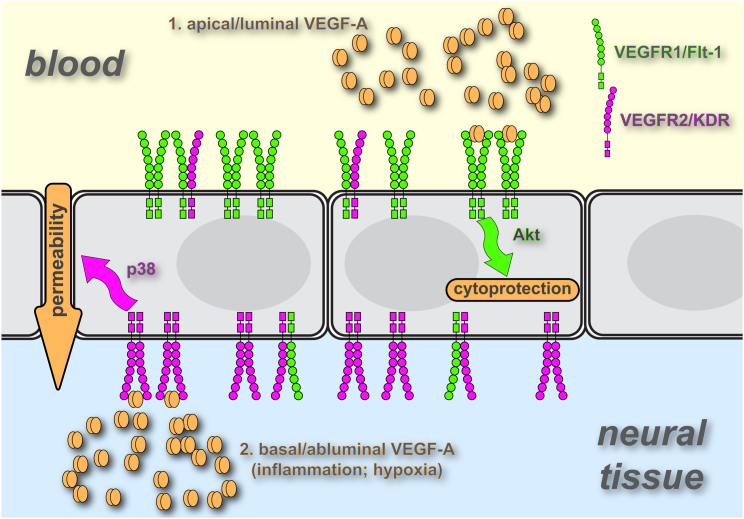
Model of VEGF Action on MVECs of Blood-Neural Barriers The majority of VEGFR1 and VEGFR2 is found on the apical (luminal) and basal (abluminal) side, respectively, of brain or retinal MVECs. Based on computer simulation (Mac Gabhann and Popel, 2007), such a receptor distribution is predicted to result primarily in VEGFR1 and VEGFR2 homodimers at the apical and basal surface, respectively. Residual apical VEGFR2 or basal VEGFR1 would not be found in homodimers but rather in heterodimers with the more abundant receptor. Signaling from VEGFR1, mainly triggered by circulating VEGF, activates a PI3K/Akt pathway, which plays a role in endothelial cytoprotection. Signaling from VEGFR2, triggered by tissue-borne VEGF, activates p38 and mediates paracellular permeability, hallmarked by macromolecular flux, electrical conductance, and a loss of Cldn5.

The vascular endothelium operates in a highly polarized environment, but to date there has been little exploration of polarized endothelial signaling. Here we demonstrate that luminal and abluminal EC surfaces of blood-neural barriers display different functionality during acute responses to VEGF-A as well as histamine, IGFBP3, and LPA. In particular, we found that cerebral and retinal vascular permeability was completely refractory to circulating VEGF-A. In contrast, the lung vasculature responded to circulating VEGF-A and VEGF-E with enhanced permeability and p38 activation. Because mesenteric and tracheal microvessels also induce permeability in response to luminal VEGF-A (Sun et al., 2012, Bates and Curry, 1996), our findings appear to have revealed a clear specialization of the blood-neural barrier endothelium. Importantly, our data are consistent with VEGF-A’s well-established role as a paracrine factor produced by hypoxic/ischemic tissue (see also Figure 7), for instance by astrocytes during central nervous system inflammatory disease (Argaw et al., 2012) and pathological retinal angiogenesis (Weidemann et al., 2010) or by Müller cells during diabetes-induced retinal ischemia (Wang et al., 2010). The sided responsiveness of these MVECs to VEGF-A was attributable to highly polarized expression of the receptor tyrosine kinases VEGFR1 and -R2 and distinct downstream activation of key signaling pathways.

VEGF receptor localization was polarized strongly but not in an absolute manner. Nevertheless, the permeability response and the signaling response of the microvascular endothelium was completely polarized. General computational modeling of VEGF receptor surface distribution (Mac Gabhann and Popel, 2007) suggests that the distribution pattern we observed in cerebral and retinal ECs would result in luminal VEGFR1 homodimers and some VEGFR1-R2 heterodimers but no VEGFR2 homodimers. In contrast, basal membranes would be rich in VEGFR2 homodimers (and heterodimers) but devoid of VEGFR1 homodimers (see also Figure 7). We speculate that this absence of VEGFR2 and VEGFR1 homodimers on the luminal and abluminal surface of neural ECs produces exclusivity with regard to location and receptor specificity of ligands. In particular, in this model, p38 and Akt activation would be exclusively associated with VEGFR2 and VEGFR1 homodimer signaling, respectively. Indeed, Cudmore et al. have recently shown that VEGFR2 yields different cellular responses depending on whether it is engaged in homo- or heterodimers (Cudmore et al., 2012). In addition, coreceptors such as neuropilin 1 may also play a role (Becker et al., 2005). Future experiments using heterodimeric ligands such as PlGF-1-VEGF-E (Cudmore et al., 2012) could establish the role of VEGFR1-R2 heterodimers in the differential signaling response of brain and retinal ECs.

In line with generally accepted views (Takahashi and Shibuya, 2005, Issbrücker et al., 2003), VEGFR2 and p38 activities were associated with the permeability-enhancing response of VEGF-A. In contrast, and judging by activation profiles and PI3K inhibition, we did not detect any involvement of Akt in VEGF-induced permeability in the brain. Indeed, conflicting results have been reported regarding a direct role of Akt in vascular permeability (Six et al., 2002, Chen et al., 2005), and its involvement has frequently been inferred by the need for activation of the Akt substrate eNOS during VEGF-mediated permeability (Takahashi and Shibuya, 2005). However, in cerebral MVECs, eNOS activation can also occur in an Akt-independent manner, in particular during inflammation (Martinelli et al., 2009). We did not find a role for VEGFR1 in mediating acute brain permeability either. This receptor has been proposed to mediate permeability in a more chronic and auxiliary way (Takahashi and Shibuya, 2005, Koch et al., 2011), which would not likely be captured in our model systems. In addition, a direct role of VEGFR1 in permeability may be restricted to nonneural vascular beds (Odorisio et al., 2002).

Instead, and in line with it activating Akt, a kinase regarded as synonymous to the cell-survival response (Datta et al., 1999), we found a clear role of VEGFR1 in cytoprotection. A cytoprotective role of VEGF-A for ECs is well documented (Gerber et al., 1999), with most mechanistic evidence derived from in vitro culture models. For instance, in umbilical vein ECs, the main VEGF receptor mediating survival appears to be VEGFR2 (Gerber et al., 1998, dela Paz et al., 2012), whereas in microvascular retinal or dermal ECs there is evidence that VEGFR1 is involved (Cai et al., 2003, Zhang et al., 2010), suggesting that receptor usage may differ between vascular beds. Whereas we were able to find clear experimental support for VEGFR2-mediated permeability in vitro and in vivo, the cytoprotective function of VEGFR1 has so far only been observed in cultured cerebral MVECs. Our failure to induce measurable apoptosis in retinal ECs in vivo through staurosporine and VEGF-A withdrawal suggests that additional growth (Walshe et al., 2009) or environmental factors such as flow (dela Paz et al., 2012, Dimmeler and Zeiher, 2000) contribute to EC survival, in particular in the brain and the eye (Ueno et al., 2008). Numerous experiments also show that the apoptotic response of ECs in vivo is relatively slow and only measurable after several days (Meeson et al., 1999, Sugimoto et al., 2003), and thus not within the time frame of our current experimental setup.

Overall, our findings are compatible with previous reports but clearly identify a high degree of specialization of VEGF signal transduction at blood-neural barriers. Specification of VEGF-A action occurs through many mechanisms, including the creation of functionally distinct protein isoforms, the presence of multiple surface, soluble, and coreceptors, and the sequestration of the ligand in the extracellular matrix (Koch et al., 2011). In addition, our data suggest that ECs are able to control the response to VEGF-A through compartmentalizing cellular signaling, and provide an intriguing explanation of how blood-neural barriers can be maintained despite constant exposure to circulating VEGF-A. Finally, the ability to map the vascular permeability response to a distinct topological signaling domain within the EC provides a new opportunity to specifically target pathological aspects of VEGF-A while preserving essential cardiovascular functions.

## Experimental Procedures

### Animals

Wistar and Lewis rats and C75BL/6J mice were purchased from Harlan Laboratories. All procedures were performed in accordance with UK and Ireland Animal Welfare Acts and with the Association for Research in Vision and Ophthalmology Statement for the Use of Animals in Ophthalmic and Vision Research and the Animal Welfare and the Ethical Review Bodies of the UCL Institute of Ophthalmology, King’s College London, and Trinity College Dublin.

### Recombinant VEGFs

Recombinant rat VEGF-A(165) was purchased from R&D Systems, VEGF-E was from Cell Sciences (or a kind gift from Kurt Ballmer-Hofer), and human PlGF-1 was from PeproTech.

### Magnetic Resonance Imaging

Vascular permeability in lungs, brain, and eyes was assessed in vivo via MRI (Campbell et al., 2012) using a dedicated small rodent 7 T MRI system (http://www.tcd.ie/neuroscience/infrastructure/neuroimaging/index.php#7tesla).

### In Vivo Permeability Measurements

The pial microvasculature of Wistar rats (age 25–30 days) was exposed and the permeability was measured as described previously (Easton and Fraser, 1994, Easton et al., 1997).

### MVEC Isolation

Microvessels were isolated from rat or mouse cortical gray matter or rat or porcine retinae by collagenase dispase digestion and BSA and Percoll density gradient centrifugation (Abbott et al., 1992). Purified vessels were seeded onto collagen IV/fibronectin-coated tissue-culture ware or Costar Transwells (3460) at high density (vessels from 6 rat brains or 12 retinae per 40 cm^2^ or 3 cm^2^, respectively). Cells were grown in EGM2-MV (Lonza) (with 5 μg/ml puromycin during the first 5 days; Perrière et al., 2007) for 2–3 weeks until their TEER plateaued at values above 200 Ω/cm^2^.

### Immunocytochemistry

MVECs were grown on collagen IV/fibronectin-coated tissue-culture ware or 12 mm Costar Transwell filters. Cells were fixed using 3.7% formaldehyde and extracted in acetone (−20°C). Alternatively, they were fixed and permeabilized simultaneously in 80% MeOH, 3.2% formaldehyde, 50 mM HEPES (pH 7.4) (Martins et al., 2013). Staining was performed as previously described (Turowski et al., 2004) using antibodies against von Willebrand factor (Dako), VE-cadherin (Martins et al., 2013), occludin and Cldn5 (Invitrogen), VEGFR1 (sc-31173; Santa Cruz Biotechnology), VEGFR2 (ab11939; Abcam), and P-glycoprotein (clone C219).

### Immunogold Electron Microscopy

Paraformaldehyde (PFA)-fixed brains from 10-month-old mice were isolated and 50 μm hippocampal slices were dissected out and postfixed for 2 hr in 4% PFA. MVECs on Transwell filter inserts were fixed in 4% PFA and 0.1% glutaraldehyde. Fixed samples were processed as previously described (Eden et al., 2010). Briefly, samples were embedded in 12% gelatin in 0.1 M phosphate buffer (pH 7.4). After infusion with 2.3 M sucrose at 4°C overnight, 80 nm sections were cut at −120°C using a cryoultramicrotome and collected in a 1:1 mixture of 2.3 M sucrose and 2% methylcellulose. The sections were then stained as previously described (Slot et al., 1991) using antibodies against VEGFR1 (sc-31173) (AF471; R&D Systems) and VEGFR2 (ab11939) (DC101). Samples were viewed with a JEOL 1010 transmission electron microscope, and images were gathered using a Gatan Orius SC100B charge-coupled device camera. Image manipulation was performed in Gatan Digital Micrograph and Adobe Photoshop. VEGFR distribution was determined by visual inspection of electron micrographs. Gold particles were considered luminal (apical) or abluminal (basal) when found within 20 nm of the respective plasma membrane (Ottersen, 1989).

### Transendothelial Flux

Fluorescein isothiocyanate (FITC) dextran (4 kDa) flux across MVECs was measured as previously described (Martins et al., 2013).

### Transendothelial Electrical Resistance

TEER was measured directly with chopstick electrodes (Turowski et al., 2004) and an EVOM voltohmmeter (World Precision Instruments). Alternatively, TEER was assessed by impedance spectroscopy using cells grown either on 12 mm Transwells and a cellZscope (nanoAnalytics) or on gold electrodes (eight-well 8W1E) and an ECIS (4,000 Hz) (Applied BioPhysics).

### Cytoprotection Assays

Caspase activity of MVECs grown in 96-well plates was measured using the Apo-ONE Homogeneous Caspase-3/7 assay kit (Promega).

### Cell-Surface Biotinylation

Apical and basal biotinylation was performed using a method adapted from Gottardi et al. (1995). Basal biotinylation was very weak, presumably because of reduced access through the filter and the basement membrane (Gottardi et al., 1995). Therefore, apical and basal domains were labeled simultaneously in the presence of EDTA. Biotinylated proteins were isolated on streptavidin beads and, following immunoblotting for VEGF receptors, apical and basal signals were quantified by densitometry and normalized against input signals. Basal signal was calculated from the combined apical and basal labeling minus the apical signal.

### Western Blots

For immunoblot analyses, samples were lysed in 50 mM Tris/Cl (pH 6.8), 2% SDS, 10% glycerol, 100 mM dithiothreitol (DTT), 100 nM calyculin A (50 μl/cm^2^ of cells), separated by SDS-PAGE, electrotransferred to nitrocellulose or polyvinylidene fluoride, and immunodecorated with phospho-specific and total antibodies as previously described (Martinelli et al., 2009). Akt (phospho-S473 and total), p38 (phospho-T180/Y182 and total), and anti-VEGFR2 antibodies were from Cell Signaling (55B11), and anti-VEGFR1 was from Abcam (Y103).

### VEGF Receptor Localization in Retinal and Pulmonary Vessels

Anti-VEGFR1 (sc-31173) and -VEGFR2 (DC101) antibodies (each at 6 mg/kg) were injected into the left ventricle of anesthetized p20 mice (C57 BL/6J). After 5 min, the animals were perfused with PBS followed by 4% PFA. Retinae were dissected out and processed for whole-mount staining (West et al., 2005). For abluminal detection, dissected unfixed retinae of PBS-perfused mice were overlaid with anti-VEGFR1 and -VEGFR2 antibodies (20 μg/ml) for 5 min, washed three times briefly with PBS, and then fixed in 4% PFA for 1 hr. All retinae were stained using anti-F4/80 (MCA497EL; AbD Serotec) and/or biotinylated isolectin B4 (B1205; Vector Labs) and secondary antibodies to detect bound VEGFR1 and -R2 antibodies.

Transversal (1 mm) sections of lungs from animals having undergone cardiac injections were analyzed in a similar manner. All samples were analyzed by confocal microscopy on a Zeiss LSM 700. Retinal flat mounts were imaged with a pinhole aperture allowing capture of the entire primary plexus depth in a single scan, whereas sections of lung were analyzed from projections of sequential image stacks.

### p38 and Akt Signaling In Vivo

For luminal stimulation, 120 μg/kg of VEGF-A, VEGF-E, or PlGF-1 or an equal volume (100 μl) of saline was injected into the tail vein of anesthetized P23 Wistar rats. For abluminal stimulation, cranial windows were surgically introduced and VEGFs were added at 100 ng/ml (final concentration) to the liquid pool superfusing the pial microvasculature. Five minutes after injection/VEGF application, animals were perfused and fixed and pial or lung sections were processed for immunohistochemistry (Powner et al., 2010). For Akt analysis, animals were injected with VEGFs in the tail vein as above or in the vitreous (using 100 ng of VEGF-A, VEGF-E, or PlGF-1 or an equal volume [5 μl] of saline). After ca. 25 min, retinae were isolated and lysed in 50 mM Tris/Cl (pH 6.8), 2% SDS, 10% glycerol, 100 mM DTT, 100 nM calyculin A and subjected to immunoblot analysis.

## Supplemental Information

Document S1. Supplemental Experimental Procedures, Figures S1–S4, and Table S1

Document S2. Article plus Supplemental Information
