# Supplementary material for: Differential Apicobasal VEGF Signaling at Vascular Blood-Neural Barriers
Source: Dev Cell. 2014 Sep 8;30(5):541–52. doi: 10.1016/j.devcel.2014.06.027 (PMC4160345; doi:10.1016/j.devcel.2014.06.027)
Supplement: Document S1. Supplemental Experimental Procedures, Figures S1–S4, and Table S1 [file mmc1.pdf]

Developmental Cell, Volume *30*

Supplemental Information

Differential Apicobasal VEGF Signaling at Vascular Blood-Neural Barriers

Natalie Hudson, Michael B. Powner, Mosharraf H. Sarker, Thomas Burgoyne, Matthew Campbell, Zoe K. Ockrim, Roberta Martinelli, Clare E. Futter, Maria B. Grant, Paul A. Fraser, David T. Shima, John Greenwood, and Patric Turowski

Figure S1

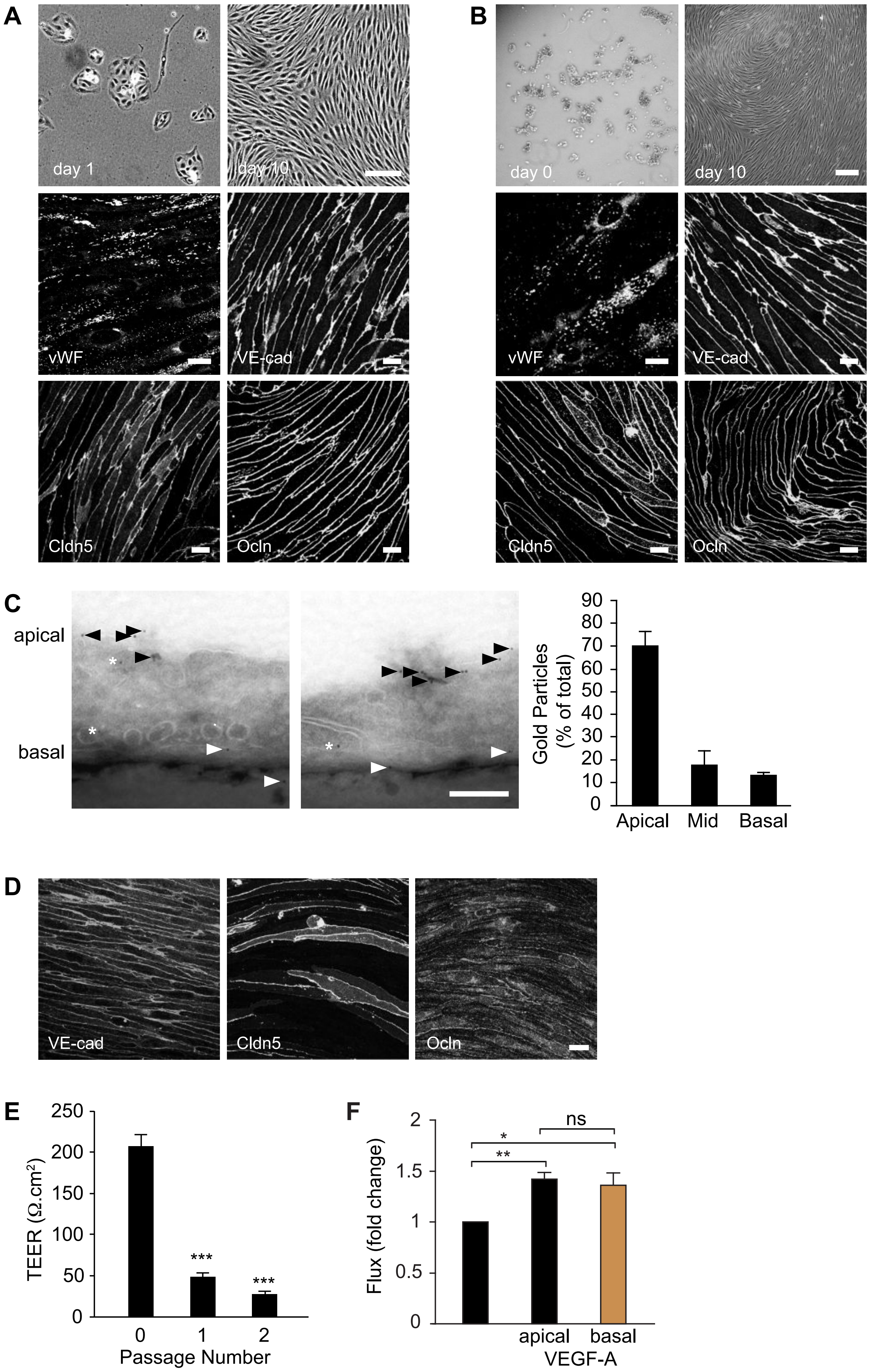
A
day 1
day 10
vWF
VE-cad
Cldn5
Ocln
B
day 0
day 10
vWF
VE-cad
Cldn5
Ocln
C
apical
basal
Gold Particles (% of total)
0
10
20
30
40
50
60
70
80
90
Apical
Mid
Basal
D
VE-cad
Cldn5
Ocln
E
TEER (Ω.cm²)
0
50
100
150
200
250
***
***
0
1
2
Passage Number
F
Flux (fold change)
0
0.5
1
1.5
2
ns
*
**
apical
basal
VEGF-A

Figure S2

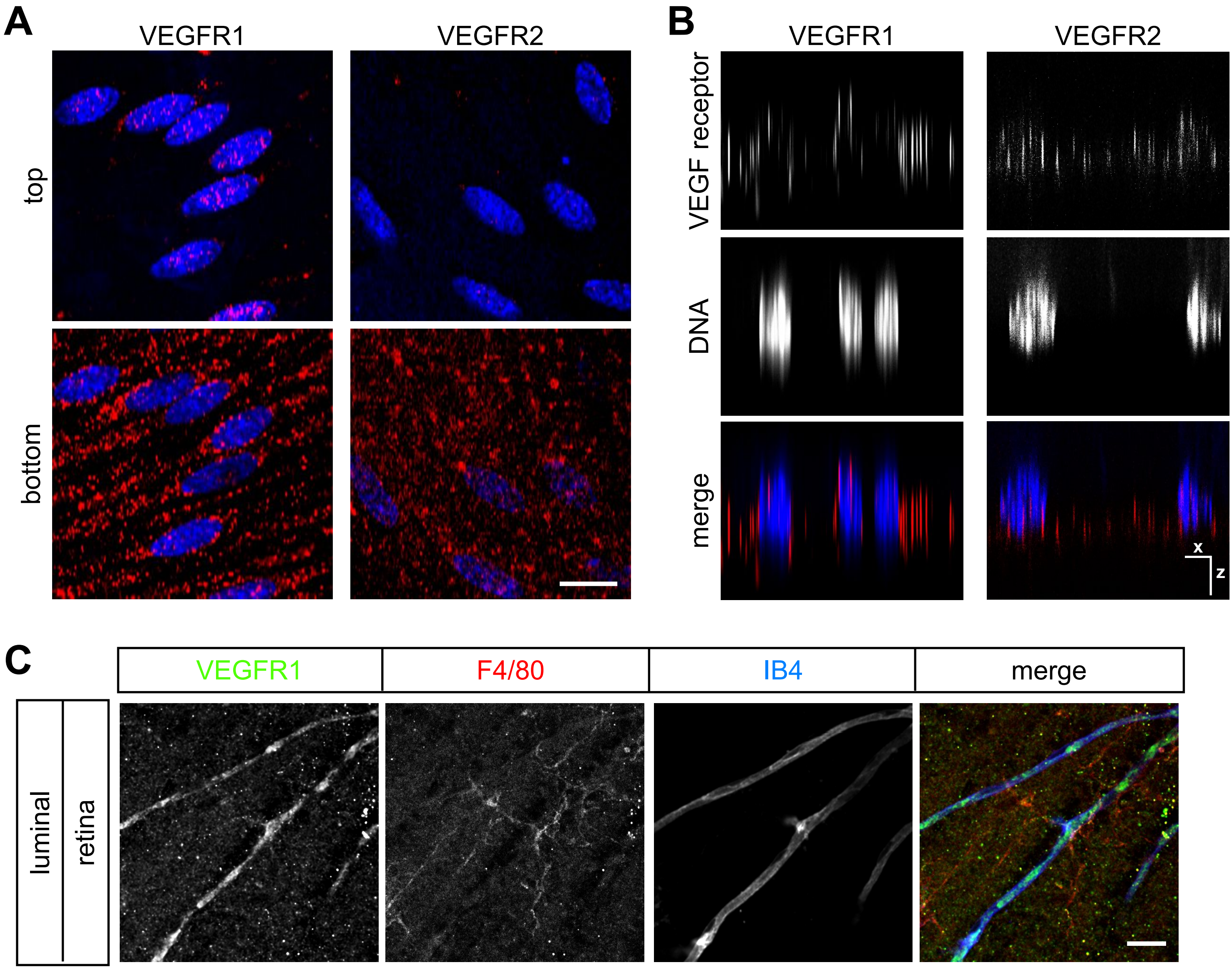
A
VEGFR1
VEGFR2
top
bottom
B
VEGFR1
VEGFR2
VEGF receptor
DNA
merge
x
z
C
VEGFR1
F4/80
IB4
merge
luminal
retina

Figure S3

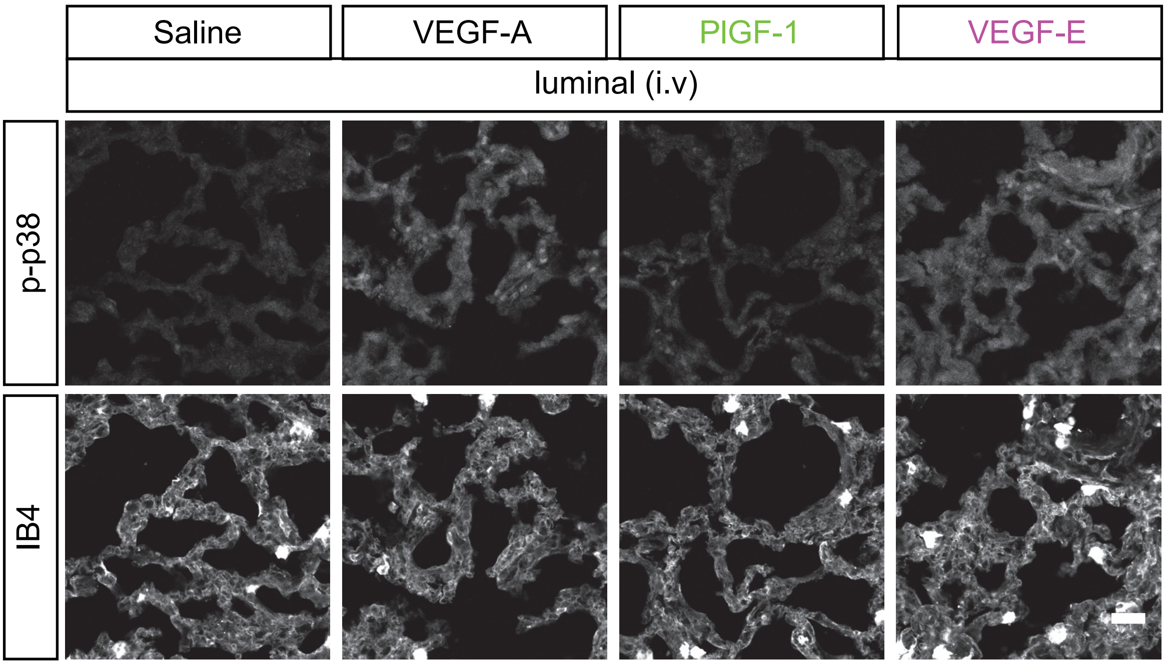

Figure S4

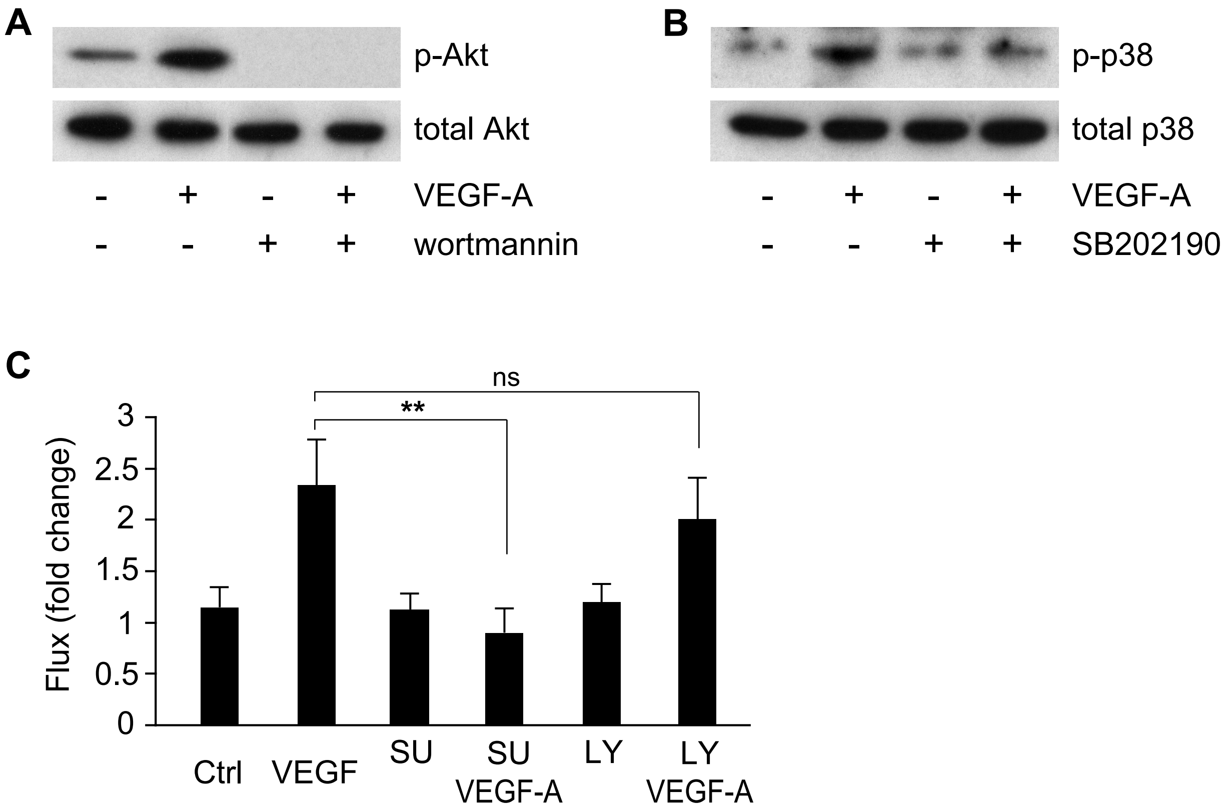

A
p-Akt
total Akt
- + - + VEGF-A
- - + + wortmannin
B
p-p38
total p38
- + - + VEGF-A
- - + + SB202190
C
ns
**
Flux (fold change)
3
2.5
2
1.5
1
0.5
0
Ctrl
VEGF
SU
SU VEGF-A
LY
LY VEGF-A

Supplemental Figure Legends

Figure S1, related to Figure 2: Characterization of primary rat brain and retinal MVECs.

(**A, B**) Microvessels were isolated from Lewis rat brains (A) or retinae (B) and seeded onto collagen IV/fibronectin coated dishes. Phase contrast microscopy showed that MVECs formed monolayers within 10 days. Scale bars, 100 µm. Cultures were also further characterized for the expression and distribution of von Willebrand Factor (vWF), VE-cad, Cldn5 and Occludin (Ocln) by indirect immunocytochemistry and confocal microscopy. Shown are full projections spanning the entirety of the cell monolayer. Scale bars, 10 µm. By all cytological criteria cerebral and retinal MVEC were indistinguishable and as expected junctional protein localization was restricted to intercellular contact areas. In agreement, barrier properties of cultures were very well developed. TEER reached a maximum of 345 ± 30 $\Omega.cm^2$ (n = 11 brain cultures) two weeks after vessel seeding. At the same time we measured very low macromolecular flux (1.883 ± 0.267 10^{-6} cm/s for 4 kDa FITC dextran), which so far has only been observed when brain MVECs are co-cultured with astrocytes (Perriere et al., 2007). (**C**) Rat brain EC monolayers were also analyzed for the distribution of p-glycoprotein by cryoimmuno-EM. Gold particles in the apical, internal or basal areas are indicated by black arrowheads, asterisks or white arrowheads, respectively. Scale bar, 200 nm. The panel on the right depicts quantification of gold particle distribution determined from three independent preparations and shows that p-glycoprotein, which is known to be enriched on the luminal side of BBB endothelium *in vivo* (Miller, 2010), localized predominantly to the apical face of our EC layers suggesting that significant apical-basal polarity was maintained. Importantly, barrier determinants of our MVEC preparations, in particular uninterrupted tight junction distribution and high TEER, were lost when cells were sub-cultured (see panels D-F). Consequently, all subsequent experiments were conducted on directly plated primary MVECs within two weeks of isolation when TEER was at least 200 $\Omega.cm^2$. (**D**) Microvessels were isolated from rat brains and grown on collagen IV/fibronectin coated dishes. Cells were passaged by trypsinization on three occasions (at 10-day intervals) and then allowed to reach confluence. Cells were then fixed, decorated using antibodies against VE-cad, Cldn5

and Ocln and analyzed by indirect immunofluorescence and confocal microscopy. Shown are full projections spanning the entirety of the cell monolayer. Scale bar, 10 µm. (**E**) TEER across confluent brain MVEC monolayers 14 days after passage 0, 1 and 2 (means ± SEM, n = 3). ***P < 0.001 (ANOVA and Dunnett's post hoc test). (**F**) The rat retinal MVEC cell line PT2 (details available on request) was passaged 9 times after immortalization and grown on permeable transwell inserts until confluent. Flux of 4 kDa FITC dextran was measured in the absence or presence of apical or basal VEGF-A (50 ng/ml). No predilection of apical or basal stimulation was observed. Similar results were obtained using the human or rat brain MVEC cell lines CMEC/D3 (Weksler et al., 2005) or GPNT (Romero et al., 2003) (data not shown). Shown are means ± SEM of three independent experiments. Statistical analysis was by Student's t-test with P > 0.05 (ns), *P < 0.05, **P < 0.01.

Figure S2, related to Figure 3: VEGF receptor distribution in MVECs.

(**A, B**) Confocal analysis of VEGF receptor distribution in primary brain MVECs. Rat cerebral MVECs were grown on permeable transwell inserts until they reached a TEER of greater than 200 $\Omega.cm^2$. Cells were fixed with a mixture of MeOH and formaldehyde and stained for VEGF receptors (red) using anti-R1 (sc-31173) or –R2 (ab11939). Distribution was analyzed by confocal microscopy. MVECs are extremely thin and apical or basal distribution cannot be determined in areas other than nuclei (DNA, blue). Shown in (A) are 400 nm sections through the very top or the bottom of cells, illustrating that in the nuclear area R1 is predominately apical whereas R2 is found underneath nuclei. Scale bar, 10 µm. Shown in (B) are representative z-section through nuclei (DNA, blue) illustrating significant VEGF receptor (red) staining for R1 on top of nuclei and for R2 below nuclei. Scale bars: x, 10 µm; z, 1 µm. **(C)** Circulating anti-VEGFR1 was not bound to monocytes or macrophages in the mouse retina. VEGFR1 (sc-31173) antibody was delivered into the vasculature of mice by cardiac injection and left to circulate for 5 min as described for Figure 3 C. After perfusion with PBS and fixation, retinae were dissected and permeabilized. Whole mount retinae were then processed with fluorescein-conjugated anti-goat antibodies to reveal bound anti-R1 antibodies and co-staining for the monocyte/macrophage marker F4/80 (red) and isolectin B4 (IB4, blue) and analyzed by confocal microcopy. Scale Bar, 20 µm.

Figure S3, related to Figure 4: p38 activation in lung microvessels *in vivo* in response to circulating VEGFs.

Intravenously injected VEGF-A and VEGF-E (at 120 μg/kg)(luminal) induce rapid and strong activation of p38 in the alveolar microvasculature of P23 rats. PlGF-1 injection only weakly activated p38. Lungs were fixed within 5 min of treatment and then stained for phosphorylated p38 (pT180/Y182). All sections were counterstained with the vessel marker isolectin B4 (IB4) and analyzed by confocal microcopy. Shown are projections spanning a thickness of ca. 8 μm. N.B.: lungs were from the same animals as the pial sections shown in Figure 4. Bar, 20 μm.

Figure S4, related to Figure 5: Inhibition of VEGF signaling in brain MVECs.

Rat cerebral MVECs were grown on permeable transwell inserts until they reached a TEER of greater than 200 $\Omega.cm^2$. They were then transferred into growth-factor reduced medium for 24 h before treated with 10 μM wortmannin (**A**) or SB202190 (**B**) or left untreated as indicated. Cells were then stimulated in the presence or absence of the inhibitor with 50 ng/ml VEGF-A from both the apical and basal side. After 60 min cells were lysed and analyzed by immunoblots. Shown are representative immunoblots immunodecorated for phospho-S473 and total Akt (A) or phospho-T180/Y182 and total p38 (B). (**C**) Mean (± SEM) 4 kDa FITC dextran flux changes in brain MVEC in response to 50 ng/ml VEGF-A following pre-treatment with 10 μM of the VEGFR2 selective inhibitor SU1498 (SU) or the PI3K inhibitor LY294002 (LY). $^{**}P < 0.01$ (Two-way ANOVA and Bonferroni post hoc).

Table S1, related to Figure 3: Summary of of immunogold EM quantification

	Mouse hippocampal microvessels		Rat brain microvascular endothelial cells		
**antibody**	**VEGFR1 (sc-31173)**	**VEGFR2 (DC101)**	**VEGFR1 (sc-31173)**	**VEGFR1 (AF471)**	**VEGFR2 (ab11939)**
**n (independent sections)**	5	4	1	3	3
**number of blood vessels**	9	8	n/a	n/a	n/a
**shown in Figure**	3B	3B	not shown	3A	3A
**apical membrane (total µm)**	108.497	129.542	11.511	32.556	112.575
**basal membrane (total µm)**	121.848	152.553	8.145	32.352	103.156
**apical gold (total)**	126	182	88	350	93
**basal gold (total)**	77	484	23	112	201
**apical gold/membrane(µm)**	1.161	1.405	7.645	10.751	0.826
**basal gold/membrane(µm)**	0.631	3.173	2.824	3.462	1.949
**apical gold (%)**	**64.8**	**30.7**	**73.0**	**75.6**	**29.8**
**basal gold (%)**	**35.2**	**69.3**	**27.0**	**24.4**	**70.2**

Mouse hippocampal microvessels or primary rat brain MVECs were processed for cryoimmunogold EM analysis using the indicated antibodies against VEGFR1 or –R2. Representative micrographs of these analyses are shown in Figure 3 A and B. Gold particles were then counted from n independent endothelial monolayer or blood vessel sections. Also shown are the total length of membrane analyzed. Only gold particles within 20 nm of the respective plasma membrane domain were included (Ottersen, 1989).

Full experimental procedures

Magnetic resonance imaging (MRI). Vascular permeability in lungs, brain and eyes was assessed *in vivo* via MRI (Campbell et al., 2012), using a dedicated small rodent 7 T MRI system (http://www.tcd.ie/ Neuroscience/infrastructure/neuroimaging/index.php#7tesla). The effects of VEGF-A or saline control were visualized using T1-weighted MR images (resolution, 0.156 mm×0.156 mm×5 mm; field of view: 20 mm×20 mm×17.9 mm3; matrix; 128×128×30; TR/TE: 500/2.7 ms; flip angle: 30° number of averages: 3; acquisition time: 2 min, 24 s; repetitions: 12) following administration of 100 µl of a 1 in 3 dilution of Gd-DTPA (Gadolinium diethylene-triamine penta-acetic acid), administered via the tail vein. VEGF-A was injected systemically (i.v. into the tail vein) at 3 µg/mouse. Assuming a blood volume of 2-3 ml in an adult mouse (and no rapid loss through tissue permeation or clearance) 3 µg of injected VEGF-A leads to a circulating concentration of 1-1.5 µg/ml. This is more than 1000-fold in excess of physiological plasma levels and also in the range of other reports studying elevated levels of VEGF (e.g. Sun et al., 2012). Alternatively, local injection of VEGF-A in the vitreous of the eye or into the cortex stereotaxically involved an injection of ca. 8 ng VEGF-A. The vitreous volume of a mouse eye is 5.3 µl. Therefore an assumed concentration of 1.5 µg/ml VEGF-A in the eye was comparable to systemic exposure. Quantitative assessment of Gd-DTPA leakage was made on 8-bit, grey scale images by subtracting the contrast intensity in selected regions of interest pre-injection of Gd-DTPA from that post injection of Gd-DTPA. Pseudocolored images using a 16-color look-up table (see Figure 1) were also generated for easier visualization of leakage areas.

***In vivo* permeability measurements in pial microvessels.** The method used in this study, and its theoretical basis, has been described previously (Easton and Fraser, 1994; Easton et al., 1997). The experiments were performed on Wistar rats (aged 25-30 days of either sex) within guidelines directed by The Animals (Scientific Procedures) Act 1986. The animals were anesthetized by an intraperitoneal injection of 60 mg/kg body weight sodium pentobarbital diluted in water (25% w/v), and maintained by supplemental injection of 10% of the original dose when necessary. At the end of the experiment animals were sacrificed by administering an overdose of the anesthetic. In these

weanling animals, which do have a fully developed blood-brain barrier (Butt et al., 1990), the dura and arachnoid was removed, thus exposing the microcirculation of the surface of the brain and leaving it free of any diffusion barrier towards the superfusing solution (artificial cerebrospinal fluid: 110.5 mM NaCl, 4.7 mM KCl, 2.5 mM $CaCl_2$, 1.1 mM KH_2PO_4, 1.25 mM $MgSO_4$, 25 mM $NaHCO_3$ and 15 mM N-[2-hydroxyethyl]piperazine-N,-[2-ethanesulphonic acid], pH 7.4, maintained at 37°C). A low molecular weight fluorescent dye, sulforhodamine B (580 Da), was introduced into the cerebral microcirculation via a bolus injection into the carotid artery, and viewed under 540/25 nm illumination. The fluorescent signal was captured through a microscope via an image-intensifier camera (Hamamatsu). Video microscopy (1 frame/2-5 s) allowed the recording of the time-dependent loss of dye in a single pial venular capillary trapped by a glass-occluding probe. Since the rate of fall of intravascular concentration of a small polar molecule is independent of the hydrostatic pressure, the concentration of dye early in the occlusion, before axial volume flux distorts the uniform axial concentration of dye in the region of measurement, will be $C_t = C_0 e^{-kt}$; where k = 4P/d, and where Ct and C0 are the dye concentrations at time t and time zero, respectively; P is the permeability and d the diameter of the vessel. All experiments were performed within 2 h from the start of surgery, when the preparation is stable without discernible change in baseline permeability (Sarker et al., 2000). Baseline permeability in all experiments was 0.2 -2 x 10^{-6} cm/s. For measurements the flow of superfusing solution was stopped so that a stable pool was formed between the water immersion objective and the brain surface. Dye was injected into the cerebral circulation while the occluding probe was placed over a selected vessel and lowered when the vessel had filled. The dye in the remaining circulation was washed out by the blood flow and did not recirculate. A baseline recording was started immediately for 20 to 60 s. Optionally, second baselines were obtained after 15-min incubation of the brain surface with 10 μM SB202190 or wortmannin. For abluminal application bradykinin, VEGF-A, PlGF-1 or VEGF-E were applied into the pool on the brain surface. The volume of the pool was known, which allowed careful control of final compound concentrations on the surface of the brain. Vasoactive compounds were washed away after ca. 60 s, at which point recordings were also stopped. Luminal bradykinin or VEGF exposure was carried out by injecting the stated concentration of VEGF into the internal carotid artery as a bolus mixed with the dye. Based on

fluorescent intensity measurements this replaced the blood in its entirety, thus the stated concentration was likely to be present in the occluded vessel without much dilution. Image analysis and densitometry was performed using ImageJ 1.45s (NIH).

MVEC isolation. Microvessels were isolated from rat or mouse cortical grey matter, or rat or porcine retinae by collagenase dispase digestion and BSA and percoll density gradient centrifugation (Abbott et al., 1992). Purified vessels were seeded onto collagen IV/fibronectin-coated tissue culture ware or Costar Transwells (3460) at high density (vessels from 6 rat brains or 12 retinae per 40 cm^2 or 3 cm^2, respectively). Cells were grown in EGM2-MV (Lonza) (with 5 μg/ml puromycin during the first 5 days (Perriere et al., 2007)) for 2 to 3 weeks until their TEER plateaued at values above 200 $\Omega.cm^2$. Experiments were performed in 1-week conditioned medium. For experiments analyzing intracellular signaling, cells were switched into EBM2, 0.5% FCS for 24 h prior to treatment and analysis. Low growth factor conditions did not significantly affect barrier properties as judged by TEER.

Immunocytochemistry. MVEC were grown on collagen IV/fibronectin-coated tissue culture ware or 12-mm Costar Transwell filters. Cells were fixed using 3.7% formaldehyde and extracted in acetone (-20°C). Alternatively, they were fixed and permeabilized simultaneously in 80% MeOH, 3.2% formaldehyde, 50 mM Hepes pH 7.4, a method often used for the fixation of C. *elegans* (Otori et al., 2006), which we found to preserve junction integrity and avoid osmotic shock of cell nuclei (Martins et al., 2013). Staining was performed as previously described (Turowski et al., 2004) using antibodies against von Willebrand Factor (Dako), VE-cad (Martins et al., 2013), occludin, Cldn5 (both Invitrogen), VEGFR1 (sc-31173, Santa Cruz Biotechnology), VEGFR2 (ab11939, Abcam) and P-glycoprotein (clone C219).

Immunogold electron microscopy. 10 month-old mice were perfused-fixed with 4% paraformaldehyde (PFA). Brains were isolated, 50 μm slices cut using a vibrating microtome and from these, hippocampi dissected out and then post-fixed for 2 h in 4% PFA. MVECs on Transwell filter inserts were fixed in 4% PFA and 0.1% glutaraldehyde. Fixed samples were processed as previously described (Eden et al., 2010). Briefly, samples were embedded in 12% gelatine in 0.1 M phosphate buffer at pH 7.4. After infusion with 2.3 M sucrose at 4°C overnight, 80-nm sections were cut at -120°C using a cryo-ultramicrotome and collected in 1:1 mixture of 2.3 M sucrose and 2%

methylcellulose. The sections were then stained as previously described (Slot et al., 1991) using antibodies against VEGFR1 (sc-31173)(AF471, R & D) and VEGFR2 (ab11939)(DC101). Samples were viewed on a Jeol 1010 TEM, and images were gathered using a Gatan OriusSC100B charge-coupled device camera. Further image manipulation was performed in Gatan Digital Micrograph and Adobe Photoshop. VEGFR distribution was determined by visual inspection of electron micrographs. Gold particles were considered to be localized luminal (apical) or abluminal (basal), when found within 20 nm of the respective plasma membrane (Ottersen, 1989).

Flux measurement. 4 kDa FITC dextran (1 mg/ml) was added to the apical side of confluent MVECs grown on 12-mm Transwell filters. Samples (50 µl) were removed from the basal chamber (and replaced by fresh medium) at 20-30 min intervals. In standard experiments baseline flux was recorded from untreated cells for 120 min, followed (optionally) by the baseline in the presence of small molecule inhibitors for at least another 60 min. Finally, vasoactive compounds were added and samples were removed for a period of at least 120 min. Fluorescence of samples was plotted against time and permeability changes were determined from linear slope changes before and after addition of the compound (see also Figure 2 A and B).

Transendothelial electrical resistance (TEER). TEER was measured directly with chopstick electrodes (Turowski et al., 2004) and an Evom voltohmmeter (World Precision Instruments). TEER values of empty collagen IV/fibronectin-coated transwells containing MVEC-conditioned medium were subtracted to determine monolayer TEER. Alternatively, TEER was assessed by impedance spectroscopy using cells grown either on 12-mm Transwells and a cellZscope (Nanoanalytics), or on gold electrodes (8-well 8W1E) and ECIS (4000 Hz; Applied Biophysics).

Cytoprotection assays. Caspase activity of MVEC grown in 96-well plates was measured using the Apo-One Homogenous Caspase-3/7 assay kit (Promega).

Cell surface biotinylation. Apical and basal biotinylation was performed using a method adapted from (Gottardi et al., 1995). Briefly, MVEC were grown on 24-mm Transwell filters to confluence and a TEER > 200 $\Omega.cm^2$. Apical biotinylation was performed by two 25-min incubations in 10 mM triethanolamine, 150 mM $NaCl_2$, 2 mM $CaCl_2$, pH 9.0 (BB) and 1.5 mg/ml sulfo-NHS-biotin (Pierce). Under the same conditions basal biotinylation was very weak, presumably because of reduced access

through the filter and the basement membrane (Gottardi et al., 1995). Therefore, apical and basal domains were labeled simultaneously in the presence of EDTA: MVEC monolayers were pre-treated with 1 mM EDTA for 5 min before labeling twice for 25 min in BB supplemented with 1.5 mg/ml sulfo-NHS-biotin (Pierce) and 1 mM EDTA. Cultures were then washed with PBS containing Ca^{2+}, Mg^{2+} and 100 mM glycine before lysis in RIPA buffer (50 mM Tris, pH 7.5, 150 mM NaCl, 1% NP40, 0.5% deoxycholate, and 0.1% SDS) supplemented with protease inhibitors (Roche). Clarified extracts (inputs) were incubated overnight with 500 µl of a 1:1 (vol/vol) slurry of streptavidin-agarose beads (Pierce). The beads were then washed three times with RIPA buffer, and bound proteins eluted by adding 50 mM Tris/Cl, pH 6.8, 2% SDS, 10% glycerol, 100 mM DTT and boiling. Following immunoblotting for VEGF receptors apical and basal signals were quantified by densitometry and normalized against input signals. Basal signal was calculated from the combined apical and basal labeling minus the apical signal.

Western blots. For immunoblot analyses samples were lysed in 50 mM Tris/Cl, pH 6.8, 2% SDS, 10% glycerol, 100 mM DTT, 100 nM calyculin A (50 $\mu l/cm^2$ of cells), separated by SDS-PAGE and electrotransfered to nitrocellulose or PVDF. For each series of samples, two identically loaded gels/immunoblots were run for immunodecoration with phospho-specific and total antibodies as previously described (Martinelli et al., 2009). Akt (phospho-S473 and total), p38 (phospho-T180/Y182 and total) and anti-VEGFR2 antibodies were from Cell Signaling (55B11), anti-VEGFR1 from Abcam (Y103).

VEGF receptor localization in retinal and pulmonary vessels. 300 µl of a mixture of anti-VEGFR1 (sc-31173) and -VEGFR2 (DC101) antibodies (each at 6 mg/kg) were slowly (at ca. 60 µl/min) injected into the left ventricle of anesthetized p20 mice (C57 BL/6J). The antibodies were left to circulate and 5 min after the injection animals were perfused with PBS, followed by 4% PFA. Retinae were dissected out, flattened and post-fixed with 4% PFA for 1 h (West et al., 2005). Retinae were washed briefly in PBS and incubated in block (1% FBS, 3% Triton X-100, 0.5% Tween 20, in 2 X PBS) for 2 h at room temperature. For abluminal detection, dissected unfixed retinae of PBS perfused mice were overlaid with anti-VEGFR1 and -VEGFR2 antibodies (20 µg/ml) for 5 min, washed three times briefly with PBS and then fixed in 4% PFA for 1 h. Next, retinae from cardiac injections and

overlays were incubated under gentle agitation with anti-F4/80 (MCA497EL, AbD Serotec) and/or biotinylated isolectin B4 (B1205, Vector Labs) at room temperature overnight. This was followed by incubation with appropriate secondary antibodies, washes and mounting in Mowiol.

Transversal (1-mm) sections of lungs were cut, post-fixed for 1 h in 4% PFA, equilibrated in 30% sucrose/PBS and embedded in OCT compound (Agar) (Powner et al., 2010). 20 µm cryo-sections were cut and collected on superfrost® plus slides (VWR). The slides were air dried for 2 h prior to incubation in section block (1% BSA, 0.5% Triton X-100, PBS) for 1 h at room temperature. The sections were then incubated in secondary antibody mix containing biotinylated isolectin B4 (B1205, Vector Labs). Following washes, sections were mounted in Mowiol.

All samples were analyzed by confocal microscopy on a Zeiss LSM700. Retinal flatmounts were imaged with a pinhole aperture allowing capture of the entire primary plexus depth in a single scan, while sections of lung were analyzed from projections of sequential image stacks.

p38 and Akt signaling *in vivo*. For luminal stimulation 120 µg/kg of VEGF-A, VEGF-E, PlGF-1 or equal volume (100 µl) of saline was injected into the tail vein of anesthetized P23 Wistar rats. For abluminal stimulation, cranial windows were surgically introduced (see above for full description) and VEGFs were added at 100 ng/ml (final concentration) to the liquid pool superfusing the pial microvasculature. 5 min after injection/application, animals were perfused with PBS followed by 4% PFA and the brain and lungs harvested. Approx. 1 mm-thick superficial brain surface sections containing the pial vessels were dissected out. Similarly, ca. 1-mm thick transverse cuts were made through the lungs. Tissues were subsequently post-fixed for 1 h in 4% PFA. Subsequently, tissues were processed for immunohistochemistry as described above using anti-phospho-p38 (T180/Y182) and isolectin B4. Six commercial antibodies against phospho-Akt were tested for staining of such tissue sections. However, none of them produced significant staining in any of the samples, even when additional controls such as insulin treatment/injections were included. Therefore *in vivo* Akt activation was analyzed by immunoblots of retinae: Luminal VEGF stimulation was as above, abluminal stimulation was performed by injecting 100 ng of VEGF-A, VEGF-E, PlGF-1 or equal volume (5 µl) of saline into the vitreous of P23 Wistar rats. After 25 min animals were sacrificed, injected eyes enucleated, the retina lysed in 50 mM Tris/Cl, pH 6.8, 2% SDS, 10% glycerol, 100 mM

DTT, 100 nM calyculin A. Retinal homogenates were syringed through a 26-gauge needle, boiled and analyzed on immunoblots.

Additional materials were rat recombinant VEGF-A(165) (R & D), VEGF-E (Cell Sciences or a kind gift from Prof. Kurt Ballmer-Hofer), PlGF-1 (Peprotech), IGF-nonbinding IGFBP3 (a gift from Dr. Robert Baxter), SB202190, wortmannin, LY294002, SU1498 (all Merck), bradykinin, LPA, thrombin, histamine, puromycin, staurosporine (all Sigma).

Supplemental references

Miller,D.S. (2010). Regulation of P-glycoprotein and other ABC drug transporters at the blood-brain barrier. Trends Pharmacol. Sci. *31*, 246-254.

Otori,M., Karashima,T., and Yamamoto,M. (2006). The Caenorhabditis elegans homologue of deleted in azoospermia is involved in the sperm/oocyte switch. Mol. Biol. Cell *17*, 3147-3155.

Romero,I.A., Radewicz,K., Jubin,E., Michel,C.C., Greenwood,J., Couraud,P.O., and Adamson,P. (2003). Changes in cytoskeletal and tight junctional proteins correlate with decreased permeability induced by dexamethasone in cultured rat brain endothelial cells. Neurosci. Lett. *344*, 112-116.

Sarker,M.H., Hu,D.E., and Fraser,P.A. (2000). Acute effects of bradykinin on cerebral microvascular permeability in the anaesthetized rat. J. Physiol *528 Pt 1*, 177-187.

Weksler,B.B., Subileau,E.A., Perriere,N., Charneau,P., Holloway,K., Leveque,M., Tricoire-Leignel,H., Nicotra,A., Bourdoulous,S., Turowski,P., Male,D.K., Roux,F., Greenwood,J., Romero,I.A., and Couraud,P.O. (2005). Blood-brain barrier-specific properties of a human adult brain endothelial cell line. FASEB J. *19*, 1872-1874.
