# Supplementary material for: Differential Apicobasal VEGF Signaling at Vascular Blood-Neural Barriers
Source: Dev Cell. 2014 Sep 8;30(5):541–52. doi: 10.1016/j.devcel.2014.06.027 (PMC4160345; doi:10.1016/j.devcel.2014.06.027)
Supplement: Document S2. Article plus Supplemental Information [file mmc2.pdf]

Developmental Cell
Article

Differential Apicobasal VEGF Signaling at Vascular Blood-Neural Barriers

Natalie Hudson,[1,6] Michael B. Powner,[1] Mosharraf H. Sarker,[1,3] Thomas Burgoyne,[1] Matthew Campbell,[4] Zoe K. Ockrim,[1,7] Roberta Martinelli,[1,8] Clare E. Futter,[1] Maria B. Grant,[5] Paul A. Fraser,[3] David T. Shima,[2] John Greenwood,[1] and Patric Turowski[1,*]
[1]Department of Cell Biology
[2]Department of Ocular Biology and Therapeutics
UCL Institute of Ophthalmology, University College London, 11-43 Bath Street, London EC1V 9EL, UK
[3]Cardiovascular Division, King's College London, 150 Stamford Street, London SE1 9NH, UK
[4]Neurovascular Genetics Laboratory, Smurfit Institute of Genetics, Lincoln Place Gate, Trinity College, Dublin 2, Ireland
[5]Eugene and Marilyn Glick Eye Institute, Department of Ophthalmology, Indiana University School of Medicine, 1160 West Michigan Street, Indianapolis, IN 46202, USA
[6]Present address: Neurovascular Genetics Laboratory, Smurfit Institute of Genetics, Lincoln Place Gate, Trinity College, Dublin 2, Ireland
[7]Present address: Moorfields Eye Hospital, City Road, London EC1V 9PD, UK
[8]Present address: Center for Vascular Biology Research, Beth Israel Deaconess Medical Center, Harvard Medical School, 330 Brookline Avenue, Boston, MA 02215, USA
*Correspondence: p.turowski@ucl.ac.uk
http://dx.doi.org/10.1016/j.devcel.2014.06.027
This is an open access article under the CC BY license (http://creativecommons.org/licenses/by/3.0/).

SUMMARY

The vascular endothelium operates in a highly polarized environment, but to date there has been little exploration of apicobasal polarization of its signaling. We show that VEGF-A, histamine, IGFBP3, and LPA trigger unequal endothelial responses when acting from the circulation or the parenchymal side at blood-neural barriers. For VEGF-A, highly polarized receptor distribution contributed to distinct signaling patterns: VEGFR2, which was found to be predominantly abluminal, mediated increased permeability via p38; in contrast, luminal VEGFR1 led to Akt activation and facilitated cytoprotection. Importantly, such differential apicobasal signaling and VEGFR distribution were found in the microvasculature of brain and retina but not lung, indicating that endothelial cells at blood-neural barriers possess specialized signaling compartments that assign different functions depending on whether an agonist is tissue or blood borne.

INTRODUCTION

Endothelial polarity is assumed to be mechanistically similar to that of epithelial cells, where it is well studied. Undoubtedly, the morphological and molecular organization of the vascular endothelial cells (ECs) at any of the diverse blood-tissue interphases must reflect the highly polarized environment in which they operate. For instance, vascular lumen formation is entirely dependent on segregation of apical and basal membrane compartments and subsequent EC polarization (Lizama and Zovein, 2013). A huge body of work has also demonstrated that at vascular blood-brain or blood-retinal barriers, neuronal homeostasis is maintained by highly polarized localization of transporters and channels, which regulate the directional movement of ions, drugs, metabolites, and toxins (Abbott et al., 2010). Thus, it is assumed that, in their naturally highly polarized environment, ECs have adopted differential apicobasal signaling. However, experimental proof of such polarized signaling processes is still missing.

Vascular endothelial growth factor (VEGF) family members, and in particular its most studied representative VEGF-A, are central to the creation of new blood vessels during normal development and growth but also in pathological situations such as tumorigenesis and ocular neovascular disease (Koch et al., 2011; Takahashi and Shibuya, 2005). Besides its angiogenic role, VEGF-A has additional effects on the vascular endothelium. It was originally discovered for its ability to trigger vascular permeability, and this permeability-enhancing property of VEGF-A is linked to interstitial fluid accumulation in tumors and psoriatic lesions, as well as tissue edema and concomitant vision loss in neovascular eye disease (Ferrara et al., 2007). VEGF-A also has beneficial roles in the systemic vasculature, regulating normal vascular tone and acting as a trophic factor for the vascular endothelium (Maharaj and D'Amore, 2007).

VEGF-A mediates its diverse functions through the use of multiple receptors, mainly the receptor tyrosine kinases VEGFR1 (Flt-1) and VEGFR2 (Flk-1/KDR) (Koch et al., 2011). The most common splice isoform VEGF-A(165), subject of the present study, additionally interacts with neuropilin 1 and various cell-surface heparan sulfate proteoglycans to modulate the intracellular response. VEGF-A belongs to a family of growth factors with five genes encoding VEGF-A, -B, -C, and -D and placental growth factors (PlGFs), each binding VEGFRs with different affinities. For instance, PlGF-1 and the viral gene product VEGF-E specifically bind and activate VEGFR1 and VEGFR2, respectively (Takahashi and Shibuya, 2005).

VEGF-A triggers a plethora of intracellular signaling steps in ECs (Koch et al., 2011). Standout mediators associated with the VEGF-A-induced endothelial permeability response are the

mitogen-activated protein kinases Erk and p38, phospholipases, protein kinase C, phosphatidylinositol 3-kinase (PI3K)-activated Akt, and endothelial nitric oxide synthase (eNOS) (Weis and Cheresh, 2005). However, given the complexity of VEGF-A signaling and the permeability response, as well as the morphological and cytological differences of various endothelia, it is not surprising that these signaling components remain under intense scrutiny, in particular with respect to their specific role in particular experimental models and their interaction with each other in controlling various effector mechanisms.

Because of VEGF-A's central role in the regulation of endothelial functions in health and disease, anti-VEGFs have become irreplaceable tools in treating pathological angiogenesis and permeability (Ferrara et al., 2007). However, systemic anti-VEGF-A therapies are associated with endothelial dysfunction leading to bleeding, inflammation, hypertension, proteinuria, and even lethality (Chen and Cleck, 2009). Consistent with these observations in patients, animal models demonstrate that VEGF-A is constitutively required to maintain vascular tone and vascular EC survival and that reductions in plasma VEGF-A levels cause vascular attrition and functional abnormalities (Sugimoto et al., 2003).

We therefore hypothesized that circulating and tissue-produced VEGF-A induce distinct responses in ECs. In light of the central role of VEGF-A as a permeability-inducing factor in brain (Merrill and Oldfield, 2005; Argaw et al., 2012) and eye pathologies (Miller et al., 2013), our study focused on vascular ECs at blood-neural barriers and on acute endothelial permeability (as opposed to chronic interstitial fluid accumulation during inflammation, cancer, and wound healing, which may involve cells other than ECs) (Nagy et al., 2008). Our study is also restricted to the in vitro and in vivo analysis of the microvasculature as opposed to the macrovasculature, because this is where physiological and pathological vascular permeability occurs. Here we report that VEGF-A induces unequal responses depending on whether it acts on the luminal or abluminal side of neural microvascular ECs (MVECs). Mechanistically, this differential EC response is predicated on polarized VEGFR expression and distinct downstream signaling.

RESULTS

Using contrast-enhanced magnetic resonance imaging (MRI) in the mouse, we determined tissue extravasation of intravenous (i.v.) injected gadolinium (gadolinium diethylenetriamine pentaacetic acid [Gd-DTPA], 742 Da). Basal leakage of Gd-DTPA was significantly higher in peripheral tissues such as the lung than the brain or the eye, reflecting the significant difference in baseline permeability between neural and nonneural vascular beds. Intravenous injection of VEGF-A (3 μg/mouse) significantly enhanced leakage of Gd-DTPA into lungs within 10 min (Figure 1A), in agreement with previous reports of VEGF-A-induced permeability in nonneural vasculature, such as that of the trachea or the mesentery (Sun et al., 2012; Bates and Curry, 1996). Significantly, no leakage was observed in the brain or the eye over a 30 min observation period. However, when VEGF-A was directly injected into the brain cortex or the vitreous of the eye, rapid and significant accumulation of Gd-DTPA in the vicinity of the injection site was observed (Figures 1B and 1C), indicating that only tissue-borne but not circulating VEGF-A induced vascular hyperpermeability in the neural tissues.

This was further studied in single pial microvessels, which exhibit barrier properties closely resembling those found at the intact blood-brain barrier (BBB) and allow permeability measurements that are tightly controlled with respect to kinetics and dose (Butt et al., 1990; Easton et al., 1997). Increasing VEGF-A levels up to 2 μg/ml within the lumen of tight pial microvessels did not affect baseline permeability (Figures 1D and 1F), even when bradykinin triggered strong vascular permeability (Figures 1E and 1F). In contrast, exposure to VEGF-A from the tissue side led to a rapid increase of permeability (Figures 1G and 1H), which was maximal at a concentration of ca. 50 ng/ml. Taken together, these data demonstrated that cerebral microvascular permeability is strongly induced by VEGF-A, albeit only when presented to the abluminal face of microvessels.

The microvasculature in the brain and retina constitutes a network of ECs tightly interacting with mural cells such as pericytes and glial cells including astrocytes and Müller cells (Abbott et al., 2010). Because microvascular permeability operates primarily on the level of endothelium, we tested whether the ECs themselves responded to VEGF in a polarized fashion. For this, we used highly purified nonpassaged, primary MVECs isolated from brain or retina, which were characterized by preservation of interendothelial junctions, apicobasal polarity, and very good in vitro barrier properties (Figure S1 available online). Macromolecular apical-to-basal flux was enhanced nearly 2-fold when 50 ng/ml VEGF-A was added to the basal side of rat brain MVEC monolayers, whereas doses of up to 1 μg/ml applied to the apical side did not change flux (Figures 2A–2D). A similar, polarized response to VEGF-A was also observed in cultures of murine brain (data not shown) and rat or porcine retinal MVECs (Figures 2E and 2F), suggesting that functional polarity operates at all vascular blood-brain and -retinal barriers and across species. Importantly, both barrier properties and the sided response to VEGF-A were lost following repeated subculturing of MVECs (Figures S1D–S1F), offering a plausible explanation as to why this phenomenon has not yet been universally observed. VEGF-A also induced changes in transendothelial electrical resistance (TEER) and significant reduction in junctional claudin 5 (Cldn5) (Figures 2G and 2H), a reliable molecular indicator of MVEC barrier and BBB integrity (Nitta et al., 2003), indicating that paracellular rather than transcellular permeability was operational (Steed et al., 2010). VEGF-A provoked flux and TEER changes within minutes (Figures 2B and 2I), thus resembling the acute response observed in vivo. A sided permeability response similar to that with VEGF-A was also observed with histamine and insulin-like growth factor-binding protein 3 (IGFBP3) (Figure 2J), whereas opposite responsiveness was observed with lysophosphatidic acid (LPA). Thrombin and bradykinin induced significant flux in brain MVECs but without predilection for either endothelial face. Significantly, the sided response to histamine and LPA was consistent with previous results from pial microvessels in vivo (Sarker et al., 1998, 2010).

One explanation for the polarized responses to VEGF-A is that distinct signaling pathways, and in particular different VEGF-A receptors, operate on the apical and basal sides of brain or retinal MVECs. To test this hypothesis, we studied the distribution of VEGFR1 (Flt-1) and VEGFR2 (KDR/Flk-1) (Koch et al.,

CellPress

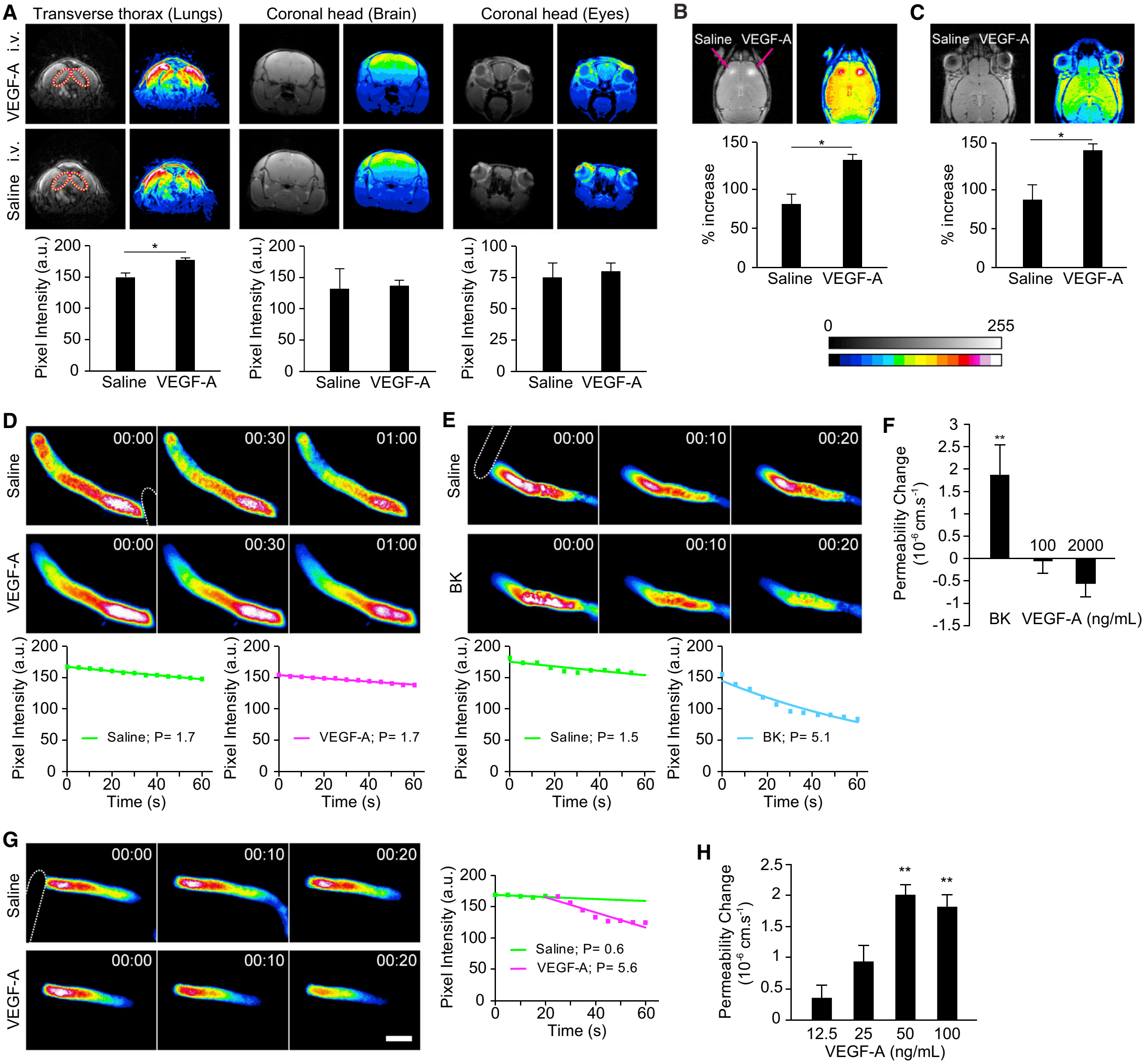

Figure 1. Abluminal but Not Luminal VEGF-A Induces Permeability In Vivo

(A–C) T-1-weighted MRI in mice showed increased extravasation of Gd-DTPA (742 Da) in the lungs (circled) but not the brain or eyes in response to i.v. VEGF-A (3 μg/animal) (A). In contrast, direct injection of VEGF-A (ca. 8 ng) into the brain cortex (B; arrows indicate injection sites) or the vitreous of the eye (C) led to increased extravasation. Shown are representative contrast-enhanced and 16-color pseudocolored images taken 8 min after VEGF-A injection, with densitometric quantification of each experimental group shown beneath. a.u., arbitrary units.

(D and E) Time-dependent recording of sulforhodamine B (580 Da) loss from single occluded rat pial microvessels in vivo showed no change of permeability in response to an intracarotid (i.e., luminal) bolus injection of VEGF-A (100 ng/ml) (D). In contrast, luminal bradykinin (BK) at 10 μM induced a rapid loss of dye (E). Shown are micrographs of pseudocolored microvessels at indicated times (min:s) after either saline or VEGF-A or bradykinin injection. The dotted outline in the first image in each series indicates the position of the occluding probe. Densitometric fluorescence intensities were plotted against time, and permeability values P (10^{-6} cm/s) were calculated by fitting data to the equation $C_t = C_0\,e^{-kt}$, where k = 4P/d and d is the diameter of the vessel.

(F) Mean permeability changes determined as described in (D) and (E) in response to either 10 μM bradykinin or 100 or 2,000 ng/ml VEGF-A.

(G) Application of VEGF-A (100 ng/ml) to the abluminal, extravascular space of single pial microvessels in vivo produced a strong increase of permeability to sulforhodamine B (580 Da). The scale bar represents 50 μm.

(H) Mean permeability changes in response to increasing concentration of VEGF-A.

Data are means ± SEM of at least three independent experiments. **$p < 0.01$, ***$p < 0.001$ [Student’s t test (A–C) and ANOVA and Dunnett’s post hoc test (F and H)].

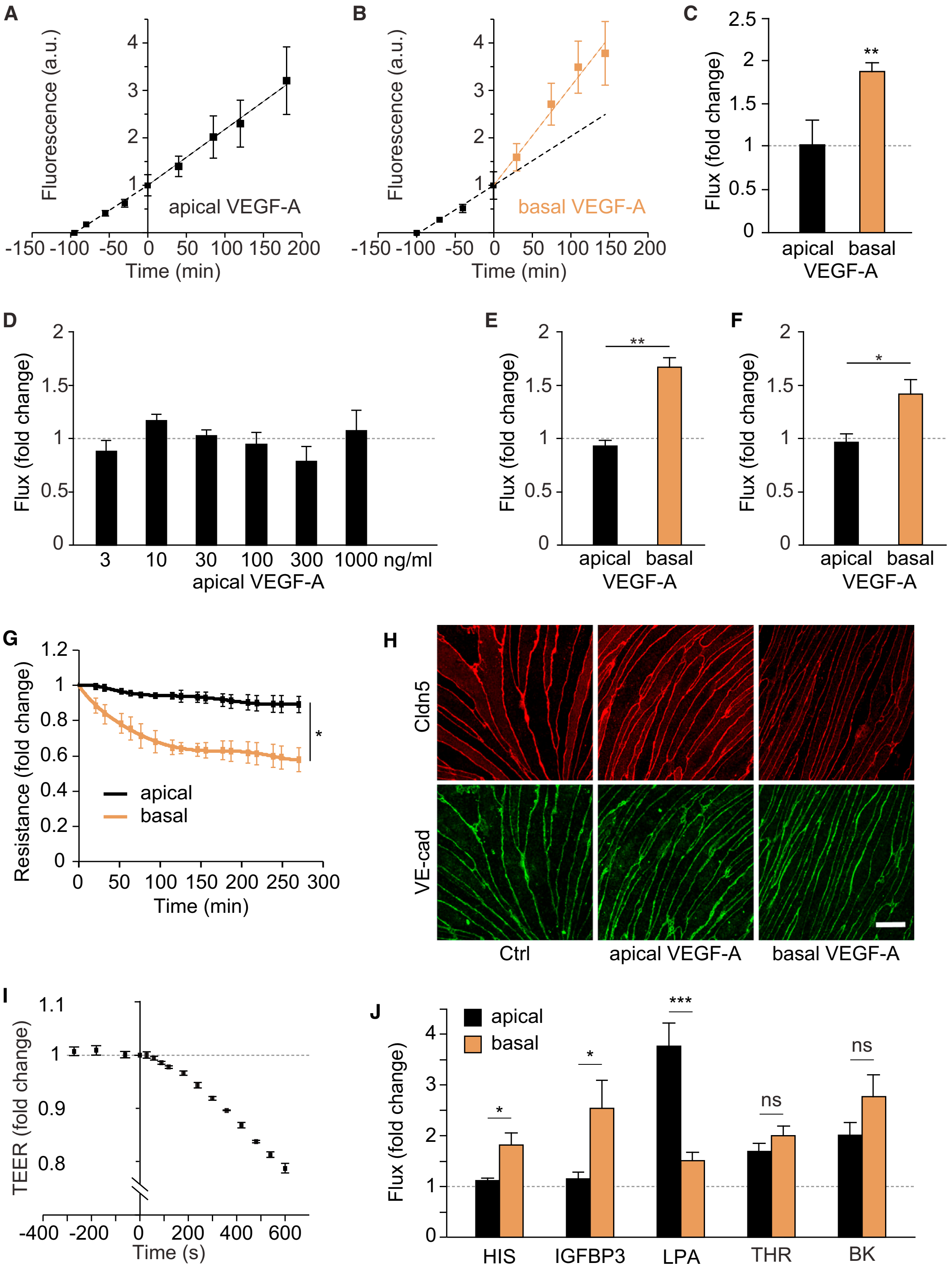

Figure 2. Basal, but Not Apical, VEGF-A Induces Permeability in Cultured Cerebral or Retinal MVECs

(A–C) Rat cerebral MVECs were grown on permeable Transwell inserts until they reached a TEER of greater than 200 Ω/cm^2. FITC dextran (4 kDa) was added to the apical chamber and flux was measured as the time-dependent accumulation of fluorescence in the basal chamber. VEGF-A (50 ng/ml) addition to the apical side of the cells (at time 0) did not change flux rates (A). In contrast, a rapid change in flux was observed when it was added to the basal side of the cells (B). Dotted lines are linear regressions of data points before and after the addition of VEGF-A. Mean fold changes of flux following apical or basal application are shown in (C).

(D) Flux was measured as in (A)–(C) in the presence of apical VEGF-A at the indicated concentrations, none of which led to significant flux changes.

(E and F) Basal but not apical VEGF-A (50 ng/ml) also induced significant 4 kDa FITC dextran flux changes in postconfluent rat (E) or porcine (F) retinal MVECs.

(legend continued on next page)

2011). Cryoimmunogold electron microscopy (EM) showed that ca. 75% of VEGFR1 was found apically in rat brain MVECs, whereas ca. 70% of VEGFR2 was found at basal membranes (Figure 3A; Table S1). Analysis by domain-specific biotinylation (Figure 3C) or analysis by confocal microscopy (Figures S2A and S2B) confirmed this highly polarized distribution pattern. Importantly, distribution of VEGFR1 and VEGFR2 was also predominantly luminal and abluminal, respectively, in the microvascular endothelium of the mouse hippocampus (Figure 3B; Table S1). VEGF receptor distribution was also interrogated in vivo: the luminal side of the mouse vasculature was exposed for 5 min to anti-VEGFR1 or -R2 antibodies by cardiac injection. Following perfusion, VEGFR antibody binding was studied by fluorescent secondary antibody labeling of retinae and lungs (Figure 3D; Figure S2C). VEGFR1 antibodies bound to retinal and lung microvessels, whereas VEGFR2 antibodies strongly labeled lung but not retinal microvessels. In contrast, when the same antibodies were applied abluminally for 5 min to unfixed and nonpermeabilized retinae, VEGFR2 but not -R1 was detectable in retinal vessels. In addition, both antibodies strongly reacted with other cells in the retinal ganglion cell layer, consistent with the reported expression of VEGF receptors in ganglion and Müller cells (Saint-Geniez et al., 2008; Foxton et al., 2013). Taken together, the majority of VEGFR1 and -R2 was found to be inversely located to the luminal and abluminal sides, respectively, of MVECs in the brain and the retina but not the lung.

These data strongly suggested that different downstream signals operate in response to apical or basal VEGF. Indeed, we found that the time-dependent activation of p38 and Akt was significantly different following apical or basal exposure of brain MVECs to VEGF-A. Phosphorylation of p38, increasing over time, was observed in response to basal but not apical VEGF-A (Figure 4A). In contrast, Akt activation was only seen following apical exposure to VEGF-A. Apical but not basal exposure to PlGF-1, which selectively binds to VEGFR1 (Takahashi and Shibuya, 2005), also induced Akt phosphorylation, with a peak at ca. 30 min (Figure 4B). In contrast, stimulation with the VEGFR2-selective ligand VEGF-E (Takahashi and Shibuya, 2005) did not lead to Akt activation from any side of the ECs. Instead, strong phosphorylation of p38 was observed within 5 min of basal but not apical exposure.

The hallmarks of this differential p38 and Akt activation pattern could be recapitulated by VEGF treatment in vivo. In rats, intravenous injection of neither VEGF-A, PlGF-1, nor VEGF-E (120 μg/kg) led to any activation of p38 in pial microvessels (Figure 4C). Nevertheless, in alveolar lung vessels of the same animals, p38 was strongly and rapidly activated in response to i.v. VEGF-A or VEGF-E but not PlGF-1 (Figure S3). In pial microvessels, VEGF-A and VEGF-E led to rapid p38 activation, but only when growth factors were administered from the tissue side through a cranial window (Figure 4C). We also attempted to study Akt activation in a similar manner. However, none of six different commercial anti-phospho-Akt antibodies produced significantly altered staining patterns in brain, lung, or retinal tissues of rats treated with time courses of luminal or abluminal VEGFs or indeed insulin, suggesting that reliable phospho-Akt detection was not possible by immunohistochemistry (data not shown). We therefore opted to study Akt activation in whole-cell lysates of retinal tissue. Intravenous injection of VEGF-A or PlGF-1 but not VEGF-E led to strong Akt activation after 25 min (Figure 4D). Some weak activation of p38 was also observed under these conditions. Direct injection of VEGFs into the retina (i.e., abluminal application) did not lead to any significant Akt phosphorylation. Taken together, these data demonstrated that in cerebral or retinal MVECs, rapid p38 activation occurred downstream of abluminal VEGFR2. In contrast, Akt activation occurred more slowly and downstream of luminal VEGFR1.

This polarized signaling pattern was not only in full agreement with the VEGFR1 and -R2 localization described in Figure 3 but also with the endothelial permeability response. Enhanced macromolecular flux across brain MVECs was seen following basal (but not apical) VEGF-E stimulation, whereas PlGF-1 had no effect on barrier function, neither in vitro nor in vivo (Figures 5A and 5B). Furthermore, we found that SB202190, but not wortmannin or LY294002, which inhibited VEGF-induced p38 or Akt activation, respectively (Figures S4A and S4B), abrogated VEGF-induced hyperpermeability in vitro and in vivo (Figures 5C and 5D; Figure S4C). Taken together, this clearly indicated that the permeability response to VEGF at blood-neural barriers is mediated by abluminal VEGFR2. In agreement, the VEGFR2 inhibitor SU1498 completely abolished VEGF-A-induced flux in brain MVECs (Figure S4C).

Abundant data suggest that systemic (hence, luminal) VEGF-A is a trophic factor for the vasculature (Maharaj and D'Amore, 2007; Gerber et al., 1998). In light of the specific activation of Akt following luminal VEGFR1 stimulation, we investigated a potential role in endothelial cytoprotection. Rat brain MVECs were stimulated with the microbial alkaloid staurosporine, widely used to induce apoptosis in most cell types, including ECs (Kabir et al., 2002). Apoptosis, measured as an increase in caspase 3/7 activity, was significantly reduced by apical VEGF-A or PlGF-1 but not VEGF-E (Figure 6A). This VEGF-mediated cytoprotection was sensitive to wortmannin but not to SB202190 (Figure 6B). To this end, a potential role of VEGF in cytoprotection of retinal ECs could not be interrogated in vivo, because staurosporine was ineffective in inducing EC death. Staurosporine was applied

(G) Postconfluent rat cerebral MVECs grown on permeable Transwell inserts were subjected either apically or basally to 50 ng/ml VEGF-A, and time-dependent changes in electrical resistance were monitored by impedance spectroscopy.

(H) Changes in the distribution of Cldn5 and VE-cadherin in response to either apical or basal VEGF-A (50 ng/ml, 1 hr) were analyzed by confocal microscopy in postconfluent rat cerebral MVECs. Shown are representative projections spanning the entire thickness of the monolayers. The scale bar represents 10 μm.

(I) As in (G), except that direct TEER changes were measured every ca. 30 s using chopstick electrodes. VEGF-A (50 ng/ml) was added to the basal side at time 0.

(J) Transendothelial flux was measured in postconfluent primary rat brain MVECs in response to apical or basal histamine (100 μM; HIS), IGFBP3 (50 ng/ml), LPA (10 μM), thrombin (1 U/ml; THR), or bradykinin (10 μM; BK).

Data are means ± SEM of at least three independent experiments. *$p < 0.05$, **$p < 0.01$, ***$p < 0.001$; ns, not significant [Student's t test (C, E, F, and J), ANOVA (D), or two-factor analysis of variance (location of VEGF application by time) with one repeated measure (time) (G)]. Note that in (J), only p values of apical versus basal treatments are shown.

See also Figure S1.

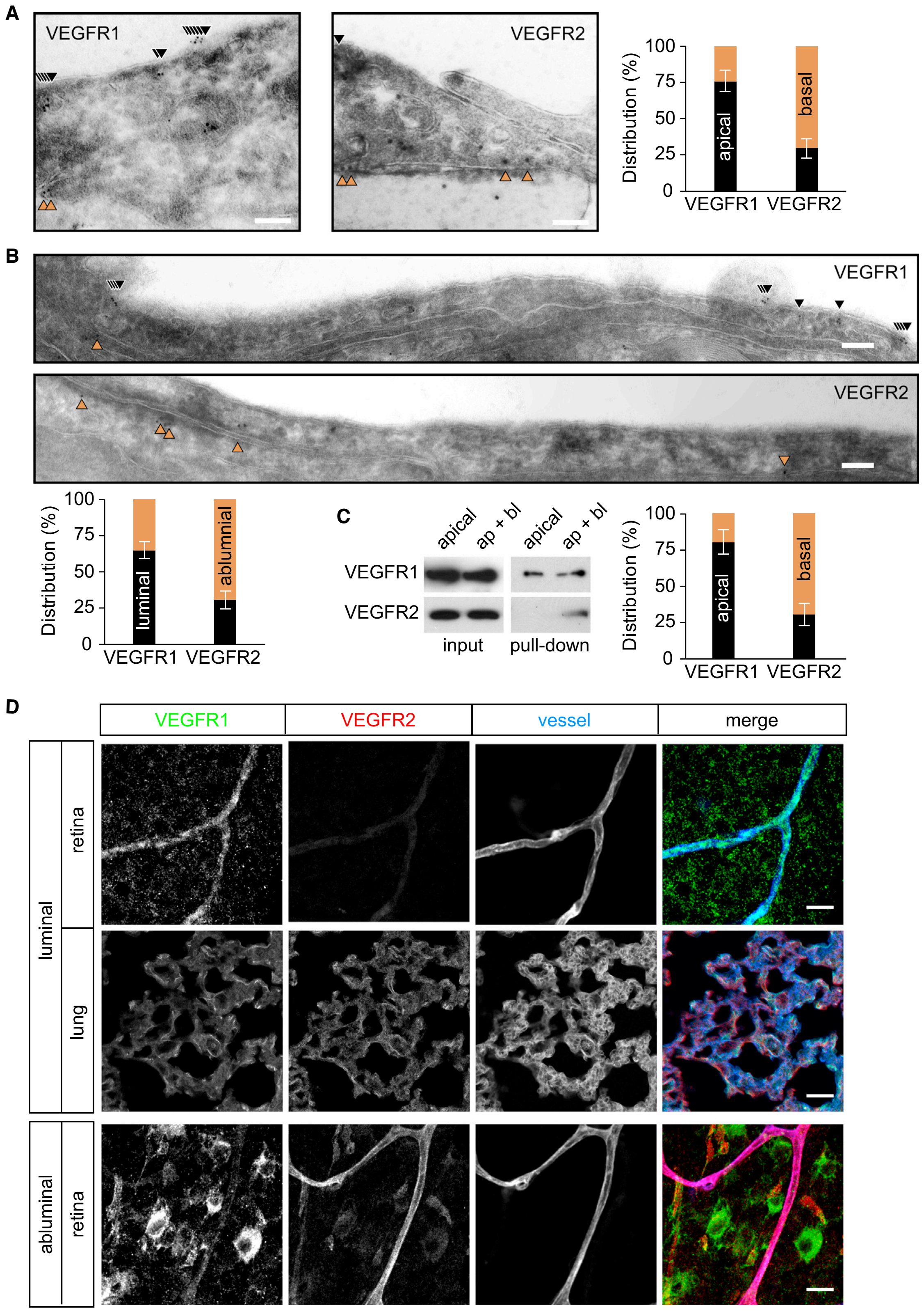

Figure 3. Differential VEGF Receptor Localization in Microvascular ECs in Brain and Retina

(A and B) Cryoimmunogold EM analysis of VEGFR1 and -R2 in primary rat brain MVECs (A) or mouse hippocampal microvessels (B) showed predominant apical/luminal (black) and basal/abluminal (brown) localization, respectively. Mean distribution of each VEGF receptor (±SEM) was determined by quantifying gold

(legend continued on next page)

to mouse or rat retinae both intravitreally and through the carotid artery at up to 40 μM for up to 12 hr. This treatment induced severe retinal ganglion cell death but did not affect ECs at all (data not shown). Even when additionally circulating VEGF-A was reduced by coadministration of anti-VEGF antibodies, we could not measure any annexin V binding or activated caspase 3 staining in retinal ECs (data not shown). Thus, VEGF mediates a cytoprotective response in rat brain MVECs in culture, with signaling via VEGFR1 and Akt but not VEGFR2 and p38. In the neuronal vasculature in vivo, VEGF-A is likely to be only part of a complex network of environmental cytoprotective factors (see below).

DISCUSSION

The vascular endothelium operates in a highly polarized environment, but to date there has been little exploration of polarized endothelial signaling. Here we demonstrate that luminal and abluminal EC surfaces of blood-neural barriers display different functionality during acute responses to VEGF-A as well as histamine, IGFBP3, and LPA. In particular, we found that cerebral and retinal vascular permeability was completely refractory to circulating VEGF-A. In contrast, the lung vasculature responded to circulating VEGF-A and VEGF-E with enhanced permeability and p38 activation. Because mesenteric and tracheal microvessels also induce permeability in response to luminal VEGF-A (Sun et al., 2012; Bates and Curry, 1996), our findings appear to have revealed a clear specialization of the blood-neural barrier endothelium. Importantly, our data are consistent with VEGF-A's well-established role as a paracrine factor produced by hypoxic/ischemic tissue (see also Figure 7), for instance by astrocytes during central nervous system inflammatory disease (Argaw et al., 2012) and pathological retinal angiogenesis (Weidemann et al., 2010) or by Müller cells during diabetes-induced retinal ischemia (Wang et al., 2010). The sided responsiveness of these MVECs to VEGF-A was attributable to highly polarized expression of the receptor tyrosine kinases VEGFR1 and -R2 and distinct downstream activation of key signaling pathways.

VEGF receptor localization was polarized strongly but not in an absolute manner. Nevertheless, the permeability response and the signaling response of the microvascular endothelium was completely polarized. General computational modeling of VEGF receptor surface distribution (Mac Gabhann and Popel, 2007) suggests that the distribution pattern we observed in cerebral and retinal ECs would result in luminal VEGFR1 homodimers and some VEGFR1-R2 heterodimers but no VEGFR2 homodimers. In contrast, basal membranes would be rich in VEGFR2 homodimers (and heterodimers) but devoid of VEGFR1 homodimers (see also Figure 7). We speculate that this absence of VEGFR2 and VEGFR1 homodimers on the luminal and abluminal surface of neural ECs produces exclusivity with regard to location and receptor specificity of ligands. In particular, in this model, p38 and Akt activation would be exclusively associated with VEGFR2 and VEGFR1 homodimer signaling, respectively. Indeed, Cudmore et al. have recently shown that VEGFR2 yields different cellular responses depending on whether it is engaged in homo- or heterodimers (Cudmore et al., 2012). In addition, coreceptors such as neuropilin 1 may also play a role (Becker et al., 2005). Future experiments using heterodimeric ligands such as PlGF-1-VEGF-E (Cudmore et al., 2012) could establish the role of VEGFR1-R2 heterodimers in the differential signaling response of brain and retinal ECs.

In line with generally accepted views (Takahashi and Shibuya, 2005; Issbrücker et al., 2003), VEGFR2 and p38 activities were associated with the permeability-enhancing response of VEGF-A. In contrast, and judging by activation profiles and PI3K inhibition, we did not detect any involvement of Akt in VEGF-induced permeability in the brain. Indeed, conflicting results have been reported regarding a direct role of Akt in vascular permeability (Six et al., 2002; Chen et al., 2005), and its involvement has frequently been inferred by the need for activation of the Akt substrate eNOS during VEGF-mediated permeability (Takahashi and Shibuya, 2005). However, in cerebral MVECs, eNOS activation can also occur in an Akt-independent manner, in particular during inflammation (Martinelli et al., 2009). We did not find a role for VEGFR1 in mediating acute brain permeability either. This receptor has been proposed to mediate permeability in a more chronic and auxiliary way (Takahashi and Shibuya, 2005; Koch et al., 2011), which would not likely be captured in our model systems. In addition, a direct role of VEGFR1 in permeability may be restricted to nonneural vascular beds (Odorisio et al., 2002).

Instead, and in line with it activating Akt, a kinase regarded as synonymous to the cell-survival response (Datta et al., 1999), we found a clear role of VEGFR1 in cytoprotection. A cytoprotective role of VEGF-A for ECs is well documented (Gerber et al., 1999), with most mechanistic evidence derived from in vitro culture models. For instance, in umbilical vein ECs, the main VEGF receptor mediating survival appears to be VEGFR2 (Gerber et al., 1998; dela Paz et al., 2012), whereas in microvascular retinal or dermal ECs there is evidence that VEGFR1 is involved (Cai et al., 2003; Zhang et al., 2010), suggesting that receptor usage may differ between vascular beds. Whereas we were able to find clear experimental support for VEGFR2-mediated permeability in vitro and in vivo, the cytoprotective function of VEGFR1 has so far only been observed in cultured cerebral MVECs. Our failure to induce measurable apoptosis in retinal ECs in vivo through staurosporine and VEGF-A withdrawal suggests that additional

particles located within ca. 20 nm of the plasma membrane (as indicated by arrowheads) in five independent sections, each comprising at least 10 μm of continuous plasma membrane. The scale bars represent 100 nm.

(C) VEGF receptor distribution was also analyzed in postconfluent rat cerebral MVECs by biotinylation of either the apical or, in the presence of EDTA, the apical and basal membranes (ap+bl). Biotinylated proteins were isolated and analyzed by western blot. Shown are representative immunoblots of VEGFR1 and VEGFR2 and quantitative distribution analysis from three experiments (means ± SEM).

(D) VEGFR1 but not -R2 antibodies bound to the retinal vasculature of mice within 5 min of luminal delivery through cardiac injection. However, in the same animals, both VEGFR1 and -R2 antibodies were found bound to alveolar microvessels in the lung. Inversely, when unfixed, nonpermeabilized retinae were incubated with VEGFR antibodies (abluminal), only VEGFR2 was found to stain microvessels significantly. All whole mounts and sections were counterstained with the vessel marker isolectin B4 and analyzed by confocal microcopy. Shown are optical sections of ca. 8 μm thickness of the retinal ganglion cell layer or the center of the lung. The scale bars represent 20 μm.

See also Figure S2 and Table S1.

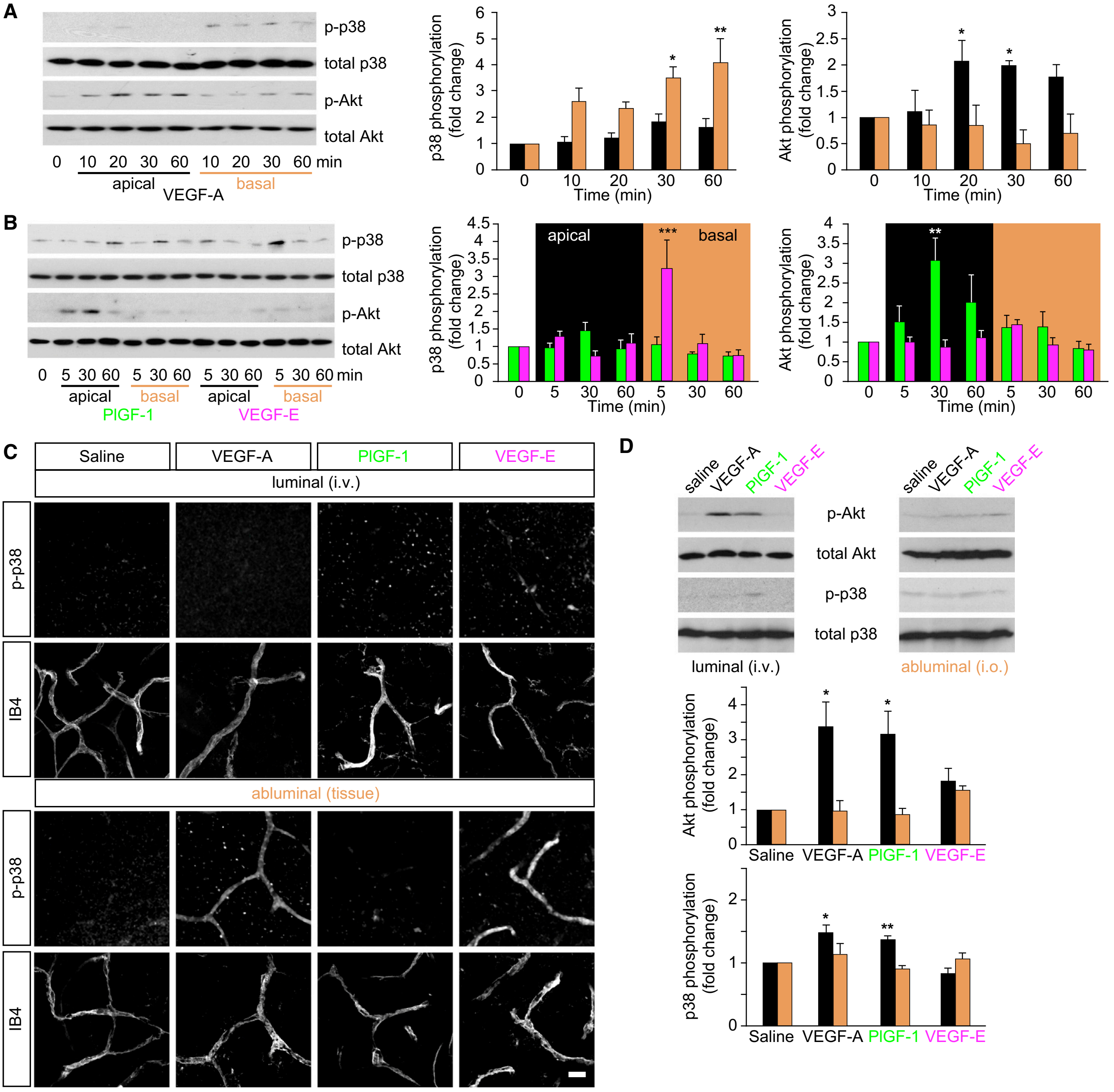

Figure 4. Differential VEGF Receptor Signaling in Brain MVECs and Neural Microvessels

(A) Basal but not apical VEGF-A (50 ng/ml) induced significant activation of p38 but not Akt in postconfluent primary rat brain MVECs. In contrast, Akt but not p38 was activated in response to apical VEGF-A. At the indicated times of VEGF-A stimulation, whole-cell lysates were prepared and levels of phosphorylated p38 (pT180/Y182) and Akt (pS473) were determined by western blotting. Shown are representative blots and normalized densitometric quantifications.

(B) Kinase response to PlGF-1 or VEGF-E analyzed as in (A). p38 was activated within 5 min of basal but not apical VEGF-E (50 ng/ml) treatment, whereas slower Akt activation was seen in response to apical but not basal PlGF-1 (50 ng/ml).

(C) Intravenously injected VEGF-A, PlGF-1, or VEGF-E (at 120 μg/kg) (luminal) did not induce activation of p38 in the pial vasculature of P23 rats. When applied abluminally to the pial microvasculature (at 100 ng/ml) (abluminal), VEGF-A and VEGF-E but not PlGF-1 led to rapid activation of p38. Pial tissues were fixed within 5 min of treatment and then stained for phosphorylated p38 (pT180/Y182). All sections were counterstained with the vessel marker isolectin B4 (IB4) and analyzed by confocal microscopy. Shown are projections spanning a thickness of ca. 11 μm. The scale bar represents 20 μm.

(D) VEGF-A, PlGF-1, or VEGF-E was either injected into the tail vein of P23 rats (at 120 μg/kg) or into the vitreous (i.o.; 100 ng/eye). After ca. 20 min, retinae were isolated and subjected to quantitative immunoblot analysis as described for (A) and (B). Akt was robustly activated in response to i.v. but not i.o. injected VEGF-A and PlGF-1. p38 was weakly activated as well. VEGF-E did not induce any significant effects.

Shown are means ± SEM from at least three independent experiments. $^*p < 0.05$, $^{**}p < 0.01$, $^{***}p < 0.001$ [ANOVA and Dunnett's post hoc test (A, B, and D)]. See also Figure S3.

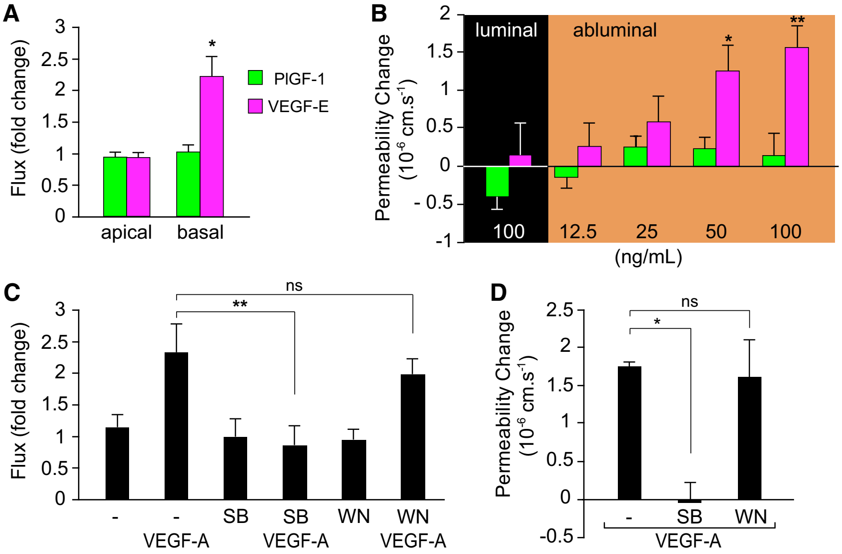

Figure 5. VEGFR2 and p38 Mediate the Brain Microvascular Permeability Response

(A) Transendothelial flux of 4 kDa dextran across confluent primary rat brain MVECs was increased in response to basal but not apical VEGF-E (50 ng/ml). PlGF-1 (50 ng/ml) did not affect flux.

(B) Application of VEGF-E but not PlGF-1 to the abluminal, extravascular space of single pial microvessels in vivo produced a dose-dependent increase of permeability to sulforhodamine B (580 Da). Intracarotid (i.e., luminal) bolus injection of PlGF-1 or VEGF-E did not affect permeability.

(C) Flux measurements in primary brain MVECs showed that pretreatment with 10 μM p38 inhibitor SB202190 (SB) but not the PI3K inhibitor wortmannin (WN) inhibited the permeability response to 50 ng/ml VEGF-A.

(D) VEGF-A-induced permeability changes in pial microvessels were abolished by pretreatment with 10 μM SB202190 but not wortmannin.

Shown are means ± SEM from at least three independent experiments. *$p < 0.05$, **$p < 0.01$; ns, not significant [Student's t test (A), ANOVA and Dunnett's post hoc test (B and D), and two-way ANOVA and Bonferroni's post hoc test (C)].

See also Figure S4.

growth (Walshe et al., 2009) or environmental factors such as flow (dela Paz et al., 2012; Dimmeler and Zeiher, 2000) contribute to EC survival, in particular in the brain and the eye (Ueno et al., 2008). Numerous experiments also show that the apoptotic response of ECs in vivo is relatively slow and only measurable after several days (Meeson et al., 1999; Sugimoto et al., 2003), and thus not within the time frame of our current experimental setup.

Overall, our findings are compatible with previous reports but clearly identify a high degree of specialization of VEGF signal transduction at blood-neural barriers. Specification of VEGF-A action occurs through many mechanisms, including the creation of functionally distinct protein isoforms, the presence of multiple surface, soluble, and coreceptors, and the sequestration of the ligand in the extracellular matrix (Koch et al., 2011). In addition, our data suggest that ECs are able to control the response to VEGF-A through compartmentalizing cellular signaling, and provide an intriguing explanation of how blood-neural barriers can be maintained despite constant exposure to circulating VEGF-A. Finally, the ability to map the vascular permeability response to a distinct topological signaling domain within the EC provides a new opportunity to specifically target pathological aspects of VEGF-A while preserving essential cardiovascular functions.

EXPERIMENTAL PROCEDURES

Animals

Wistar and Lewis rats and C75BL/6J mice were purchased from Harlan Laboratories. All procedures were performed in accordance with UK and Ireland Animal Welfare Acts and with the Association for Research in Vision and Ophthalmology Statement for the Use of Animals in Ophthalmic and Vision Research and the Animal Welfare and the Ethical Review Bodies of the UCL Institute of Ophthalmology, King's College London, and Trinity College Dublin.

Recombinant VEGFs

Recombinant rat VEGF-A(165) was purchased from R&D Systems, VEGF-E was from Cell Sciences (or a kind gift from Kurt Ballmer-Hofer), and human PlGF-1 was from PeproTech.

Magnetic Resonance Imaging

Vascular permeability in lungs, brain, and eyes was assessed in vivo via MRI (Campbell et al., 2012) using a dedicated small rodent 7 T MRI system (http://www.tcd.ie/neuroscience/infrastructure/neuroimaging/index.php#7tesla).

In Vivo Permeability Measurements

The pial microvasculature of Wistar rats (age 25–30 days) was exposed and the permeability was measured as described previously (Easton and Fraser, 1994; Easton et al., 1997).

MVEC Isolation

Microvessels were isolated from rat or mouse cortical gray matter or rat or porcine retinae by collagenase dispase digestion and BSA and Percoll density gradient centrifugation (Abbott et al., 1992). Purified vessels were seeded onto collagen IV/fibronectin-coated tissue-culture ware or Costar Transwells (3460) at high density (vessels from 6 rat brains or 12 retinae per 40 cm^2 or 3 cm^2, respectively). Cells were grown in EGM2-MV (Lonza) (with 5 μg/ml puromycin during the first 5 days; Perrière et al., 2007) for 2–3 weeks until their TEER plateaued at values above 200 Ω/cm^2.

Immunocytochemistry

MVECs were grown on collagen IV/fibronectin-coated tissue-culture ware or 12 mm Costar Transwell filters. Cells were fixed using 3.7% formaldehyde and extracted in acetone (−20°C). Alternatively, they were fixed and permeabilized simultaneously in 80% MeOH, 3.2% formaldehyde, 50 mM HEPES (pH 7.4) (Martins et al., 2013). Staining was performed as previously described (Turowski et al., 2004) using antibodies against von Willebrand factor (Dako), VE-cadherin (Martins et al., 2013), occludin and Cldn5 (Invitrogen), VEGFR1 (sc-31173; Santa Cruz Biotechnology), VEGFR2 (ab11939; Abcam), and P-glycoprotein (clone C219).

Immunogold Electron Microscopy

Paraformaldehyde (PFA)-fixed brains from 10-month-old mice were isolated and 50 μm hippocampal slices were dissected out and postfixed for 2 hr in 4% PFA. MVECs on Transwell filter inserts were fixed in 4% PFA and 0.1% glutaraldehyde. Fixed samples were processed as previously described (Eden et al., 2010). Briefly, samples were embedded in 12% gelatin in 0.1 M phosphate buffer (pH 7.4). After infusion with 2.3 M sucrose at 4°C overnight, 80 nm sections were cut at −120°C using a cryoultramicrotome and collected

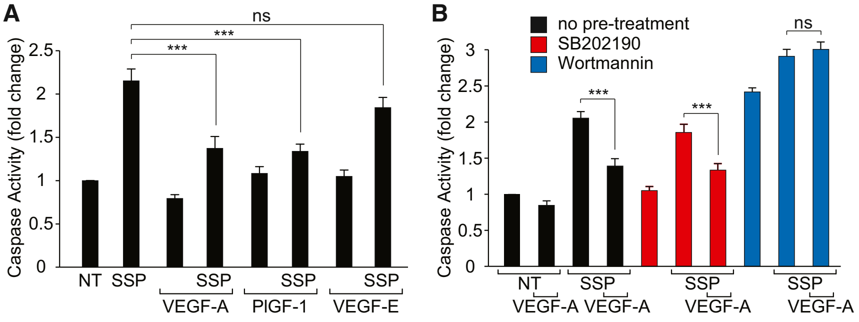

Figure 6. VEGFR1 and Akt-Mediated Cytoprotection in Brain Microvascular ECs

(A) Staurosporine (SSP; 1 μM, 60 min) more than doubled caspase 3/7 activity in primary rat brain MVECs when compared to untreated control cells (NT). The staurosporine-induced caspase 3/7 activity was significantly reduced by pretreatment with VEGF-A and PlGF-1 but not VEGF-E (all at 50 ng/ml for 30 min).

(B) The VEGF-A-mediated reduction of staurosporine-induced caspase 3/7 activity was sensitive to 10 μM PI3K inhibitor wortmannin but not the p38 inhibitor SB202190.

Shown are means ± SEM ($n \geq 15$). ***$p < 0.001$; ns, not significant (Student's t test).

in a 1:1 mixture of 2.3 M sucrose and 2% methylcellulose. The sections were then stained as previously described (Slot et al., 1991) using antibodies against VEGFR1 (sc-31173) (AF471; R&D Systems) and VEGFR2 (ab11939) (DC101). Samples were viewed with a JEOL 1010 transmission electron microscope, and images were gathered using a Gatan Orius SC100B charge-coupled device camera. Image manipulation was performed in Gatan Digital Micrograph and Adobe Photoshop. VEGFR distribution was determined by visual inspection of electron micrographs. Gold particles were considered luminal (apical) or abluminal (basal) when found within 20 nm of the respective plasma membrane (Ottersen, 1989).

Transendothelial Flux

Fluorescein isothiocyanate (FITC) dextran (4 kDa) flux across MVECs was measured as previously described (Martins et al., 2013).

Transendothelial Electrical Resistance

TEER was measured directly with chopstick electrodes (Turowski et al., 2004) and an EVOM voltohmmeter (World Precision Instruments). Alternatively, TEER was assessed by impedance spectroscopy using cells grown either on 12 mm Transwells and a cellZscope (nanoAnalytics) or on gold electrodes (eight-well 8W1E) and an ECIS (4,000 Hz) (Applied BioPhysics).

Cytoprotection Assays

Caspase activity of MVECs grown in 96-well plates was measured using the Apo-ONE Homogeneous Caspase-3/7 assay kit (Promega).

Cell-Surface Biotinylation

Apical and basal biotinylation was performed using a method adapted from Gottardi et al. (1995). Basal biotinylation was very weak, presumably because of reduced access through the filter and the basement membrane (Gottardi et al., 1995). Therefore, apical and basal domains were labeled simultaneously in the presence of EDTA. Biotinylated proteins were isolated on streptavidin beads and, following immunoblotting for VEGF receptors, apical and basal signals were quantified by densitometry and normalized against input signals. Basal signal was calculated from the combined apical and basal labeling minus the apical signal.

Western Blots

For immunoblot analyses, samples were lysed in 50 mM Tris/Cl (pH 6.8), 2% SDS, 10% glycerol, 100 mM dithiothreitol (DTT), 100 nM calyculin A (50 μl/cm^2 of cells), separated by SDS-PAGE, electrotransferred to nitrocellulose or polyvinylidene fluoride, and immunodecorated with phospho-specific and total antibodies as previously described (Martinelli et al., 2009). Akt (phospho-S473 and total), p38 (phospho-T180/Y182 and total), and anti-VEGFR2 antibodies were from Cell Signaling (55B11), and anti-VEGFR1 was from Abcam (Y103).

VEGF Receptor Localization in Retinal and Pulmonary Vessels

Anti-VEGFR1 (sc-31173) and -VEGFR2 (DC101) antibodies (each at 6 mg/kg) were injected into the left ventricle of anesthetized p20 mice (C57 BL/6J). After 5 min, the animals were perfused with PBS followed by 4% PFA. Retinae were dissected out and processed for whole-mount staining (West et al., 2005). For abluminal detection, dissected unfixed retinae of PBS-perfused mice were overlaid with anti-VEGFR1 and -VEGFR2 antibodies (20 μg/ml) for 5 min, washed three times briefly with PBS, and then fixed in 4% PFA for 1 hr. All retinae were stained using anti-F4/80 (MCA497EL; AbD Serotec) and/or biotinylated isolectin B4 (B1205; Vector Labs) and secondary antibodies to detect bound VEGFR1 and -R2 antibodies.

Transversal (1 mm) sections of lungs from animals having undergone cardiac injections were analyzed in a similar manner. All samples were analyzed by confocal microscopy on a Zeiss LSM 700. Retinal flat mounts were imaged with a pinhole aperture allowing capture of the entire primary plexus depth in a single scan, whereas sections of lung were analyzed from projections of sequential image stacks.

p38 and Akt Signaling In Vivo

For luminal stimulation, 120 μg/kg of VEGF-A, VEGF-E, or PlGF-1 or an equal volume (100 μl) of saline was injected into the tail vein of anesthetized P23 Wistar rats. For abluminal stimulation, cranial windows were surgically introduced and VEGFs were added at 100 ng/ml (final concentration) to the liquid pool superfusing the pial microvasculature. Five minutes after injection/VEGF application, animals were perfused and fixed and pial or lung sections were processed for immunohistochemistry (Powner et al., 2010). For Akt analysis, animals were injected with VEGFs in the tail vein as above or in the vitreous (using 100 ng of VEGF-A, VEGF-E, or PlGF-1 or an equal volume [5 μl] of saline). After ca. 25 min, retinae were isolated and lysed in 50 mM Tris/Cl (pH 6.8), 2% SDS, 10% glycerol, 100 mM DTT, 100 nM calyculin A and subjected to immunoblot analysis.

SUPPLEMENTAL INFORMATION

Supplemental Information includes Supplemental Experimental Procedures, four figures, and one table and can be found with this article online at http://dx.doi.org/10.1016/j.devcel.2014.06.027.

ACKNOWLEDGMENTS

We thank Ms. Eva Kubala (UCL Institute of Ophthalmology) for initial experiments on VEGF receptor localization in the retina, Dr. Robert Baxter (University of Sydney) for purified IGFBP3, Prof. Kurt Ballmer-Hofer (Paul Scherrer Institut) for purified VEGF-E, Prof. Gunnar Gouras (University of Lund) for mouse hippocampal sections, and Drs. Pierre-Olivier Couraud and Nicolas Perrière (Institut Cochin) and Prof. Christiana Ruhrberg (UCL Institute of Ophthalmology) for stimulating discussions and data sharing ahead of publication. This work was supported by the British Heart Foundation (PG/11/62/29010) and the Special Trustees of Moorfields Eye Hospital.

Received: December 13, 2013
Revised: June 5, 2014
Accepted: June 30, 2014
Published: August 28, 2014

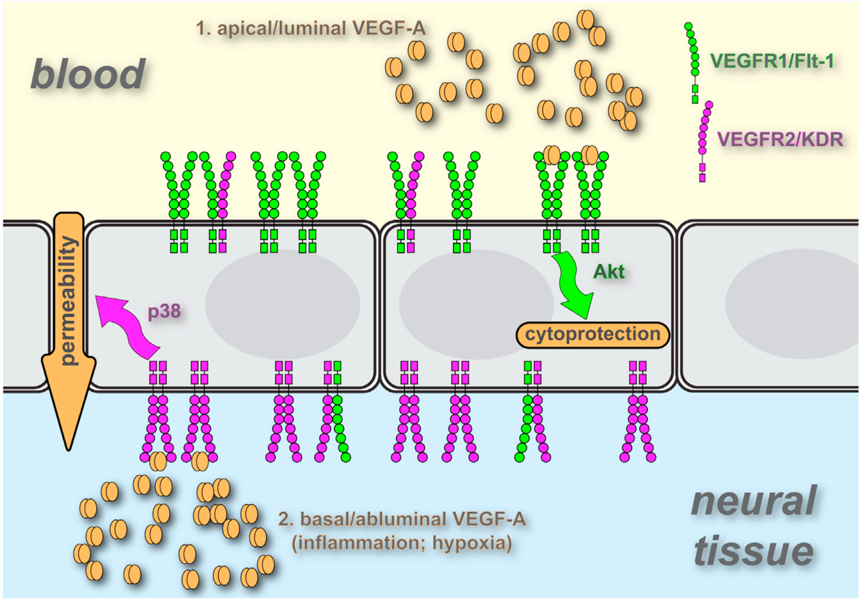

Figure 7. Model of VEGF Action on MVECs of Blood-Neural Barriers

The majority of VEGFR1 and VEGFR2 is found on the apical (luminal) and basal (abluminal) side, respectively, of brain or retinal MVECs. Based on computer simulation (Mac Gabhann and Popel, 2007), such a receptor distribution is predicted to result primarily in VEGFR1 and VEGFR2 homodimers at the apical and basal surface, respectively. Residual apical VEGFR2 or basal VEGFR1 would not be found in homodimers but rather in heterodimers with the more abundant receptor. Signaling from VEGFR1, mainly triggered by circulating VEGF, activates a PI3K/Akt pathway, which plays a role in endothelial cytoprotection. Signaling from VEGFR2, triggered by tissue-borne VEGF, activates p38 and mediates paracellular permeability, hallmarked by macromolecular flux, electrical conductance, and a loss of Cldn5.

REFERENCES

Abbott, N.J., Hughes, C.C., Revest, P.A., and Greenwood, J. (1992). Development and characterisation of a rat brain capillary endothelial culture: towards an in vitro blood-brain barrier. J. Cell Sci. *103*, 23–37.

Abbott, N.J., Patabendige, A.A., Dolman, D.E., Yusof, S.R., and Begley, D.J. (2010). Structure and function of the blood-brain barrier. Neurobiol. Dis. *37*, 13–25.

Argaw, A.T., Asp, L., Zhang, J., Navrazhina, K., Pham, T., Mariani, J.N., Mahase, S., Dutta, D.J., Seto, J., Kramer, E.G., et al. (2012). Astrocyte-derived VEGF-A drives blood-brain barrier disruption in CNS inflammatory disease. J. Clin. Invest. *122*, 2454–2468.

Bates, D.O., and Curry, F.E. (1996). Vascular endothelial growth factor increases hydraulic conductivity of isolated perfused microvessels. Am. J. Physiol. *271*, H2520–H2528.

Becker, P.M., Waltenberger, J., Yachechko, R., Mirzapoiazova, T., Sham, J.S., Lee, C.G., Elias, J.A., and Verin, A.D. (2005). Neuropilin-1 regulates vascular endothelial growth factor-mediated endothelial permeability. Circ. Res. *96*, 1257–1265.

Butt, A.M., Jones, H.C., and Abbott, N.J. (1990). Electrical resistance across the blood-brain barrier in anaesthetized rats: a developmental study. J. Physiol. *429*, 47–62.

Cai, J., Ahmad, S., Jiang, W.G., Huang, J., Kontos, C.D., Boulton, M., and Ahmed, A. (2003). Activation of vascular endothelial growth factor receptor-1 sustains angiogenesis and Bcl-2 expression via the phosphatidylinositol 3-kinase pathway in endothelial cells. Diabetes *52*, 2959–2968.

Campbell, M., Hanrahan, F., Gobbo, O.L., Kelly, M.E., Kiang, A.S., Humphries, M.M., Nguyen, A.T., Ozaki, E., Keaney, J., Blau, C.W., et al. (2012). Targeted suppression of claudin-5 decreases cerebral oedema and improves cognitive outcome following traumatic brain injury. Nat. Commun. *3*, 849.

Chen, H.X., and Cleck, J.N. (2009). Adverse effects of anticancer agents that target the VEGF pathway. Nat. Rev. Clin. Oncol. *6*, 465–477.

Chen, J., Somanath, P.R., Razorenova, O., Chen, W.S., Hay, N., Bornstein, P., and Byzova, T.V. (2005). Akt1 regulates pathological angiogenesis, vascular maturation and permeability in vivo. Nat. Med. *11*, 1188–1196.

Cudmore, M.J., Hewett, P.W., Ahmad, S., Wang, K.Q., Cai, M., Al-Ani, B., Fujisawa, T., Ma, B., Sissaoui, S., Ramma, W., et al. (2012). The role of heterodimerization between VEGFR-1 and VEGFR-2 in the regulation of endothelial cell homeostasis. Nat. Commun. *3*, 972.

Datta, S.R., Brunet, A., and Greenberg, M.E. (1999). Cellular survival: a play in three Akts. Genes Dev. *13*, 2905–2927.

dela Paz, N.G., Walshe, T.E., Leach, L.L., Saint-Geniez, M., and D'Amore, P.A. (2012). Role of shear-stress-induced VEGF expression in endothelial cell survival. J. Cell Sci. *125*, 831–843.

Dimmeler, S., and Zeiher, A.M. (2000). Endothelial cell apoptosis in angiogenesis and vessel regression. Circ. Res. *87*, 434–439.

Easton, A.S., and Fraser, P.A. (1994). Variable restriction of albumin diffusion across inflamed cerebral microvessels of the anaesthetized rat. J. Physiol. *475*, 147–157.

Easton, A.S., Sarker, M.H., and Fraser, P.A. (1997). Two components of blood-brain barrier disruption in the rat. J. Physiol. *503*, 613–623.

Eden, E.R., White, I.J., Tsapara, A., and Futter, C.E. (2010). Membrane contacts between endosomes and ER provide sites for PTP1B-epidermal growth factor receptor interaction. Nat. Cell Biol. *12*, 267–272.

Ferrara, N., Mass, R.D., Campa, C., and Kim, R. (2007). Targeting VEGF-A to treat cancer and age-related macular degeneration. Annu. Rev. Med. *58*, 491–504.

Foxton, R.H., Finkelstein, A., Vijay, S., Dahlmann-Noor, A., Khaw, P.T., Morgan, J.E., Shima, D.T., and Ng, Y.S. (2013). VEGF-A is necessary and sufficient for retinal neuroprotection in models of experimental glaucoma. Am. J. Pathol. *182*, 1379–1390.

Gerber, H.P., McMurtrey, A., Kowalski, J., Yan, M., Keyt, B.A., Dixit, V., and Ferrara, N. (1998). Vascular endothelial growth factor regulates endothelial cell survival through the phosphatidylinositol 3′-kinase/Akt signal transduction pathway. Requirement for Flk-1/KDR activation. J. Biol. Chem. *273*, 30336–30343.

Gerber, H.P., Hillan, K.J., Ryan, A.M., Kowalski, J., Keller, G.A., Rangell, L., Wright, B.D., Radtke, F., Aguet, M., and Ferrara, N. (1999). VEGF is required for growth and survival in neonatal mice. Development *126*, 1149–1159.

Gottardi, C.J., Dunbar, L.A., and Caplan, M.J. (1995). Biotinylation and assessment of membrane polarity: caveats and methodological concerns. Am. J. Physiol. *268*, F285–F295.

Issbrücker, K., Marti, H.H., Hippenstiel, S., Springmann, G., Voswinckel, R., Gaumann, A., Breier, G., Drexler, H.C., Suttorp, N., and Clauss, M. (2003). p38 MAP kinase—a molecular switch between VEGF-induced angiogenesis and vascular hyperpermeability. FASEB J. *17*, 262–264.

Kabir, J., Lobo, M., and Zachary, I. (2002). Staurosporine induces endothelial cell apoptosis via focal adhesion kinase dephosphorylation and focal adhesion disassembly independent of focal adhesion kinase proteolysis. Biochem. J. *367*, 145–155.

Koch, S., Tugues, S., Li, X., Gualandi, L., and Claesson-Welsh, L. (2011). Signal transduction by vascular endothelial growth factor receptors. Biochem. J. *437*, 169–183.

Lizama, C.O., and Zovein, A.C. (2013). Polarizing pathways: balancing endothelial polarity, permeability, and lumen formation. Exp. Cell Res. *319*, 1247–1254.

Mac Gabhann, F., and Popel, A.S. (2007). Dimerization of VEGF receptors and implications for signal transduction: a computational study. Biophys. Chem. *128*, 125–139.

Maharaj, A.S., and D'Amore, P.A. (2007). Roles for VEGF in the adult. Microvasc. Res. *74*, 100–113.

Martinelli, R., Gegg, M., Longbottom, R., Adamson, P., Turowski, P., and Greenwood, J. (2009). ICAM-1-mediated endothelial nitric oxide synthase activation via calcium and AMP-activated protein kinase is required for transendothelial lymphocyte migration. Mol. Biol. Cell *20*, 995–1005.

Martins, T., Burgoyne, T., Kenny, B.A., Hudson, N., Futter, C.E., Ambrósio, A.F., Silva, A.P., Greenwood, J., and Turowski, P. (2013). Methamphetamine-induced nitric oxide promotes vesicular transport in blood-brain barrier endothelial cells. Neuropharmacology *65*, 74–82.

Meeson, A.P., Argilla, M., Ko, K., Witte, L., and Lang, R.A. (1999). VEGF deprivation-induced apoptosis is a component of programmed capillary regression. Development *126*, 1407–1415.

Merrill, M.J., and Oldfield, E.H. (2005). A reassessment of vascular endothelial growth factor in central nervous system pathology. J. Neurosurg. *103*, 853–868.

Miller, J.W., Le Couter, J., Strauss, E.C., and Ferrara, N. (2013). Vascular endothelial growth factor A in intraocular vascular disease. Ophthalmology *120*, 106–114.

Nagy, J.A., Benjamin, L., Zeng, H., Dvorak, A.M., and Dvorak, H.F. (2008). Vascular permeability, vascular hyperpermeability and angiogenesis. Angiogenesis *11*, 109–119.

Nitta, T., Hata, M., Gotoh, S., Seo, Y., Sasaki, H., Hashimoto, N., Furuse, M., and Tsukita, S. (2003). Size-selective loosening of the blood-brain barrier in claudin-5-deficient mice. J. Cell Biol. *161*, 653–660.

Odorisio, T., Schietroma, C., Zaccaria, M.L., Cianfarani, F., Tiveron, C., Tatangelo, L., Failla, C.M., and Zambruno, G. (2002). Mice overexpressing placenta growth factor exhibit increased vascularization and vessel permeability. J. Cell Sci. *115*, 2559–2567.

Ottersen, O.P. (1989). Quantitative electron microscopic immunocytochemistry of neuroactive amino acids. Anat. Embryol. (Berl.) *180*, 1–15.

Perrière, N., Yousif, S., Cazaubon, S., Chaverot, N., Bourasset, F., Cisternino, S., Declèves, X., Hori, S., Terasaki, T., Deli, M., et al. (2007). A functional in vitro model of rat blood-brain barrier for molecular analysis of efflux transporters. Brain Res. *1150*, 1–13.

Powner, M.B., Gillies, M.C., Tretiach, M., Scott, A., Guymer, R.H., Hageman, G.S., and Fruttiger, M. (2010). Perifoveal Müller cell depletion in a case of macular telangiectasia type 2. Ophthalmology *117*, 2407–2416.

Saint-Geniez, M., Maharaj, A.S., Walshe, T.E., Tucker, B.A., Sekiyama, E., Kurihara, T., Darland, D.C., Young, M.J., and D'Amore, P.A. (2008). Endogenous VEGF is required for visual function: evidence for a survival role on Müller cells and photoreceptors. PLoS ONE *3*, e3554.

Sarker, M.H., Easton, A.S., and Fraser, P.A. (1998). Regulation of cerebral microvascular permeability by histamine in the anaesthetized rat. J. Physiol. *507*, 909–918.

Sarker, M.H., Hu, D.E., and Fraser, P.A. (2010). Regulation of cerebromicrovascular permeability by lysophosphatidic acid. Microcirculation *17*, 39–46.

Six, I., Kureishi, Y., Luo, Z., and Walsh, K. (2002). Akt signaling mediates VEGF/VPF vascular permeability in vivo. FEBS Lett. *532*, 67–69.

Slot, J.W., Geuze, H.J., Gigengack, S., Lienhard, G.E., and James, D.E. (1991). Immuno-localization of the insulin regulatable glucose transporter in brown adipose tissue of the rat. J. Cell Biol. *113*, 123–135.

Steed, E., Balda, M.S., and Matter, K. (2010). Dynamics and functions of tight junctions. Trends Cell Biol. *20*, 142–149.

Sugimoto, H., Hamano, Y., Charytan, D., Cosgrove, D., Kieran, M., Sudhakar, A., and Kalluri, R. (2003). Neutralization of circulating vascular endothelial growth factor (VEGF) by anti-VEGF antibodies and soluble VEGF receptor 1 (sFlt-1) induces proteinuria. J. Biol. Chem. *278*, 12605–12608.

Sun, Z., Li, X., Massena, S., Kutschera, S., Padhan, N., Gualandi, L., Sundvold-Gjerstad, V., Gustafsson, K., Choy, W.W., Zang, G., et al. (2012). VEGFR2 induces c-Src signaling and vascular permeability in vivo via the adaptor protein TSAd. J. Exp. Med. *209*, 1363–1377.

Takahashi, H., and Shibuya, M. (2005). The vascular endothelial growth factor (VEGF)/VEGF receptor system and its role under physiological and pathological conditions. Clin. Sci. *109*, 227–241.

Turowski, P., Adamson, P., Sathia, J., Zhang, J.J., Moss, S.E., Aylward, G.W., Hayes, M.J., Kanuga, N., and Greenwood, J. (2004). Basement membrane-dependent modification of phenotype and gene expression in human retinal pigment epithelial ARPE-19 cells. Invest. Ophthalmol. Vis. Sci. *45*, 2786–2794.

Ueno, S., Pease, M.E., Wersinger, D.M., Masuda, T., Vinores, S.A., Licht, T., Zack, D.J., Quigley, H., Keshet, E., and Campochiaro, P.A. (2008). Prolonged blockade of VEGF family members does not cause identifiable damage to retinal neurons or vessels. J. Cell. Physiol. *217*, 13–22.

Walshe, T.E., Saint-Geniez, M., Maharaj, A.S., Sekiyama, E., Maldonado, A.E., and D'Amore, P.A. (2009). TGF-β is required for vascular barrier function, endothelial survival and homeostasis of the adult microvasculature. PLoS ONE *4*, e5149.

Wang, J., Xu, X., Elliott, M.H., Zhu, M., and Le, Y.Z. (2010). Müller cell-derived VEGF is essential for diabetes-induced retinal inflammation and vascular leakage. Diabetes *59*, 2297–2305.

Weidemann, A., Krohne, T.U., Aguilar, E., Kurihara, T., Takeda, N., Dorrell, M.I., Simon, M.C., Haase, V.H., Friedlander, M., and Johnson, R.S. (2010). Astrocyte hypoxic response is essential for pathological but not developmental angiogenesis of the retina. Glia *58*, 1177–1185.

Weis, S.M., and Cheresh, D.A. (2005). Pathophysiological consequences of VEGF-induced vascular permeability. Nature *437*, 497–504.

West, H., Richardson, W.D., and Fruttiger, M. (2005). Stabilization of the retinal vascular network by reciprocal feedback between blood vessels and astrocytes. Development *132*, 1855–1862.

Zhang, Z., Neiva, K.G., Lingen, M.W., Ellis, L.M., and Nör, J.E. (2010). VEGF-dependent tumor angiogenesis requires inverse and reciprocal regulation of VEGFR1 and VEGFR2. Cell Death Differ. *17*, 499–512.

Developmental Cell, Volume *30*

Supplemental Information

Differential Apicobasal VEGF Signaling at Vascular Blood-Neural Barriers

Natalie Hudson, Michael B. Powner, Mosharraf H. Sarker, Thomas Burgoyne, Matthew Campbell, Zoe K. Ockrim, Roberta Martinelli, Clare E. Futter, Maria B. Grant, Paul A. Fraser, David T. Shima, John Greenwood, and Patric Turowski

Figure S1

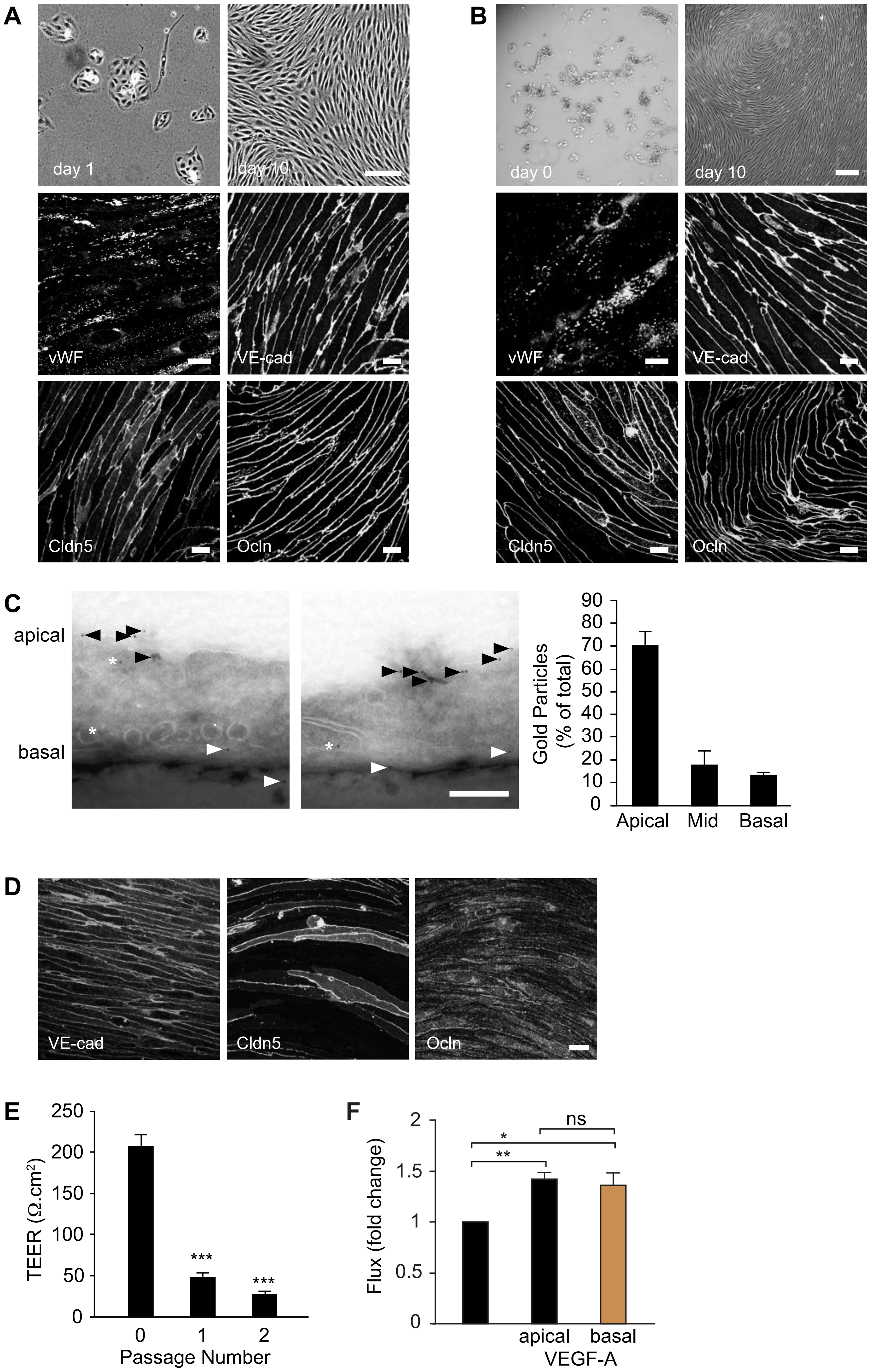

A
day 1
day 10
vWF
VE-cad
Cldn5
Ocln
B
day 0
day 10
vWF
VE-cad
Cldn5
Ocln
C
apical
basal
Gold Particles (% of total)
90
80
70
60
50
40
30
20
10
0
Apical
Mid
Basal
D
VE-cad
Cldn5
Ocln
E
TEER (Ω.cm2)
250
200
150
100
50
0
***
***
0
1
2
Passage Number
F
Flux (fold change)
2
1.5
1
0.5
0
ns
*
**
apical
basal
VEGF-A

Figure S2

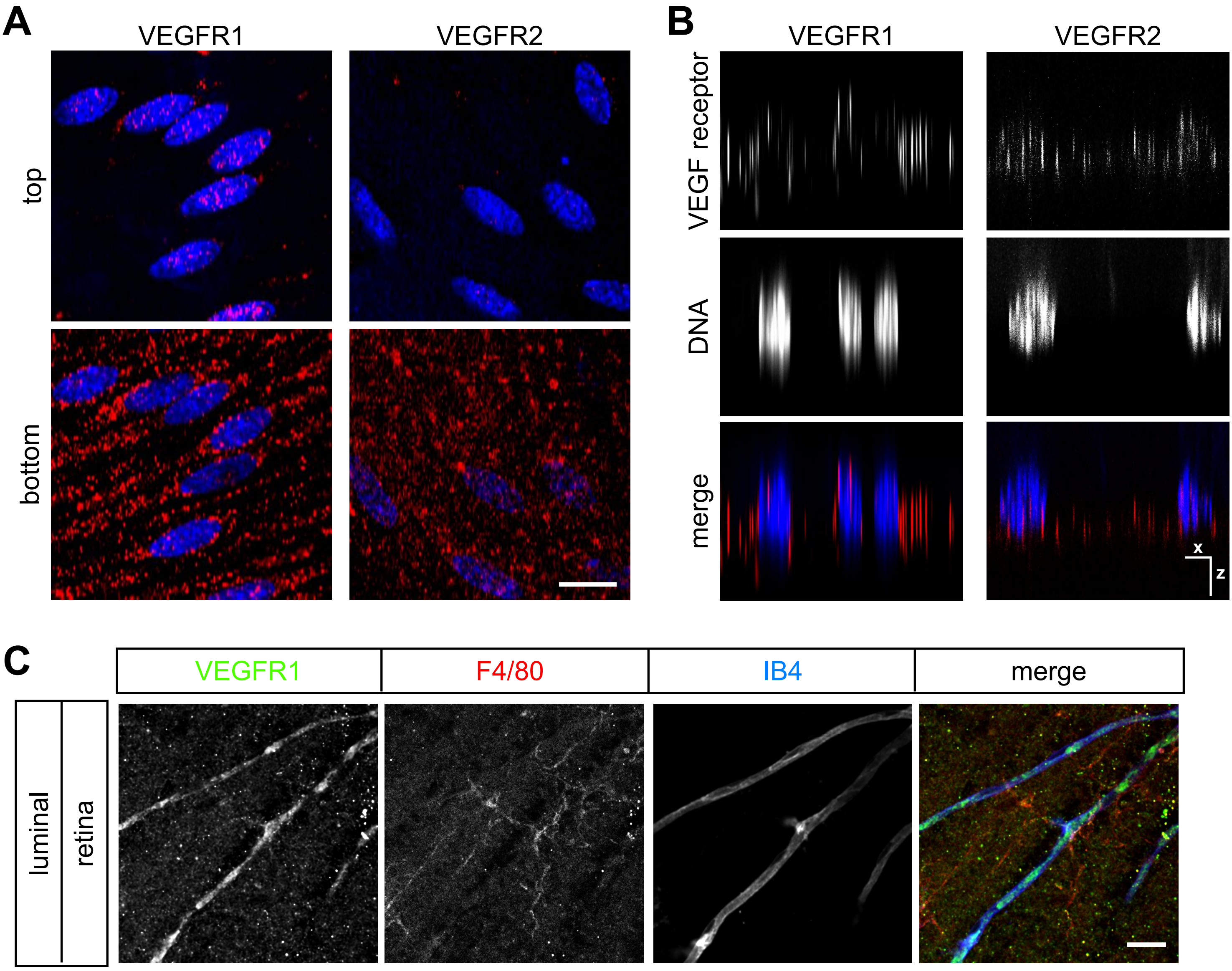

A
VEGFR1
VEGFR2
top
bottom
B
VEGFR1
VEGFR2
VEGF receptor
DNA
merge
x
z
C
VEGFR1
F4/80
IB4
merge
luminal
retina

Figure S3

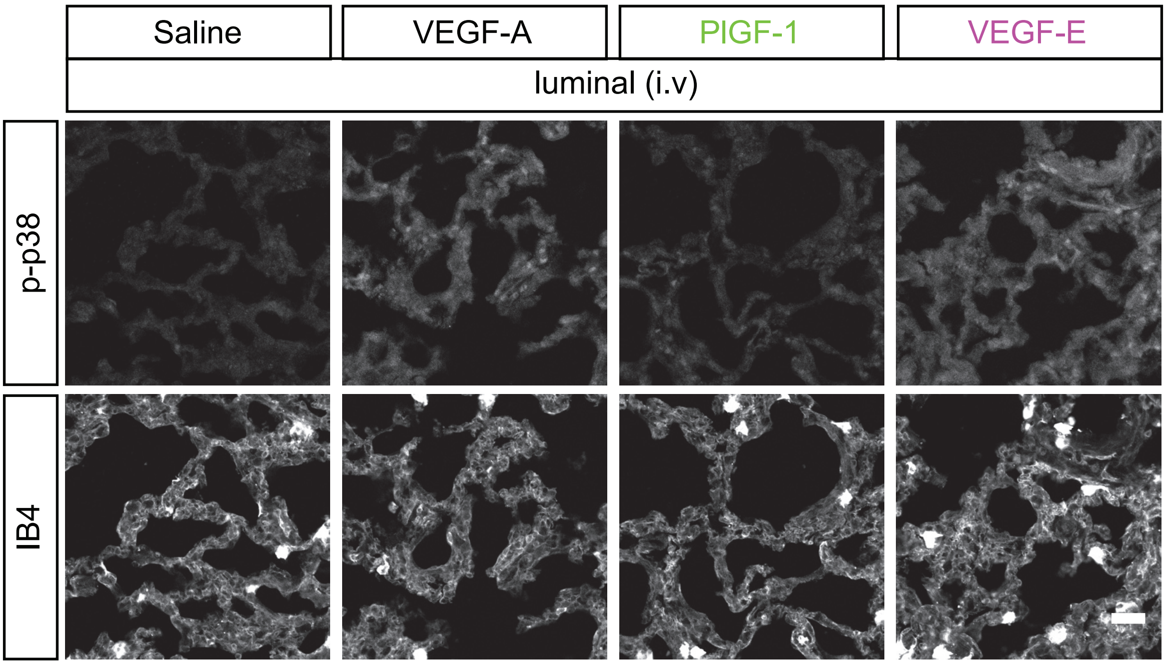

Figure S4

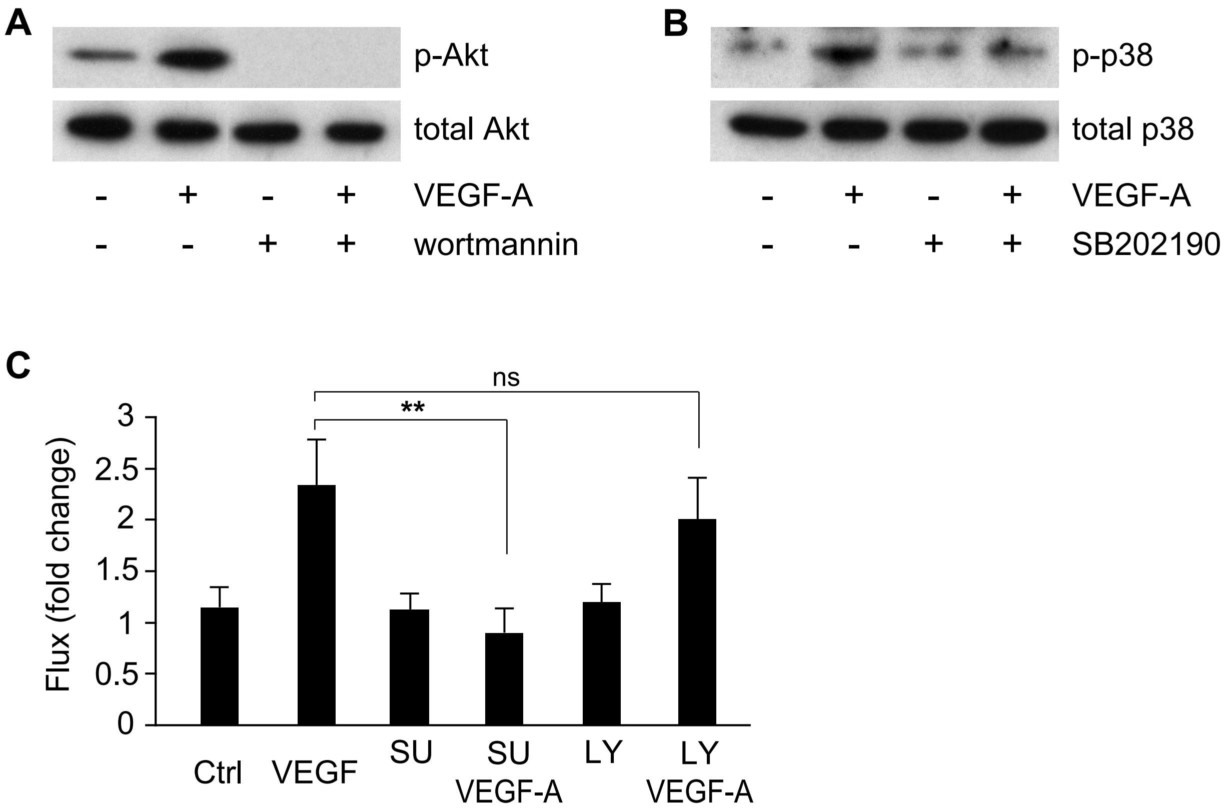
A
p-Akt
total Akt
- + - + VEGF-A
- - + + wortmannin
B
p-p38
total p38
- + - + VEGF-A
- - + + SB202190
C
ns
**
Flux (fold change)
3
2.5
2
1.5
1
0.5
0
Ctrl
VEGF
SU
SU VEGF-A
LY
LY VEGF-A

Supplemental Figure Legends

Figure S1, related to Figure 2: Characterization of primary rat brain and retinal MVECs.

(**A, B**) Microvessels were isolated from Lewis rat brains (A) or retinae (B) and seeded onto collagen IV/fibronectin coated dishes. Phase contrast microscopy showed that MVECs formed monolayers within 10 days. Scale bars, 100 µm. Cultures were also further characterized for the expression and distribution of von Willebrand Factor (vWF), VE-cad, Cldn5 and Occludin (Ocln) by indirect immunocytochemistry and confocal microscopy. Shown are full projections spanning the entirety of the cell monolayer. Scale bars, 10 µm. By all cytological criteria cerebral and retinal MVEC were indistinguishable and as expected junctional protein localization was restricted to intercellular contact areas. In agreement, barrier properties of cultures were very well developed. TEER reached a maximum of 345 ± 30 $\Omega.cm^2$ (n = 11 brain cultures) two weeks after vessel seeding. At the same time we measured very low macromolecular flux (1.883 ± 0.267 10^{-6} cm/s for 4 kDa FITC dextran), which so far has only been observed when brain MVECs are co-cultured with astrocytes (Perriere et al., 2007). (**C**) Rat brain EC monolayers were also analyzed for the distribution of p-glycoprotein by cryoimmuno-EM. Gold particles in the apical, internal or basal areas are indicated by black arrowheads, asterisks or white arrowheads, respectively. Scale bar, 200 nm. The panel on the right depicts quantification of gold particle distribution determined from three independent preparations and shows that p-glycoprotein, which is known to be enriched on the luminal side of BBB endothelium *in vivo* (Miller, 2010), localized predominantly to the apical face of our EC layers suggesting that significant apical-basal polarity was maintained. Importantly, barrier determinants of our MVEC preparations, in particular uninterrupted tight junction distribution and high TEER, were lost when cells were sub-cultured (see panels D-F). Consequently, all subsequent experiments were conducted on directly plated primary MVECs within two weeks of isolation when TEER was at least 200 $\Omega.cm^2$. (**D**) Microvessels were isolated from rat brains and grown on collagen IV/fibronectin coated dishes. Cells were passaged by trypsinization on three occasions (at 10-day intervals) and then allowed to reach confluence. Cells were then fixed, decorated using antibodies against VE-cad, Cldn5

and Ocln and analyzed by indirect immunofluorescence and confocal microscopy. Shown are full projections spanning the entirety of the cell monolayer. Scale bar, 10 µm. (**E**) TEER across confluent brain MVEC monolayers 14 days after passage 0, 1 and 2 (means ± SEM, n = 3). ***P < 0.001 (ANOVA and Dunnett's post hoc test). (**F**) The rat retinal MVEC cell line PT2 (details available on request) was passaged 9 times after immortalization and grown on permeable transwell inserts until confluent. Flux of 4 kDa FITC dextran was measured in the absence or presence of apical or basal VEGF-A (50 ng/ml). No predilection of apical or basal stimulation was observed. Similar results were obtained using the human or rat brain MVEC cell lines CMEC/D3 (Weksler et al., 2005) or GPNT (Romero et al., 2003) (data not shown). Shown are means ± SEM of three independent experiments. Statistical analysis was by Student's t-test with P > 0.05 (ns), *P < 0.05, **P < 0.01.

Figure S2, related to Figure 3: VEGF receptor distribution in MVECs.

(**A, B**) Confocal analysis of VEGF receptor distribution in primary brain MVECs. Rat cerebral MVECs were grown on permeable transwell inserts until they reached a TEER of greater than 200 $\Omega.cm^2$. Cells were fixed with a mixture of MeOH and formaldehyde and stained for VEGF receptors (red) using anti-R1 (sc-31173) or –R2 (ab11939). Distribution was analyzed by confocal microscopy. MVECs are extremely thin and apical or basal distribution cannot be determined in areas other than nuclei (DNA, blue). Shown in (A) are 400 nm sections through the very top or the bottom of cells, illustrating that in the nuclear area R1 is predominately apical whereas R2 is found underneath nuclei. Scale bar, 10 µm. Shown in (B) are representative z-section through nuclei (DNA, blue) illustrating significant VEGF receptor (red) staining for R1 on top of nuclei and for R2 below nuclei. Scale bars: x, 10 µm; z, 1 µm. **(C)** Circulating anti-VEGFR1 was not bound to monocytes or macrophages in the mouse retina. VEGFR1 (sc-31173) antibody was delivered into the vasculature of mice by cardiac injection and left to circulate for 5 min as described for Figure 3 C. After perfusion with PBS and fixation, retinae were dissected and permeabilized. Whole mount retinae were then processed with fluorescein-conjugated anti-goat antibodies to reveal bound anti-R1 antibodies and co-staining for the monocyte/macrophage marker F4/80 (red) and isolectin B4 (IB4, blue) and analyzed by confocal microcopy. Scale Bar, 20 µm.

Figure S3, related to Figure 4: p38 activation in lung microvessels *in vivo* in response to circulating VEGFs.

Intravenously injected VEGF-A and VEGF-E (at 120 µg/kg)(luminal) induce rapid and strong activation of p38 in the alveolar microvasculature of P23 rats. PlGF-1 injection only weakly activated p38. Lungs were fixed within 5 min of treatment and then stained for phosphorylated p38 (pT180/Y182). All sections were counterstained with the vessel marker isolectin B4 (IB4) and analyzed by confocal microcopy. Shown are projections spanning a thickness of ca. 8 µm. N.B.: lungs were from the same animals as the pial sections shown in Figure 4. Bar, 20 µm.

Figure S4, related to Figure 5: Inhibition of VEGF signaling in brain MVECs.

Rat cerebral MVECs were grown on permeable transwell inserts until they reached a TEER of greater than 200 $\Omega.cm^2$. They were then transferred into growth-factor reduced medium for 24 h before treated with 10 µM wortmannin (**A**) or SB202190 (**B**) or left untreated as indicated. Cells were then stimulated in the presence or absence of the inhibitor with 50 ng/ml VEGF-A from both the apical and basal side. After 60 min cells were lysed and analyzed by immunoblots. Shown are representative immunoblots immunodecorated for phospho-S473 and total Akt (A) or phospho-T180/Y182 and total p38 (B). (**C**) Mean (± SEM) 4 kDa FITC dextran flux changes in brain MVEC in response to 50 ng/ml VEGF-A following pre-treatment with 10 µM of the VEGFR2 selective inhibitor SU1498 (SU) or the PI3K inhibitor LY294002 (LY). **$P < 0.01$ (Two-way ANOVA and Bonferroni post hoc).

Table S1, related to Figure 3: Summary of of immunogold EM quantification

	Mouse hippocampal microvessels		Rat brain microvascular endothelial cells		
**antibody**	**VEGFR1 (sc-31173)**	**VEGFR2 (DC101)**	**VEGFR1 (sc-31173)**	**VEGFR1 (AF471)**	**VEGFR2 (ab11939)**
**n (independent sections)**	5	4	1	3	3
**number of blood vessels**	9	8	n/a	n/a	n/a
**shown in Figure**	3B	3B	not shown	3A	3A
**apical membrane (total µm)**	108.497	129.542	11.511	32.556	112.575
**basal membrane (total µm)**	121.848	152.553	8.145	32.352	103.156
**apical gold (total)**	126	182	88	350	93
**basal gold (total)**	77	484	23	112	201
**apical gold/membrane(µm)**	1.161	1.405	7.645	10.751	0.826
**basal gold/membrane(µm)**	0.631	3.173	2.824	3.462	1.949
**apical gold (%)**	**64.8**	**30.7**	**73.0**	**75.6**	**29.8**
**basal gold (%)**	**35.2**	**69.3**	**27.0**	**24.4**	**70.2**

Mouse hippocampal microvessels or primary rat brain MVECs were processed for cryoimmunogold EM analysis using the indicated antibodies against VEGFR1 or –R2. Representative micrographs of these analyses are shown in Figure 3 A and B. Gold particles were then counted from n independent endothelial monolayer or blood vessel sections. Also shown are the total length of membrane analyzed. Only gold particles within 20 nm of the respective plasma membrane domain were included (Ottersen, 1989).

Full experimental procedures

Magnetic resonance imaging (MRI). Vascular permeability in lungs, brain and eyes was assessed *in vivo* via MRI (Campbell et al., 2012), using a dedicated small rodent 7 T MRI system (http://www.tcd.ie/ Neuroscience/infrastructure/neuroimaging/index.php#7tesla). The effects of VEGF-A or saline control were visualized using T1-weighted MR images (resolution, 0.156 mm×0.156 mm×5 mm; field of view: 20 mm×20 mm×17.9 mm3; matrix; 128×128×30; TR/TE: 500/2.7 ms; flip angle: 30° number of averages: 3; acquisition time: 2 min, 24 s; repetitions: 12) following administration of 100 µl of a 1 in 3 dilution of Gd-DTPA (Gadolinium diethylene-triamine penta-acetic acid), administered via the tail vein. VEGF-A was injected systemically (i.v. into the tail vein) at 3 µg/mouse. Assuming a blood volume of 2-3 ml in an adult mouse (and no rapid loss through tissue permeation or clearance) 3 µg of injected VEGF-A leads to a circulating concentration of 1-1.5 µg/ml. This is more than 1000-fold in excess of physiological plasma levels and also in the range of other reports studying elevated levels of VEGF (e.g. Sun et al., 2012). Alternatively, local injection of VEGF-A in the vitreous of the eye or into the cortex stereotaxically involved an injection of ca. 8 ng VEGF-A. The vitreous volume of a mouse eye is 5.3 µl. Therefore an assumed concentration of 1.5 µg/ml VEGF-A in the eye was comparable to systemic exposure. Quantitative assessment of Gd-DTPA leakage was made on 8-bit, grey scale images by subtracting the contrast intensity in selected regions of interest pre-injection of Gd-DTPA from that post injection of Gd-DTPA. Pseudocolored images using a 16-color look-up table (see Figure 1) were also generated for easier visualization of leakage areas.

***In vivo* permeability measurements in pial microvessels.** The method used in this study, and its theoretical basis, has been described previously (Easton and Fraser, 1994; Easton et al., 1997). The experiments were performed on Wistar rats (aged 25-30 days of either sex) within guidelines directed by The Animals (Scientific Procedures) Act 1986. The animals were anesthetized by an intraperitoneal injection of 60 mg/kg body weight sodium pentobarbital diluted in water (25% w/v), and maintained by supplemental injection of 10% of the original dose when necessary. At the end of the experiment animals were sacrificed by administering an overdose of the anesthetic. In these

weanling animals, which do have a fully developed blood-brain barrier (Butt et al., 1990), the dura and arachnoid was removed, thus exposing the microcirculation of the surface of the brain and leaving it free of any diffusion barrier towards the superfusing solution (artificial cerebrospinal fluid: 110.5 mM NaCl, 4.7 mM KCl, 2.5 mM $CaCl_2$, 1.1 mM KH_2PO_4, 1.25 mM $MgSO_4$, 25 mM $NaHCO_3$ and 15 mM N-[2-hydroxyethyl]piperazine-N,-[2-ethanesulphonic acid], pH 7.4, maintained at 37°C). A low molecular weight fluorescent dye, sulforhodamine B (580 Da), was introduced into the cerebral microcirculation via a bolus injection into the carotid artery, and viewed under 540/25 nm illumination. The fluorescent signal was captured through a microscope via an image-intensifier camera (Hamamatsu). Video microscopy (1 frame/2-5 s) allowed the recording of the time-dependent loss of dye in a single pial venular capillary trapped by a glass-occluding probe. Since the rate of fall of intravascular concentration of a small polar molecule is independent of the hydrostatic pressure, the concentration of dye early in the occlusion, before axial volume flux distorts the uniform axial concentration of dye in the region of measurement, will be $C_t = C_0 e^{-kt}$; where k = 4P/d, and where Ct and C0 are the dye concentrations at time t and time zero, respectively; P is the permeability and d the diameter of the vessel. All experiments were performed within 2 h from the start of surgery, when the preparation is stable without discernible change in baseline permeability (Sarker et al., 2000). Baseline permeability in all experiments was 0.2 -2 x 10^{-6} cm/s. For measurements the flow of superfusing solution was stopped so that a stable pool was formed between the water immersion objective and the brain surface. Dye was injected into the cerebral circulation while the occluding probe was placed over a selected vessel and lowered when the vessel had filled. The dye in the remaining circulation was washed out by the blood flow and did not recirculate. A baseline recording was started immediately for 20 to 60 s. Optionally, second baselines were obtained after 15-min incubation of the brain surface with 10 µM SB202190 or wortmannin. For abluminal application bradykinin, VEGF-A, PlGF-1 or VEGF-E were applied into the pool on the brain surface. The volume of the pool was known, which allowed careful control of final compound concentrations on the surface of the brain. Vasoactive compounds were washed away after ca. 60 s, at which point recordings were also stopped. Luminal bradykinin or VEGF exposure was carried out by injecting the stated concentration of VEGF into the internal carotid artery as a bolus mixed with the dye. Based on

fluorescent intensity measurements this replaced the blood in its entirety, thus the stated concentration was likely to be present in the occluded vessel without much dilution. Image analysis and densitometry was performed using ImageJ 1.45s (NIH).

MVEC isolation. Microvessels were isolated from rat or mouse cortical grey matter, or rat or porcine retinae by collagenase dispase digestion and BSA and percoll density gradient centrifugation (Abbott et al., 1992). Purified vessels were seeded onto collagen IV/fibronectin-coated tissue culture ware or Costar Transwells (3460) at high density (vessels from 6 rat brains or 12 retinae per 40 cm^2 or 3 cm^2, respectively). Cells were grown in EGM2-MV (Lonza) (with 5 µg/ml puromycin during the first 5 days (Perriere et al., 2007)) for 2 to 3 weeks until their TEER plateaued at values above 200 $\Omega.cm^2$. Experiments were performed in 1-week conditioned medium. For experiments analyzing intracellular signaling, cells were switched into EBM2, 0.5% FCS for 24 h prior to treatment and analysis. Low growth factor conditions did not significantly affect barrier properties as judged by TEER.

Immunocytochemistry. MVEC were grown on collagen IV/fibronectin-coated tissue culture ware or 12-mm Costar Transwell filters. Cells were fixed using 3.7% formaldehyde and extracted in acetone (-20°C). Alternatively, they were fixed and permeabilized simultaneously in 80% MeOH, 3.2% formaldehyde, 50 mM Hepes pH 7.4, a method often used for the fixation of C. *elegans* (Otori et al., 2006), which we found to preserve junction integrity and avoid osmotic shock of cell nuclei (Martins et al., 2013). Staining was performed as previously described (Turowski et al., 2004) using antibodies against von Willebrand Factor (Dako), VE-cad (Martins et al., 2013), occludin, Cldn5 (both Invitrogen), VEGFR1 (sc-31173, Santa Cruz Biotechnology), VEGFR2 (ab11939, Abcam) and P-glycoprotein (clone C219).

Immunogold electron microscopy. 10 month-old mice were perfused-fixed with 4% paraformaldehyde (PFA). Brains were isolated, 50 µm slices cut using a vibrating microtome and from these, hippocampi dissected out and then post-fixed for 2 h in 4% PFA. MVECs on Transwell filter inserts were fixed in 4% PFA and 0.1% glutaraldehyde. Fixed samples were processed as previously described (Eden et al., 2010). Briefly, samples were embedded in 12% gelatine in 0.1 M phosphate buffer at pH 7.4. After infusion with 2.3 M sucrose at 4°C overnight, 80-nm sections were cut at -120°C using a cryo-ultramicrotome and collected in 1:1 mixture of 2.3 M sucrose and 2%

methylcellulose. The sections were then stained as previously described (Slot et al., 1991) using antibodies against VEGFR1 (sc-31173)(AF471, R & D) and VEGFR2 (ab11939)(DC101). Samples were viewed on a Jeol 1010 TEM, and images were gathered using a Gatan OriusSC100B charge-coupled device camera. Further image manipulation was performed in Gatan Digital Micrograph and Adobe Photoshop. VEGFR distribution was determined by visual inspection of electron micrographs. Gold particles were considered to be localized luminal (apical) or abluminal (basal), when found within 20 nm of the respective plasma membrane (Ottersen, 1989).

Flux measurement. 4 kDa FITC dextran (1 mg/ml) was added to the apical side of confluent MVECs grown on 12-mm Transwell filters. Samples (50 µl) were removed from the basal chamber (and replaced by fresh medium) at 20-30 min intervals. In standard experiments baseline flux was recorded from untreated cells for 120 min, followed (optionally) by the baseline in the presence of small molecule inhibitors for at least another 60 min. Finally, vasoactive compounds were added and samples were removed for a period of at least 120 min. Fluorescence of samples was plotted against time and permeability changes were determined from linear slope changes before and after addition of the compound (see also Figure 2 A and B).

Transendothelial electrical resistance (TEER). TEER was measured directly with chopstick electrodes (Turowski et al., 2004) and an Evom voltohmmeter (World Precision Instruments). TEER values of empty collagen IV/fibronectin-coated transwells containing MVEC-conditioned medium were subtracted to determine monolayer TEER. Alternatively, TEER was assessed by impedance spectroscopy using cells grown either on 12-mm Transwells and a cellZscope (Nanoanalytics), or on gold electrodes (8-well 8W1E) and ECIS (4000 Hz; Applied Biophysics).

Cytoprotection assays. Caspase activity of MVEC grown in 96-well plates was measured using the Apo-One Homogenous Caspase-3/7 assay kit (Promega).

Cell surface biotinylation. Apical and basal biotinylation was performed using a method adapted from (Gottardi et al., 1995). Briefly, MVEC were grown on 24-mm Transwell filters to confluence and a TEER > 200 $\Omega.cm^2$. Apical biotinylation was performed by two 25-min incubations in 10 mM triethanolamine, 150 mM $NaCl_2$, 2 mM $CaCl_2$, pH 9.0 (BB) and 1.5 mg/ml sulfo-NHS-biotin (Pierce). Under the same conditions basal biotinylation was very weak, presumably because of reduced access

through the filter and the basement membrane (Gottardi et al., 1995). Therefore, apical and basal domains were labeled simultaneously in the presence of EDTA: MVEC monolayers were pre-treated with 1 mM EDTA for 5 min before labeling twice for 25 min in BB supplemented with 1.5 mg/ml sulfo-NHS-biotin (Pierce) and 1 mM EDTA. Cultures were then washed with PBS containing Ca^{2+}, Mg^{2+} and 100 mM glycine before lysis in RIPA buffer (50 mM Tris, pH 7.5, 150 mM NaCl, 1% NP40, 0.5% deoxycholate, and 0.1% SDS) supplemented with protease inhibitors (Roche). Clarified extracts (inputs) were incubated overnight with 500 µl of a 1:1 (vol/vol) slurry of streptavidin-agarose beads (Pierce). The beads were then washed three times with RIPA buffer, and bound proteins eluted by adding 50 mM Tris/Cl, pH 6.8, 2% SDS, 10% glycerol, 100 mM DTT and boiling. Following immunoblotting for VEGF receptors apical and basal signals were quantified by densitometry and normalized against input signals. Basal signal was calculated from the combined apical and basal labeling minus the apical signal.

Western blots. For immunoblot analyses samples were lysed in 50 mM Tris/Cl, pH 6.8, 2% SDS, 10% glycerol, 100 mM DTT, 100 nM calyculin A (50 µl/cm^2 of cells), separated by SDS-PAGE and electrotransfered to nitrocellulose or PVDF. For each series of samples, two identically loaded gels/immunoblots were run for immunodecoration with phospho-specific and total antibodies as previously described (Martinelli et al., 2009). Akt (phospho-S473 and total), p38 (phospho-T180/Y182 and total) and anti-VEGFR2 antibodies were from Cell Signaling (55B11), anti-VEGFR1 from Abcam (Y103).

VEGF receptor localization in retinal and pulmonary vessels. 300 µl of a mixture of anti-VEGFR1 (sc-31173) and -VEGFR2 (DC101) antibodies (each at 6 mg/kg) were slowly (at ca. 60 µl/min) injected into the left ventricle of anesthetized p20 mice (C57 BL/6J). The antibodies were left to circulate and 5 min after the injection animals were perfused with PBS, followed by 4% PFA. Retinae were dissected out, flattened and post-fixed with 4% PFA for 1 h (West et al., 2005). Retinae were washed briefly in PBS and incubated in block (1% FBS, 3% Triton X-100, 0.5% Tween 20, in 2 X PBS) for 2 h at room temperature. For abluminal detection, dissected unfixed retinae of PBS perfused mice were overlaid with anti-VEGFR1 and -VEGFR2 antibodies (20 µg/ml) for 5 min, washed three times briefly with PBS and then fixed in 4% PFA for 1 h. Next, retinae from cardiac injections and

overlays were incubated under gentle agitation with anti-F4/80 (MCA497EL, AbD Serotec) and/or biotinylated isolectin B4 (B1205, Vector Labs) at room temperature overnight. This was followed by incubation with appropriate secondary antibodies, washes and mounting in Mowiol.

Transversal (1-mm) sections of lungs were cut, post-fixed for 1 h in 4% PFA, equilibrated in 30% sucrose/PBS and embedded in OCT compound (Agar) (Powner et al., 2010). 20 µm cryo-sections were cut and collected on superfrost® plus slides (VWR). The slides were air dried for 2 h prior to incubation in section block (1% BSA, 0.5% Triton X-100, PBS) for 1 h at room temperature. The sections were then incubated in secondary antibody mix containing biotinylated isolectin B4 (B1205, Vector Labs). Following washes, sections were mounted in Mowiol.

All samples were analyzed by confocal microscopy on a Zeiss LSM700. Retinal flatmounts were imaged with a pinhole aperture allowing capture of the entire primary plexus depth in a single scan, while sections of lung were analyzed from projections of sequential image stacks.

p38 and Akt signaling *in vivo*. For luminal stimulation 120 µg/kg of VEGF-A, VEGF-E, PlGF-1 or equal volume (100 µl) of saline was injected into the tail vein of anesthetized P23 Wistar rats. For abluminal stimulation, cranial windows were surgically introduced (see above for full description) and VEGFs were added at 100 ng/ml (final concentration) to the liquid pool superfusing the pial microvasculature. 5 min after injection/application, animals were perfused with PBS followed by 4% PFA and the brain and lungs harvested. Approx. 1 mm-thick superficial brain surface sections containing the pial vessels were dissected out. Similarly, ca. 1-mm thick transverse cuts were made through the lungs. Tissues were subsequently post-fixed for 1 h in 4% PFA. Subsequently, tissues were processed for immunohistochemistry as described above using anti-phospho-p38 (T180/Y182) and isolectin B4. Six commercial antibodies against phospho-Akt were tested for staining of such tissue sections. However, none of them produced significant staining in any of the samples, even when additional controls such as insulin treatment/injections were included. Therefore *in vivo* Akt activation was analyzed by immunoblots of retinae: Luminal VEGF stimulation was as above, abluminal stimulation was performed by injecting 100 ng of VEGF-A, VEGF-E, PlGF-1 or equal volume (5 µl) of saline into the vitreous of P23 Wistar rats. After 25 min animals were sacrificed, injected eyes enucleated, the retina lysed in 50 mM Tris/Cl, pH 6.8, 2% SDS, 10% glycerol, 100 mM

DTT, 100 nM calyculin A. Retinal homogenates were syringed through a 26-gauge needle, boiled and analyzed on immunoblots.

Additional materials were rat recombinant VEGF-A(165) (R & D), VEGF-E (Cell Sciences or a kind gift from Prof. Kurt Ballmer-Hofer), PlGF-1 (Peprotech), IGF-nonbinding IGFBP3 (a gift from Dr. Robert Baxter), SB202190, wortmannin, LY294002, SU1498 (all Merck), bradykinin, LPA, thrombin, histamine, puromycin, staurosporine (all Sigma).

Supplemental references

Miller,D.S. (2010). Regulation of P-glycoprotein and other ABC drug transporters at the blood-brain barrier. Trends Pharmacol. Sci. *31*, 246-254.

Otori,M., Karashima,T., and Yamamoto,M. (2006). The Caenorhabditis elegans homologue of deleted in azoospermia is involved in the sperm/oocyte switch. Mol. Biol. Cell *17*, 3147-3155.

Romero,I.A., Radewicz,K., Jubin,E., Michel,C.C., Greenwood,J., Couraud,P.O., and Adamson,P. (2003). Changes in cytoskeletal and tight junctional proteins correlate with decreased permeability induced by dexamethasone in cultured rat brain endothelial cells. Neurosci. Lett. *344*, 112-116.

Sarker,M.H., Hu,D.E., and Fraser,P.A. (2000). Acute effects of bradykinin on cerebral microvascular permeability in the anaesthetized rat. J. Physiol *528 Pt 1*, 177-187.

Weksler,B.B., Subileau,E.A., Perriere,N., Charneau,P., Holloway,K., Leveque,M., Tricoire-Leignel,H., Nicotra,A., Bourdoulous,S., Turowski,P., Male,D.K., Roux,F., Greenwood,J., Romero,I.A., and Couraud,P.O. (2005). Blood-brain barrier-specific properties of a human adult brain endothelial cell line. FASEB J. *19*, 1872-1874.
